# Highly Chemoselective Synthesis of Azaarene-Equipped CF_3_-Tertiary Alcohols under Metal-Free Conditions and Their Fungicidal Activities

**DOI:** 10.1021/acsomega.2c05855

**Published:** 2022-10-10

**Authors:** Bingyi Zhou, Guoyu Yang, Caixia Wang, Lijie Liu, Lijun Shi, Zhenliang Pan, Xiaoming Ji, Lulu Wu, Huayu Zheng, Cuilian Xu, Liangxin Fan

**Affiliations:** † College of Sciences, Henan Agricultural University, Zhengzhou 450002, China; ‡ College of Tobacco Sciences, Henan Agricultural University, Zhengzhou 450002, China; § College of Sciences, Chang’an University, Xi’an 710064, China

## Abstract

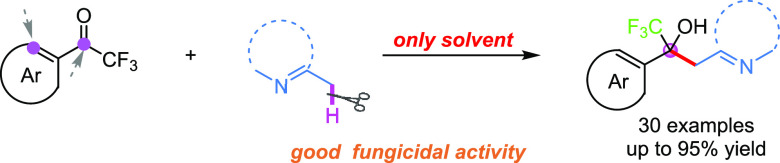

A highly chemoselective reaction between α,β-unsaturated trifluoromethyl ketones with azaarenes under metal-free conditions was carried out, affording a range of valuable azaarene-equipped CF_3_-tertiary alcohols in moderate to excellent yields (up to 95% yield) with good tolerance of functional groups, and their structures were confirmed by NMR, HRMS, and X-ray diffraction for validation. This method features simple reaction conditions (only solvent), high atom- and step-economy, and broad substrate scope. Moreover, most of the target products exhibited promising fungicidal activities, and compound **3al** exhibited 91.65% fungicidal activity against *R. solani*, with an EC_50_ value of 0.18 mg/mL.

## Introduction

**Scheme 1:**
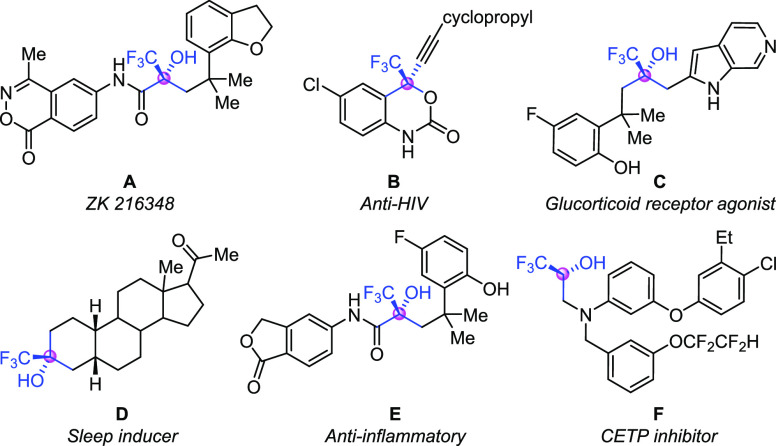
Representative Examples of Drug Molecules with CF_3_-Tertiary Alcohol Motifs

Fluorine-containing molecules are pervasive motifs, and they exhibit broad applications in the field of medicines, agrochemicals, polymers, functional materials, and other chemical industries, owing to the specific characteristics of the fluorine atom, such as high electronegativity, high lipophilicity, good hydrophobicity, metabolic stability, and bioavailability.^1^ Particularly, trifluoromethyl-substituted tertiary alcohols are embedded in a range of pharmaceuticals.^2,3^ For example, compounds **A** and **C** are glucorticoid receptor agonists,^4^ compound **B** is a reverse transcriptase inhibitor of HIV,^5^ compound **D** is a sleep inducer,^6^ compound **E** is an anti-inflammatory reagent,^7^ and compound **F** is a cholesteryl ester transfer protein inhibitor^8^ (Scheme 1). Therefore, the synthesis of these skeletons is highly attractive and a plethora of practical protocols for rapid assembly of CF_3_-substituted tertiary alcohols have been developed in recent years.

**Scheme 2:**
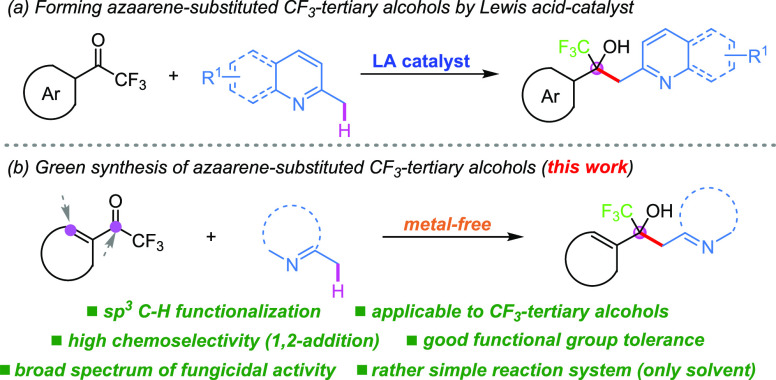
Strategies for the Synthesis of the Azaarene-Equipped CF_3_-Tertiary Alcohols

On the other hand, the direct functionalization of ubiquitous inert C–H bonds has been rapidly developed in past decades, owing to the advantages in terms of atom- and step-economy.^9^ In this regard, C(sp^3^)–H functionalization of azaarenes has emerged as a powerful method for the rapid assembly of functional *N*-heterocycle molecules, which are pervasive motifs found in a series of pharmaceuticals, natural products, and materials.^10^ However, C(sp^3^)–H functionalization of methylquinoline and its derivatives coupled with trifluoromethyl ketones to construct CF_3_-tertiary alcohols have been less explored, and Lewis acid is often required (Scheme 2a).^11,12^ Thus, further development of new efficient functional transformations from the point of more sustainable and environmental chemistry is extremely valuable.^13^

In this context, with our ongoing interest in C–H activation and CF_3_-functionalization chemistry,^14^ we present a novel CF_3_-tertiary alcohol synthesis protocol of trifluoromethyl ketones with azaarenes under metal-free conditions, thus resulting in various ubiquitous CF_3_-tertiary alcohols, which are frequently found in pharmaceutical chemistry (Scheme 2b).^2^

## Results and Discussion

To validate our hypothesis, we began our studies by employing β-(trifluoroacetyl)coumarin **1a** and 2-methylquinoline **2a** as model substrates to explore the reaction conditions, and the results are summarized in Table 1. Initially, a comprehensive screening of reaction solvents was carried out (entries 1–11). To our delight, all the cases such as DMF, DMSO, EtOH, acetone, EA, DCE, CH_3_CN, 1,4-dioxane, toluene, THF, and *n*-hexane were feasible to perform the titled conversion, as shown in Scheme 2, and the CH_3_CN solvent was identified as the best choice, affording anticipated product **3aa** in 82% yield (entry 7). Further adjusting the reaction temperature, neither increase nor decrease had a positive effect (entries 12–13). Gratifyingly, it was found that the ratio of two substrates could affect the reaction performance dramatically (entries 14–15), improving the yield of **3aa** to 93% when altering the ratio of **1a:2a** to 1:2 (entry 15). Additionally, the potential 1,4-addition byproduct was not discovered during the whole optimization reaction conditions. Accordingly, the optimized reaction conditions were determined as follows: **1a** (0.2 mmol), **2a** (0.4 mmol), and CH_3_CN (2.0 mL) at 90 °C under an air atmosphere for 9 h.

**Table 1:**
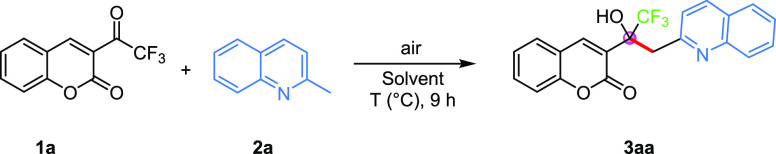
Optimization of the Reaction Conditions^a^

entry	solvent	**2a** (mmol)	*T* (°C)	yield^b^ (%)
1	DMF	0.2	90	55
2	DMSO	0.2	90	35
3	EtOH	0.2	90	27
4	Acetone	0.2	90	49
5	EA	0.2	90	31
6	DCE	0.2	90	24
7	CH_3_CN	0.2	90	82
8	1,4-dioxane	0.2	90	57
9	Toluene	0.2	90	63
10	THF	0.2	90	50
11	*n*-hexane	0.2	90	48
12	CH_3_CN	0.2	70	68
13	CH_3_CN	0.2	110	81
14	CH_3_CN	0.3	90	86
**15**	**CH_3_CN**	**0.4**	**90**	**93**

a All reactions were performed with **1a** (0.2 mmol) and **2a** in solvent (2.0 mL) under an air atmosphere at 90 °C for 9 h.

b Isolated yield. DMF = *N,N*-dimethylformamide, DMSO = dimethylsulfoxide, EA = ethyl acetate, DCE = 1,2-dichloroethane, and THF = tetrahydrofuran.

With efficient protocols in hand, the scope with respect to the azaarenes was first examined. The results indicated that various azaarenes are well tolerated, and the reaction of azaarenes **2b-r** with **1a** underwent the envisioned pathways to deliver the CF_3_-tertiary alcohols **3ab-ar** in 41–93% yields (Table 2). For instance, diversified functionalization of the 4-, 6-, 7-, and 8-positions of **2a** with electron-donating groups (EDGs) such as methoxy (**2e**) and ethoxy (**2f**) and electron-withdrawing groups (EWGs) such as fluoro (**2g**, **2k**), chloro (**2b**, **2h**), bromo (**2c**, **2i**, **2l**), and ester (**2d**, **2j**) is tolerated with good yields. Moreover, methylquinoline-bearing methyl at C_4_ also proved to be feasible and afforded the anticipated CF_3_-tertiary alcohol product **3am** in 46% yield. Notably, the established reaction conditions also led to anticipated product **3an** when 1-methylisoquinoline (**2n**) was used as a coupling partner. After examining the tolerance of methylquinoline under the optimized conditions as shown in Table 1, we turned our attention on the various methyl *N*-heterocycles (**2o–q**) with **1a**. To our delight, various methyl *N*-heterocycles such as quinoxaline (**2o**), quinazolinone (**2p**), and benzothiazole (**2q**) were reacted with **1a** smoothly, which produced various 1-(β-coumarinyl)-1-(β-heterocyclyl)trifluoroethanols in moderate to good yields. Furthermore, 2-methylpyridine (**2r**) was found to participate in the established reaction, giving **3ar** in 44% yield.

**Table 2:**
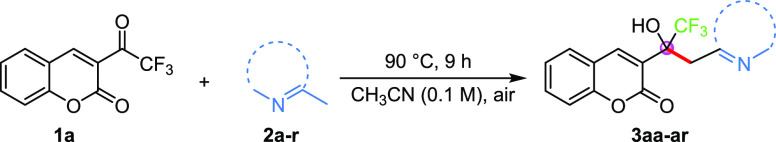

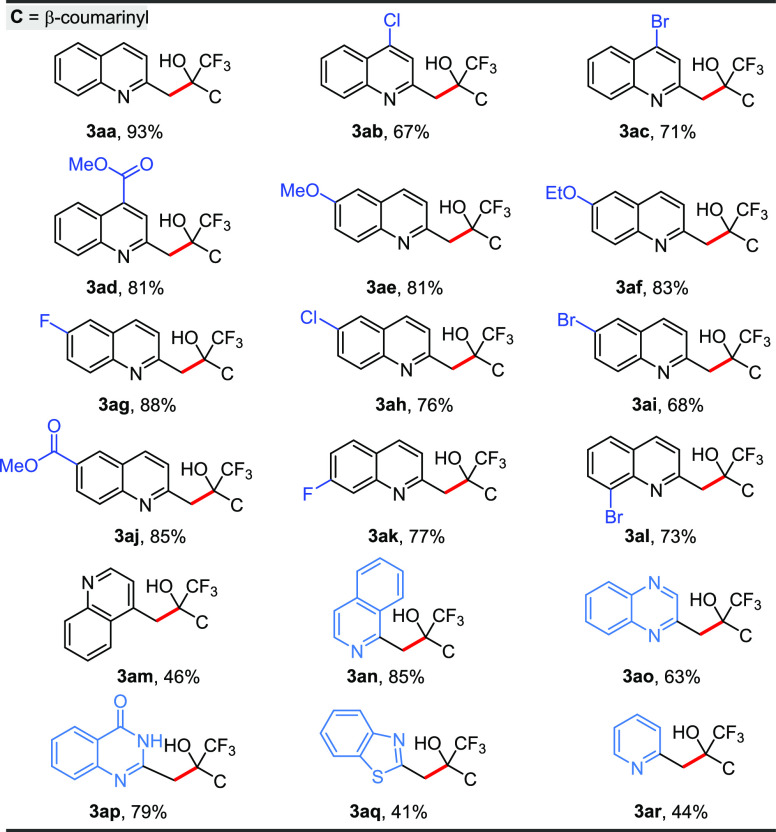
Scope of Azaarenes^a^

a All reactions were performed with **1a** (0.2 mmol) and **2a–r** (0.4 mmol) in 2.0 mL CH_3_CN under an air atmosphere at 90 °C for 9 h; Isolated yields are given.

To further investigate the scope of this reaction, diversified trifluoromethyl ketones were evaluated (Table 3). Overall, a series of trifluoroacetylcoumarins or trifluoroacetylbenzene equipped with EDGs and EWGs are well tolerated, affording the anticipated trifluoromethyl tertiary alcohols **3ba–ma** in 52–95% yields. Specifically, C_6_ sites with methyl (**1b**)-, fluoro (**1c**)-, chloro (**1d**)-, and bromo (**1e**)-modified CF_3_-coumarin ketones reacted smoothly with **2a**, delivering desired products with moderate to excellent yields (67–95%). Particularly, a sensitive but versatile iodine group (**1f**) was also well compatible, which is often used in transition metal-catalyzed cross coupling transformations. Next, C_7_ and C_8_ sites with strong EDGs such as methoxy (**1g**, **1h**) and ethoxy (**1i**) are well tolerated, giving rise to anticipated **3ga–ia** in 83–88% yields. Notably, disubstituted substrates (**1j**) and (**1k**) were well compatible and delivered the corresponding adduct products in 84 and 89% yields, respectively. Meanwhile, the structure of **3ja** was also determined by analogy on the basis of X-ray (CCDC 2179504). Gratifyingly, the fused-ring system **1l** also reacted smoothly, generating tertiary alcohol product **3la** in 89% yield. Finally, it should be mentioned that trifluoroacetophenone (**1m**) could be subjected as the electrophilic reagent to produce **3ma** with moderate yield (52%) in an established metal-free system, which is quite different from previous reports that often rely on Lewis acid catalysis, such as iron salt, indium salt, and so on.^11,12^

**Table 3:**
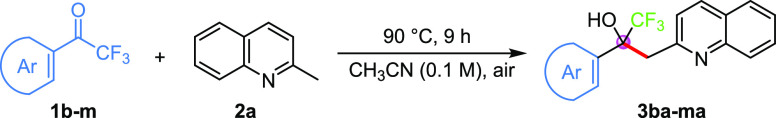

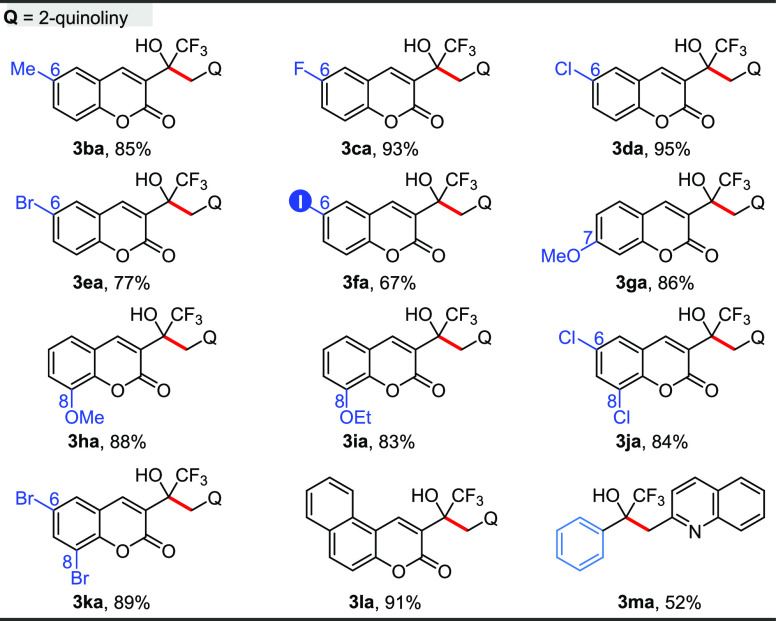
Scope of Trifluoromethyl Ketones^a^

a All reactions were performed with **1b–m** (0.2 mmol) and **2a** (0.4 mmol) in 2.0 mL CH_3_CN under an air atmosphere at 90 °C for 9 h. Isolated yields are given.

**Scheme 3:**
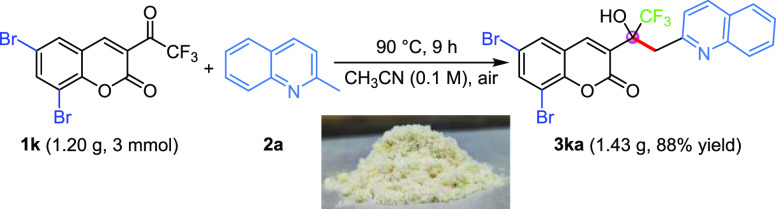
Gram-Scale Reaction

To further showcase the potential utility of this newly established method, a larger-scale (3 mmol) experiment was carried out between **1k** and **2a** (Scheme 3), which delivered the desired product **3ka** with comparable yield (1.43 g, 88% yield).

**Scheme 4:**
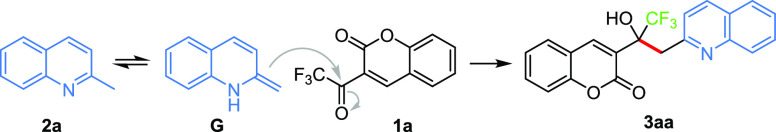
Proposed Mechanism

Based on the abovementioned results and previous reports,^11^ a plausible reaction pathway for the conversion of trifluoromethyl ketone and azaarene to CF_3_-tertiary alcohols under metal-free conditions is outlined in Scheme 4. Initially, 2-methylquinoline **2a** was converted to its enamine species **G**, and then, the enamide species **G** underwent nucleophilic addition to β-(trifluoroacetyl)coumarin **1a**; further protonation could provide the expected azaarene-equipped CF_3_-tertiary alcohol **3aa**.

**Figure 1:**
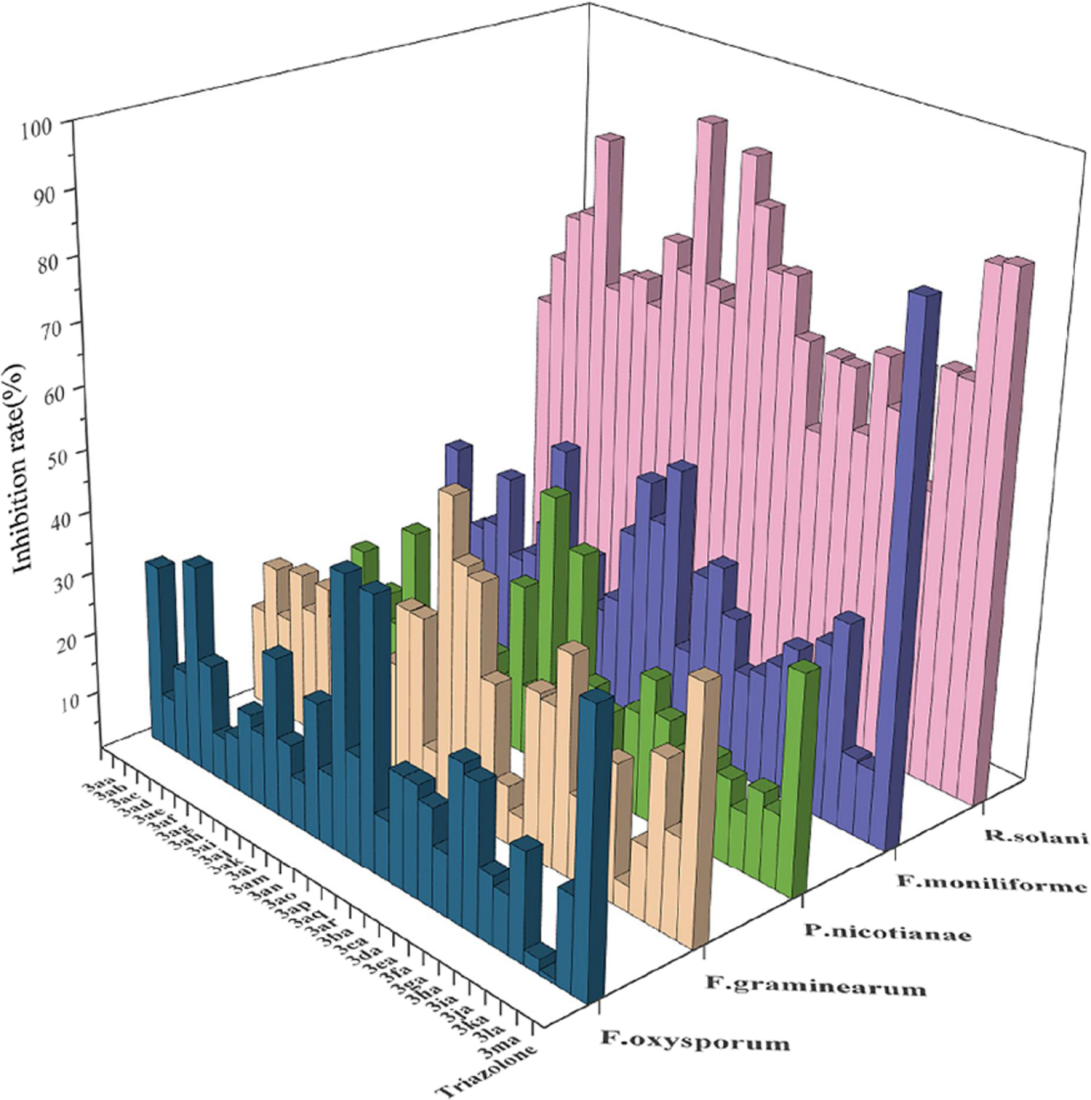
Antifungal activities of the CF_3_-tertiary alcohols **3aa–3ma**.

To evaluate the biological activity of the newly prepared azaarene-equipped CF_3_-tertiary alcohols, preliminary antifungal activity against *F. oxysporum*, *F. graminearum*, *P. nicotianae*, *F. moniliforme*, and *R. solani* with target compounds **3aa–ma** was performed based on the early reference,^15^ and the results are summarized in Figure 1. Overall, most of the desired CF_3_-tertiary alcohol products **3aa–ma** showed fungicidal activities against the abovementioned five fungi. First, the inhibitor rate of the model product **3aa** against *F. oxysporum* was 29.84% at 0.5 mg/mL. The inhibitor rate was improved to 44.32 and 45.11% when compounds **3aq** and **3ao** were used, respectively. Next, compounds **3aa–ma** against *F. graminearum*, *P. nicotianae*, *F. moniliforme*, and *R. solani* were tested. Gratifyingly, the antifungal activity against *R. solani* was obviously better compared to other three fungi, with a moderate to good inhibitor rate. Particularly, compound **3al** displayed good (91.65%) in vitro fungicidal activity, with the median effective concentration (EC_50_) value of 0.18 mg/mL.

## Conclusions

In conclusion, we have developed a novel reaction for the synthesis of azaarene-equipped CF_3_-tertiary alcohols through addition of azaarenes to CF_3_-ketones under metal-free conditions. The azaarenes, including quinolones, isoquinoline, quinoxaline, quinazolinone, and benzothiazole, were used as coupling partners. The corresponding CF_3_-tertiary alcohol products were obtained in excellent yields (up to 95% yield) with high chemoselectivity (only 1,2-addition). Compared to previous reports, this established reaction features simple reaction conditions (only solvent), high atom- and step-economy, and broad substrate scope. Moreover, most of the synthesized compounds displayed promising fungicidal activities, and compound **3al** exhibited 91.65% fungicidal activity against *R. solani*, with an EC_50_ value of 0.18 mg/mL. Further studies on the synthesis of novel CF_3_-tertiary alcohol scaffolds with green methods and their fungicidal activities are ongoing in our laboratories.

## Experimental Section

### General Information

All reactions were carried out under an air atmosphere. All reagents were used as received unless otherwise noted. Analytical thin-layer chromatography was performed with 0.25 mm-coated commercial silica gel plates (TLC Silica Gel 60 F_254_); the developed chromatogram was visualized using fluorescence. Flash chromatography was performed with silica gel (200–300 mesh). Proton nuclear magnetic resonance (^1^H NMR) data were acquired at 400 MHz on a Bruker Ascend spectrometer. Chemical shifts are reported in delta (δ) units, in parts per million (ppm) downfield from tetramethylsilane. Splitting patterns are designated as s, singlet; d, doublet; t, triplet; and m, multiplet. Coupling constants *J* are quoted in Hz. Carbon-13 nuclear magnetic resonance (^13^C NMR) data were acquired at 100 MHz on a Bruker Ascend 400 spectrometer. Chemical shifts are reported in ppm relative to the center line of a triplet at 77.0 ppm for chloroform-d and the center line of a septet at 44.0 ppm for DMSO-*d*_6_. Fluorine nuclear magnetic resonance (^19^F NMR) data were acquired at 376 MHz on a Bruker Ascend 400 spectrometer, and chemical shifts are reported relative to interstandard CFCl_3_ at 0.0 ppm. Mass spectra were acquired on a Bruker Daltonics MicroTof-Q II mass spectrometer.

### General Procedure for the Preparation of Azaarene-Equipped CF_3_-Tertiary Alcohols

A 10.0 mL vial equipped with a stirring bar was charged with trifluoromethyl ketones **1** (0.2 mmol, 1.0 equiv), azaarenes **2** (0.4 mmol, 2.0 equiv), and CH_3_CN (2.0 mL). The vial was sealed with a Teflon screw cap, and the reaction mixture was heated at 90 °C for 9 h under pressure. After the reaction vessel was cooled to room temperature, the crude reaction mixture was filtered with celite and washed with DCM. The solvent was removed under reduced pressure. Then, the residue was purified by silica gel column chromatography to afford the desired product **3**.

#### 3-(1,1,1-Trifluoro-2-hydroxy-3-(quinolin-2-yl)propan-2-yl)-2*H*-chromen-2-one (**3aa**)

(PE:EA = 5:1, *R*_f_ = 0.37, white solid, mp = 91.7–92.3 °C, 93% yield). ^1^H NMR (400 MHz, Chloroform-*d*) δ 8.64 (s, 1H), 8.47 (s, 1H), 8.10 (d, *J* = 8.4 Hz, 1H), 7.93 (d, *J* = 8.5 Hz, 1H), 7.76 (d, *J* = 8.2 Hz, 1H), 7.67 (t, *J* = 7.7 Hz, 1H), 7.51–7.44 (m, 4H), 7.23–7.19 (m, 2H), 4.56 (d, *J* = 14.7 Hz, 1H), 3.73 (d, *J* = 14.7 Hz, 1H). ^13^C NMR (100 MHz, Chloroform-*d*) δ 159.2, 158.3, 154.0, 146.1, 137.8, 132.4, 130.2, 128.7, 128.2, 127.8, 127.1, 126.7, 126.5, 124.8, 124.6 (q, *J* = 284.0 Hz), 124.5, 123.1, 118.7, 116.2, 76.8 (q, *J* = 30.0 Hz), 37.4. ^19^F NMR (376 MHz, Chloroform-*d*) δ −79.98. IR (KBr): 3421, 2837, 2810, 1653, 1586, 1373, 1357, 783, 770 cm^–1^. HRMS (ESI) *m/z* calculated for C_21_H_15_F_3_NO_3_ [M + H]^+^ 386.1004, found 386.1007.

#### 3-(3-(4-Chloroquinolin-2-yl)-1,1,1-trifluoro-2-hydroxypropan-2-yl)-2*H*-chromen-2-one (**3ab**)

(PE:EA = 5:1, *R*_f_ = 0.35, white solid, mp = 140.8–141.2 °C, 65% yield). ^1^H NMR (400 MHz, Chloroform-*d*) δ 8.48 (s, 1H), 8.30 (s, 1H), 8.16 (d, *J* = 8.5 Hz, 1H), 7.93 (d, *J* = 8.4 Hz, 1H), 7.72 (t, *J* = 8.4 Hz, 1H), 7.60 (t, *J* = 8.3 Hz, 2H), 7.51–7.46 (m, 2H), 7.25–7.21 (m, 2H), 4.52 (d, *J* = 14.7 Hz, 1H), 3.70 (d, *J* = 14.7 Hz, 1H). ^13^C NMR (100 MHz, Chloroform-*d*) δ 159.3, 158.0, 154.0, 147.0, 146.2, 144.2, 132.5, 131.0, 128.7, 128.5, 127.6, 125.9, 125.4, 124.6, 124.5 (q, *J* = 284.0 Hz), 124.3, 123.0, 118.6, 116.3, 76.7 (q, *J* = 30.1 Hz), 37.5. ^19^F NMR (376 MHz, Chloroform-*d*) δ −80.06. IR (KBr): 3436, 2849, 2820, 2722, 1743, 1673, 1641, 1597, 1391, 1378, 1346, 784, 768 cm^–1^. HRMS (ESI) *m/z* calculated for C_21_H_14_ClF_3_NO_3_ [M + H]^+^ 420.0614, found 420.0602.

#### 3-(3-(4-Bromoquinolin-2-yl)-1,1,1-trifluoro-2-hydroxypropan-2-yl)-2*H*-chromen-2-one (**3ac**)

(PE:EA = 3:1, *R*_f_ = 0.45, white solid, mp = 138.7–139.5 °C, 71% yield). ^1^H NMR (400 MHz, Chloroform-*d*) δ 8.47 (s, 1H), 8.29 (s, 1H), 8.11 (d, *J* = 8.5 Hz, 1H), 7.91 (d, *J* = 8.4 Hz, 1H), 7.79 (s, 1H), 7.73–7.69 (m, 1H), 7.59 (d, *J* = 5.6 Hz, 1H), 7.48 (dd, *J* = 10.5, 7.8 Hz, 2H), 7.25–7.19 (m, 2H), 4.50 (d, *J* = 14.7 Hz, 1H), 3.70 (d, *J* = 14.8 Hz, 1H). ^13^C NMR (100 MHz, Chloroform-*d*) δ 159.3, 157.9, 154.0, 146.7, 146.2, 135.7, 132.5, 131.0, 128.7, 128.6, 127.9, 126.9, 126.8, 126.6, 124.6, 124.5 (q, *J* = 286.1 Hz), 124.3, 118.6, 116.3, 76.7 (q, *J* = 30.0 Hz), 37.3. IR (KBr): 3444, 2847, 2816, 2735, 1631, 1595, 1391, 1352, 1269, 1183, 791, 763, 739 cm^–1^. ^19^F NMR (376 MHz, Chloroform-*d*) δ −80.05. HRMS (ESI) *m/z* calculated for C_21_H_14_BrF_3_NO_3_ [M + H]^+^ 464.0109, found 464.0108.

#### Methyl 2-(3,3,3-Trifluoro-2-hydroxy-2-(2-oxo-2*H*-chromen-3-yl)propyl)quinoline-4-carboxylate (**3ad**)

(PE:EA = 5:1, *R*_f_ = 0.33, white solid, mp = 185.3–186.5 °C, 81% yield). ^1^H NMR (400 MHz, Chloroform-*d*) δ 8.69 (d, *J* = 8.7 Hz, 1H), 8.48 (s, 1H), 7.98 (s, 2H), 7.70 (d, *J* = 7.0 Hz, 1H), 7.61–7.57 (m, 1H), 7.48 (dd, *J* = 14.3, 8.0 Hz, 2H), 7.22 (t, *J* = 7.4 Hz, 3H), 4.58 (d, *J* = 14.9 Hz, 1H), 4.02 (s, 3H), 3.77 (d, *J* = 14.8 Hz, 1H). ^13^C NMR (100 MHz, Chloroform-*d*) δ 166.0, 159.3, 157.7, 153.9, 147.2, 146.2, 136.2, 132.5, 130.4, 128.7, 128.6, 128.2, 125.7, 124.6, 124.5 (q, *J* = 286.2 Hz), 124.2, 124.1, 118.6, 116.2, 76.5 (q, *J* = 29.9 Hz), 52.9, 37.7. ^19^F NMR (376 MHz, Chloroform-*d*) δ −80.04. IR (KBr): 3425, 2844, 2810, 2755, 1650, 1623, 1583, 1388, 1349, 1265, 789, 756 cm^–1^. HRMS (ESI) *m/z* calculated for C_23_H_17_F_3_NO_5_ [M + H]^+^ 444.1059, found 444.1017.

#### 3-(1,1,1-Trifluoro-2-hydroxy-3-(6-methoxyquinolin-2-yl)propan-2-yl)-2*H*-chromen-2-one (**3ae**)

(PE:EA = 5:1, *R*_f_ = 0.31, white solid, mp = 185.5–186.7 °C, 81% yield). ^1^H NMR (400 MHz, Chloroform-*d*) δ 8.65 (s, 1H), 8.60 (s, 1H), 8.45 (s, 1H), 7.98 (d, *J* = 8.4 Hz, 1H), 7.81 (d, *J* = 9.2 Hz, 1H), 7.46 (t, *J* = 7.9 Hz, 2H), 7.41 (d, *J* = 8.4 Hz, 1H), 7.31 (dd, *J* = 9.2, 2.7 Hz, 1H), 7.21 (ddt, *J* = 7.6, 4.1, 2.0 Hz, 2H), 7.00 (d, *J* = 2.8 Hz, 1H), 4.51 (d, *J* = 14.6 Hz, 1H), 3.88 (s, 3H), 3.68 (d, *J* = 14.6 Hz, 1H). ^13^C NMR (100 MHz, Chloroform-*d*) δ 159.2, 157.8, 155.4, 154.0, 146.0, 142.3, 136.4, 132.3, 129.5, 128.6, 128.2, 124.9, 124.6 (q, *J* = 286.2 Hz), 124.4, 123.3, 123.0, 118.7, 116.1, 105.0, 76.8 (q, *J* = 29.9 Hz), 55.6, 37.1. ^19^F NMR (376 MHz, Chloroform-*d*) δ −79.86. IR (KBr): 3437, 2847, 2735, 2701, 1673, 1650, 1603, 1391, 1347, 797, 768, 750 cm^–1^. HRMS (ESI) *m/z* calculated for C_22_H_17_F_3_NO_4_ [M + H]^+^ 416.1110, found 416.1128.

#### 3-(3-(6-Ethoxyquinolin-2-yl)-1,1,1-trifluoro-2-hydroxypropan-2-yl)-2*H*-chromen-2-one (**3af**)

(PE:EA = 5:1, *R*_f_ = 0.38, white solid, mp = 190.9–191.6 °C, 83% yield). ^1^H NMR (400 MHz, Chloroform-*d*) δ 8.60 (s, 1H), 8.44 (s, 1H), 7.96 (d, *J* = 8.4 Hz, 1H), 7.80 (d, *J* = 9.2 Hz, 1H), 7.50–7.44 (m, 2H), 7.40 (d, *J* = 8.4 Hz, 1H), 7.31 (dd, *J* = 9.2, 2.7 Hz, 1H), 7.25–7.17 (m, 2H), 6.98 (d, *J* = 2.7 Hz, 1H), 4.50 (d, *J* = 14.6 Hz, 1H), 4.10 (dd, *J* = 7.0, 2.9 Hz, 2H), 3.67 (d, *J* = 14.6 Hz, 1H), 1.45 (t, *J* = 7.0 Hz, 3H). ^13^C NMR (100 MHz, Chloroform-*d*) δ 159.2, 157.2, 155.2, 154.0, 146.0, 142.2, 136.4, 132.3, 129.5, 128.6, 128.2, 126.0, 124.9, 124.6 (q, *J* = 286.1 Hz), 124.4, 123.2, 118.7, 116.1, 105.7, 76.8 (q, *J* = 29.3 Hz), 63.9, 37.1, 14.7. ^19^F NMR (376 MHz, Chloroform-*d*) δ −79.91. IR (KBr): 3450, 2852, 2806, 2732, 1657, 1597, 1383, 1352, 1269, 786, 763 cm^–1^. HRMS (ESI) *m/z* calculated for C_23_H_19_F_3_NO_4_ [M + H]^+^ 430.1266, found 430.1288.

#### 3-(1,1,1-Trifluoro-3-(6-fluoroquinolin-2-yl)-2-hydroxypropan-2-yl)-2*H*-chromen-2-one (**3ag**)

(PE:EA = 5:1, *R*_f_ = 0.30, white solid, mp = 121.9–122.8 °C, 63% yield). ^1^H NMR (400 MHz, Chloroform-*d*) δ 8.46 (s, 1H), 8.34 (s, 1H), 8.06 (d, *J* = 8.5 Hz, 1H), 7.91 (s, 1H), 7.46 (dt, *J* = 14.5, 7.4 Hz, 4H), 7.38 (dd, *J* = 8.7, 2.8 Hz, 1H), 7.22 (t, *J* = 7.1 Hz, 2H), 4.55 (d, *J* = 14.6 Hz, 1H), 3.71 (d, *J* = 14.7 Hz, 1H). ^13^C NMR (100 MHz, Chloroform-*d*) δ 161.7, 159.3, 157.6, 154.0, 146.2, 137.1, 132.5, 130.7, 128.7, 127.8, 127.7, 124.6, 124.5 (q, *J* = 285.8 Hz), 123.9, 120.3, 118.6, 116.2, 111.0, 110.8, 76.7 (q, *J* = 30.0 Hz), 37.5. ^19^F NMR (376 MHz, Chloroform-*d*) δ −79.99, −112.81. IR (KBr): 3444, 2848, 2821, 2724, 1743, 1673, 1639, 1605, 1089, 1378, 1347, 786, 769 cm^–1^. HRMS (ESI) *m/z* calculated for C_21_H_14_F_4_NO_3_ [M + H]^+^ 404.0910, found 404.0924.

#### 3-(3-(6-Chloroquinolin-2-yl)-1,1,1-trifluoro-2-hydroxypropan-2-yl)-2*H*-chromen-2-one (**3ah**)

(PE:EA = 5:1, *R*_f_ = 0.39, white solid, mp = 161.2–162.7 °C, 76% yield). ^1^H NMR (400 MHz, Chloroform-*d*) δ 8.45 (s, 1H), 8.27 (s, 1H), 8.02 (d, *J* = 8.5 Hz, 1H), 7.86 (d, *J* = 9.0 Hz, 1H), 7.74 (d, *J* = 2.3 Hz, 1H), 7.60 (dd, *J* = 9.0, 2.3 Hz, 1H), 7.54–7.46 (m, 3H), 7.22 (t, *J* = 7.3 Hz, 2H), 4.54 (d, *J* = 14.7 Hz, 1H), 3.72 (d, *J* = 14.7 Hz, 1H). ^13^C NMR (100 MHz, Chloroform-*d*) δ 159.2, 158.6, 154.0, 146.1, 144.6, 136.7, 132.4, 131.0, 129.8, 128.7, 127.7, 126.4, 125.9, 124.6, 124.5 (q, *J* = 286.6 Hz), 124.0, 123.1, 118.6, 116.2, 76.7 (q, *J* = 30.0 Hz), 37.6. ^19^F NMR (376 MHz, Chloroform-*d*) δ −80.01. IR (KBr): 3436, 2849, 2813, 1633, 1594, 1589, 1378, 1349, 781, 770 cm^–1^. HRMS (ESI) *m/z* calculated for C_21_H_14_ClF_3_NO_3_ [M + H]^+^ 420.0614, found 420.0598.

#### 3-(3-(6-Bromoquinolin-2-yl)-1,1,1-trifluoro-2-hydroxypropan-2-yl)-2*H*-chromen-2-one (**3ai**)

(PE:EA = 5:1, *R*_f_ = 0.37, white solid, mp = 180.3–180.9 °C, 68% yield). ^1^H NMR (400 MHz, Chloroform-*d*) δ 8.45 (s, 1H), 8.24 (s, 1H), 8.02 (d, *J* = 8.5 Hz, 1H), 7.92 (s, 1H), 7.79 (d, *J* = 9.2 Hz, 1H), 7.73 (d, *J* = 6.9 Hz, 1H), 7.53–7.46 (m, 3H), 7.23 (t, *J* = 7.4 Hz, 2H), 4.54 (d, *J* = 14.7 Hz, 1H), 3.70 (d, *J* = 14.6 Hz, 1H). ^13^C NMR (100 MHz, Chloroform-*d*) δ 159.4, 158.9, 154.1, 146.7, 144.2, 137.4, 133.8, 132.7, 130.6, 128.9, 128.4, 124.8, 124.7, 124.6 (q, *J* = 286.2 Hz), 124.2, 120.7, 118.7, 116.4, 76.5 (q, *J* = 30.3 Hz), 37.8. ^19^F NMR (376 MHz, Chloroform-*d*) δ −79.37. IR (KBr): 3431, 2852, 2800, 1631, 1615, 1589, 1390, 1363, 1348, 780, 765, 736 cm^–1^. HRMS (ESI) *m/z* calculated for C_21_H_14_BrF_3_NO_3_ [M + H]^+^ 464.0109, found 464.0101.

#### Methyl-2-(3,3,3-trifluoro-2-hydroxy-2-(2-oxo-2*H*-chromen-3-yl)propyl)quinoline-6-carboxylate (**3aj**)

(PE:EA = 5:1, *R*_f_ = 0.25, white solid, mp = 198.1–199.8 °C, 85% yield). ^1^H NMR (400 MHz, Chloroform-*d*) δ 8.52 (d, *J* = 1.9 Hz, 1H), 8.47 (s, 1H), 8.25 (dd, *J* = 8.9, 1.9 Hz, 1H), 8.21 (d, *J* = 8.5 Hz, 1H), 7.96 (d, *J* = 8.8 Hz, 1H), 7.55 (d, *J* = 8.5 Hz, 1H), 7.48 (dd, *J* = 12.7, 7.3 Hz, 2H), 7.22 (t, *J* = 7.4 Hz, 8H), 4.58 (d, *J* = 14.8 Hz, 1H), 3.99 (s, 1H), 3.96 (s, 3H), 3.75 (d, *J* = 14.8 Hz, 1H). ^13^C NMR (100 MHz, Chloroform-*d*) δ 166.3, 160.7, 159.2, 154.0, 148.0, 146.2, 138.9, 132.5, 130.9, 129.7, 128.7, 128.5, 128.2, 126.3, 124.6, 124.5 (q, *J* = 285.9 Hz), 124.4, 124.0118.6, 116.2, 76.7 (q, *J* = 30.0 Hz), 52.5, 37.9. ^19^F NMR (376 MHz, Chloroform-*d*) δ −80.07. IR (KBr): 3429, 2850, 2808, 2735, 1667, 1631, 1592, 1389, 1350, 1269, 794, 747 cm^–1^. HRMS (ESI) *m/z* calculated for C_23_H_17_F_3_NO_5_ [M + H]^+^ 444.1059, found 444.1073.

#### 3-(1,1,1-Trifluoro-3-(7-fluoroquinolin-2-yl)-2-hydroxypropan-2-yl)-2*H*-chromen-2-one (**3ak**)

(PE:DCM = 1:1, *R*_f_ = 0.25, white solid, mp = 141.6–142.2 °C, 62% yield). ^1^H NMR (400 MHz, Chloroform-*d*) δ 8.47 (s, 1H), 8.33 (s, 1H), 8.10 (d, *J* = 8.4 Hz, 1H), 7.76 (dd, *J* = 9.0, 6.0 Hz, 1H), 7.55 (d, *J* = 8.2 Hz, 1H), 7.51 (d, *J* = 8.0 Hz, 1H), 7.46 (dd, *J* = 8.2, 5.0 Hz, 2H), 7.29 (dd, *J* = 8.6, 2.6 Hz, 1H), 7.27–7.18 (m, 3H), 4.55 (d, *J* = 14.6 Hz, 1H), 3.72 (d, *J* = 14.7 Hz, 1H). ^13^C NMR (100 MHz, Chloroform-*d*) δ 164.6, 162.1, 159.4, 159.2, 154.0, 146.1, 137.7, 132.4, 130.0, 129.9, 128.7, 124.6, 124.5 (q, *J* = 286.1 Hz,), 124.2, 122.5, 118.6, 117.4, 116.2, 112.0, 76.7 (q, *J* = 30.1 Hz), 37.6. ^19^F NMR (376 MHz, Chloroform-*d*) δ −80.00, −87.20. IR (KBr): 3429, 2850, 2810, 2743, 1634, 1605, 1383, 1350, 1263, 789, 768 cm^–1^. HRMS (ESI) *m/z* calculated for C_21_H_14_F_4_NO_3_ [M + H]^+^ 404.0910, found 404.0927.

#### 3-(3-(8-Bromoquinolin-2-yl)-1,1,1-trifluoro-2-hydroxypropan-2-yl)-2*H*-chromen-2-one (**3al**)

(PE:EA = 3:1, *R*_f_ = 0.45, white solid, mp = 155.6–156.4 °C, 73% yield). ^1^H NMR (400 MHz, Chloroform-*d*) δ 8.61 (s, 1H), 8.11 (d, *J* = 8.4 Hz, 1H), 7.95 (d, *J* = 6.3 Hz, 1H), 7.72 (d, *J* = 8.1 Hz, 1H), 7.55–7.49 (m, 2H), 7.48–7.43 (m, 1H), 7.34 (t, *J* = 7.8 Hz, 1H), 7.22 (t, *J* = 7.6 Hz, 2H), 7.18 (d, *J* = 8.3 Hz, 1H), 4.66 (d, *J* = 15.2 Hz, 1H), 3.75 (d, *J* = 15.2 Hz, 1H). ^13^C NMR (100 MHz, Chloroform-*d*) δ 159.3, 159.2, 153.9, 146.5, 143.2, 138.1, 133.4, 132.3, 128.7, 128.3, 127.6, 127.1, 124.7, 124.5 (q, *J* = 285.6 Hz), 124.4, 123.8, 123.6, 118.7, 116.1, 76.8 (q, *J* = 30.2 Hz), 37.6. ^19^F NMR (376 MHz, Chloroform-*d*) δ −80.01. IR (KBr): 3416, 3090, 3048, 3032, 2659, 1717, 1634, 1597, 1378, 1357, 1196, 1120, 945, 768, 729 cm^–1^. HRMS (ESI) *m/z* calculated for C_21_H_14_BrF_3_NO_3_ [M + H]^+^ 464.0109, found 464.0108.

#### 3-(1,1,1-Trifluoro-2-hydroxy-3-(quinolin-4-yl)propan-2-yl)-2*H*-chromen-2-one (**3am**)

(PE:EA = 3:1, *R*_f_ = 0.45, white solid, mp = 183.8–184.6 °C, 46% yield). ^1^H NMR (400 MHz, DMSO-*d*_6_) δ 8.68 (d, *J* = 4.5 Hz, 1H), 8.51 (s, 1H), 8.26 (d, *J* = 7.2 Hz, 1H), 7.97 (d, *J* = 9.7 Hz, 1H), 7.84 (d, *J* = 7.9 Hz, 1H), 7.74 (d, *J* = 6.9 Hz, 1H), 7.65–7.60 (m, 2H), 7.56 (s, 1H), 7.50 (d, *J* = 4.6 Hz, 1H), 7.38–7.33 (m, 2H), 4.60 (d, *J* = 15.7 Hz, 1H), 3.80 (d, *J* = 15.8 Hz, 1H). ^13^C NMR (100 MHz, DMSO-*d*_6_) δ 158.6, 153.7, 150.1, 148.2, 145.8, 141.8, 133.4, 130.0, 129.8, 129.5, 128.2, 126.9, 125.8 (q, *J* = 289.5 Hz), 125.3, 124.8, 123.6, 121.9, 118.5, 116.3, 76.3 (q, *J* = 28.6 Hz), 33.0. ^19^F NMR (376 MHz, DMSO-*d*_6_) δ −80.12. IR (KBr): 3437, 2805, 2737, 1631, 1603, 1389, 1350, 1269, 786, 768 cm^–1^. HRMS (ESI) *m/z* calculated for C_21_H_15_F_3_NO_3_ [M + H]^+^ 386.1004, found 386.1017.

#### 3-(1,1,1-Trifluoro-2-hydroxy-3-(isoquinolin-1-yl)propan-2-yl)-2*H*-chromen-2-one (**3an**)

(PE:EA = 5:1, *R*_f_ = 0.35, white solid, mp = 140.8–141.2, 85% yield). ^1^H NMR (400 MHz, Chloroform-*d*) δ 9.27 (s, 1H), 8.56 (s, 1H), 8.40 (d, *J* = 7.0 Hz, 1H), 8.23 (d, *J* = 5.8 Hz, 1H), 7.79–7.76 (m, 1H), 7.73–7.66 (m, 2H), 7.53 (t, *J* = 6.9 Hz, 2H), 7.48–7.42 (m, 1H), 7.23 (t, *J* = 7.6 Hz, 1H), 7.16 (d, *J* = 8.3 Hz, 1H), 5.14 (d, *J* = 15.7 Hz, 1H), 3.83 (d, *J* = 15.6 Hz, 1H). ^13^C NMR (100 MHz, Chloroform-*d*) δ 159.1, 158.1, 153.9, 145.9, 139.2, 136.6, 132.3, 131.1, 128.7, 128.2, 127.5, 127.3, 125.8, 125.3, 124.70 (q, *J* = 284.5 Hz), 124.5, 120.6, 118.7, 116.2, 76.9 (q, *J* = 29.7 Hz). 31.9. ^19^F NMR (376 MHz, Chloroform-*d*) δ −79.65. IR (KBr): 3421, 2844, 2820, 2732, 1676, 1644, 1605, 1391, 1376, 1350, 760, 742 cm^–1^. HRMS (ESI) *m/z* calculated for C_21_H_15_F_3_NO_3_ [M + H]^+^ 386.1004, found 386.1022.

#### 3-(1,1,1-Trifluoro-2-hydroxy-3-(quinoxalin-2-yl)propan-2-yl)-2*H*-chromen-2-one (**3ao**)

(PE:DCM = 1:2, *R*_f_ = 0.22, white solid, mp = 178.9–179.7 °C, 63% yield). ^1^H NMR (400 MHz, Chloroform-*d*) δ 8.95 (s, 1H), 8.45 (s, 1H), 8.07 (s, 1H), 7.92 (s, 1H), 7.75–7.70 (m, 2H), 7.54–7.48 (m, 2H), 7.40 (s, 1H), 7.24 (d, *J* = 8.3 Hz, 3H), 4.55 (d, *J* = 14.8 Hz, 1H), 3.77 (d, *J* = 14.9 Hz, 1H). ^13^C NMR (100 MHz, Chloroform-*d*) δ 159.6, 153.9, 152.4, 146.9, 146.4, 141.9, 140.4, 132.8, 130.6, 130.0, 129.6, 128.7, 128.3, 124.7, 124.4 (q, *J* = 285.6 Hz), 123.3, 118.4, 116.4, 76.8 (q, *J* = 27.3 Hz), 36.1. ^19^F NMR (376 MHz, Chloroform-*d*) δ −80.26. IR (KBr): 3426, 2839, 2805, 2735, 1733, 1634, 1584, 1433, 1386, 1350, 1266, 1159, 1057, 1013, 765, 744, 705 cm^–1^. HRMS (ESI) *m/z* calculated for C_20_H_14_F_3_N_2_O_3_ [M + H]^+^ 387.0957, found 387.0974.

#### 2-(3,3,3-Trifluoro-2-hydroxy-2-(2-oxo-2*H*-chromen-3-yl)propyl)quinazolin-4(3*H*)-one (**3ap**)

(PE:DCM = 1:2, *R*_f_ = 0.22, white solid, mp = 190.1–190.9 °C, 79% yield). ^1^H NMR (400 MHz, Chloroform-*d*) δ 8.47 (s, 1H), 8.33 (s, 1H), 8.10 (d, *J* = 8.4 Hz, 1H), 7.79–7.73 (m, 1H), 7.58–7.43 (m, 4H), 7.29 (dd, *J* = 8.6, 2.6 Hz, 1H), 7.27–7.18 (m, 3H), 4.55 (d, *J* = 14.7 Hz, 1H), 3.72 (d, *J* = 14.7 Hz, 1H). ^13^C NMR (100 MHz, DMSO-*d*_6_) δ 159.7, 156.9, 151.6, 151.0, 146.0, 141.5, 132.8, 130.9, 127.4, 124.7, 124.0, 123.2 (q, *J* = 287.0 Hz), 123.1, 123.0, 119.3, 116.7, 116.4, 114.1, 73.2 (q, *J* = 29.0 Hz), 34.1. ^19^F NMR (376 MHz, DMSO-*d*_6_) δ −80.91. IR (KBr): 3442, 2846, 2815, 2724, 1636, 1592, 1389, 1355, 1266, 789, 768 cm^–1^. HRMS (ESI) *m/z* calculated for C_20_H_14_F_3_N_2_O_4_ [M + H]^+^ 403.0906, found 403.0928.

#### 3-(3-(Benzo[*d*]thiazol-2-yl)-1,1,1-trifluoro-2-hydroxypropan-2-yl)-2*H*-chromen-2-one (**3aq**)

(PE:EA = 3:1, *R*_f_ = 0.32, yellow solid, mp = 141.5–142.8 °C, 41% yield). ^1^H NMR (400 MHz, Chloroform-*d*) δ 8.34 (s, 1H), 7.91 (d, *J* = 7.3 Hz, 1H), 7.81 (d, *J* = 8.1 Hz, 1H), 7.53 (t, *J* = 7.6 Hz, 2H), 7.45–7.40 (m, 1H), 7.37–7.33 (m, 1H), 7.28 (d, *J* = 7.6 Hz, 4H), 7.16 (s, 1H), 4.60 (d, *J* = 15.3 Hz, 1H), 3.85 (d, *J* = 15.3 Hz, 1H). ^13^C NMR (100 MHz, Chloroform-*d*) δ 165.9, 160.2, 153.8, 152.0, 146.1, 134.8, 132.9, 128.9, 127.3 (q, *J* = 285.0 Hz), 126.2, 125.4, 124.9, 122.5, 122.3, 121.7, 118.4, 116.4, 76.4 (q, *J* = 30.7 Hz), 35.9. ^19^F NMR (376 MHz, Chloroform-*d*) δ −80.21. IR (KBr): 3442, 2846, 2807, 1632, 1587, 1435, 1388, 1350, 1164, 782, 759, 730 cm^–1^. HRMS (ESI) *m/z* calculated for C_19_H_13_F_3_NO_3_S [M + H]^+^ 392.0568, found 392.0571.

#### 3-(1,1,1-Trifluoro-2-hydroxy-3-(pyridin-2-yl)propan-2-yl)-2*H*-chromen-2-one (**3ar**)

(PE:EA = 5:1, *R*_f_ = 0.42, white solid, mp = 156.1–157.7 °C, 44% yield). ^1^H NMR (400 MHz, Chloroform-*d*) δ 8.43 (s, 1H), 8.36 (d, *J* = 5.0 Hz, 1H), 7.64 (t, *J* = 7.7 Hz, 1H), 7.51 (t, *J* = 8.2 Hz, 2H), 7.36 (d, *J* = 7.8 Hz, 1H), 7.27 (s, 3H), 7.25 (d, *J* = 7.6 Hz, 2H), 7.21–7.10 (m, 1H), 4.35 (d, *J* = 14.6 Hz, 1H), 3.57 (d, *J* = 14.5 Hz, 1H). ^13^C NMR (100 MHz, Chloroform-*d*) δ 159.1, 157.0, 154.0, 147.4, 146.0, 138.0, 132.4, 128.7, 125.4, 124.7, 124.5 (q, *J* = 286.4 Hz), 124.4, 122.5, 118.6, 116.2, 76.7 (q, *J* = 30.0 Hz), 36.9. ^19^F NMR (376 MHz, Chloroform-*d*) δ −79.84. IR (KBr): 3441, 2849, 2810, 2734, 1633, 1584, 1383, 1349, 796, 765 cm^–1^. HRMS (ESI) *m/z* calculated for C_17_H_13_F_3_NO_3_ [M + H]^+^ 336.0848, found 336.0861.

#### 6-Methyl-3-(1,1,1-trifluoro-2-hydroxy-3-(quinolin-2-yl)propan-2-yl)-2*H*-chromen-2-one (**3ba**)

(PE:EA = 5:1, *R*_f_ = 0.32, white solid, mp = 136.4–137.9 °C, 85% yield). ^1^H NMR (400 MHz, Chloroform-*d*) δ 8.64 (s, 1H), 8.43 (s, 1H), 8.11 (d, *J* = 8.4 Hz, 1H), 7.94 (d, *J* = 8.4 Hz, 1H), 7.77 (d, *J* = 8.1 Hz, 1H), 7.68 (t, *J* = 7.7 Hz, 1H), 7.53–7.46 (m, 2H), 7.27 (d, *J* = 6.9 Hz, 3H), 7.11 (d, *J* = 8.9 Hz, 1H), 4.59 (d, *J* = 14.6 Hz, 1H), 3.73 (d, *J* = 14.6 Hz, 1H), 2.34 (s, 3H). ^13^C NMR (100 MHz, Chloroform-*d*) δ 159.5, 158.3, 152.1, 146.1, 137.8, 134.2, 133.4, 130.1, 128.4, 128.2, 127.8, 127.1, 126.7, 124.6 (q, *J* = 286.0 Hz), 124.5, 123.1, 120.3, 118.4, 115.9, 76.7 (q, *J* = 29.9 Hz), 37.4, 20.7. ^19^F NMR (376 MHz, Chloroform-*d*) δ −80.01. IR (KBr): 3439, 2842, 2808, 2735, 1631, 1603, 1592, 1389, 1350, 1266, 1190, 797, 744, 705 cm^–1^. HRMS (ESI) *m/z* calculated for C_22_H_17_F_3_NO_3_ [M + H]^+^ 400.1161, found 400.1120.

#### 6-Fluoro-3-(1,1,1-trifluoro-2-hydroxy-3-(quinolin-2-yl)propan-2-yl)-2*H*-chromen-2-one (**3ca**)

(PE:EA = 5:1, *R*_f_ = 0.29, white solid, mp = 155.1–156.3 °C, 93% yield). ^1^H NMR (400 MHz, Chloroform-*d*) δ 8.69 (s, 1H), 8.42 (s, 1H), 8.11 (d, *J* = 8.4 Hz, 1H), 7.92 (d, *J* = 8.4 Hz, 1H), 7.77 (d, *J* = 8.1 Hz, 1H), 7.71–7.66 (m, 1H), 7.50 (t, *J* = 7.0 Hz, 1H), 7.46 (d, *J* = 8.4 Hz, 1H), 7.21–7.13 (m, 3H), 4.54 (d, *J* = 14.7 Hz, 1H), 3.73 (d, *J* = 14.7 Hz, 1H). ^13^C NMR (100 MHz, Chloroform-*d*) δ 159.9, 158.1, 150.1, 146.1, 145.1, 137.8, 130.2, 128.1, 127.8, 127.3 (q, *J* = 285.8 Hz), 127.1, 126.7, 126.2, 119.9, 119.7, 119.2, 117.8, 113.9, 113.6, 76.8 (q, *J* = 30.1 Hz), 37.3. ^19^F NMR (376 MHz, Chloroform-*d*) δ −79.85, −117.27. IR (KBr): 3431, 2810, 2735, 2657, 1634, 1605, 1389, 1350, 1193, 1170, 765, 741 cm^–1^. HRMS (ESI) *m/z* calculated for C_21_H_13_F_4_NO_3_ [M + H]^+^ 404.0910, found 404.0922.

#### 6-Chloro-3-(1,1,1-trifluoro-2-hydroxy-3-(quinolin-2-yl)propan-2-yl)-2*H*-chromen-2-one (**3da**)

(PE:EA = 5:1, *R*_f_ = 0.28, white solid, mp = 168.4–169.8 °C, 95% yield). ^1^H NMR (400 MHz, Chloroform-*d*) δ 8.71 (s, 1H), 8.40 (s, 1H), 8.11 (d, *J* = 8.4 Hz, 1H), 7.92 (d, *J* = 8.5 Hz, 1H), 7.76 (d, *J* = 8.2 Hz, 1H), 7.68 (t, *J* = 7.7 Hz, 1H), 7.50 (t, *J* = 6.9 Hz, 1H), 7.46–7.43 (m, 2H), 7.39 (dd, *J* = 8.8, 2.5 Hz, 1H), 7.14 (d, *J* = 8.8 Hz, 1H), 4.54 (d, *J* = 14.9 Hz, 1H), 3.72 (d, *J* = 14.8 Hz, 1H). ^13^C NMR (100 MHz, Chloroform-*d*) δ 158.5, 158.1, 152.3, 146.1, 144.9, 137.9, 132.2, 130.2, 129.7, 128.1, 127.8, 127.1, 126.7, 126.3, 125.8, 124.4 (q, *J* = 284.0 Hz), 123.0, 119.6, 117.6, 76.8 (q, *J* = 30.3 Hz), 37.2. ^19^F NMR (376 MHz, Chloroform-*d*) δ −79.81. IR (KBr): 3433, 2846, 2721, 2700, 1670, 1639, 1594, 1373, 1346, 783, 767 cm^–1^. HRMS (ESI) *m/z* calculated for C_21_H_14_ClF_3_NO_3_ [M + H]^+^ 420.0614, found 420.0636.

#### 6-Bromo-3-(1,1,1-trifluoro-2-hydroxy-3-(quinolin-2-yl)propan-2-yl)-2*H*-chromen-2-one (**3ea**)

(PE:EA = 5:1, *R*_f_ = 0.25, white solid, mp = 177.3–178.6 °C, 77% yield). ^1^H NMR (400 MHz, Chloroform-*d*) δ 8.70 (s, 1H), 8.40 (s, 1H), 8.11 (d, *J* = 8.4 Hz, 1H), 7.92 (d, *J* = 8.5 Hz, 1H), 7.76 (d, *J* = 8.1 Hz, 1H), 7.69 (t, *J* = 7.7 Hz, 1H), 7.60 (d, *J* = 2.3 Hz, 1H), 7.54–7.48 (m, 2H), 7.45 (d, *J* = 8.4 Hz, 1H), 7.08 (d, *J* = 8.8 Hz, 1H), 4.54 (d, *J* = 14.7 Hz, 1H), 3.72 (d, *J* = 14.7 Hz, 1H). ^13^C NMR (100 MHz, Chloroform-*d*) δ 158.5, 158.1, 152.8, 146.1, 144.9, 137.9, 135.1, 130.9, 130.3, 128.1, 127.8, 127.7 (q, *J* = 286.0 Hz), 127.1, 126.8, 126.3, 123.0, 120.1, 117.9, 117.0, 76.8 (q, *J* = 29.9 Hz), 36.3. ^19^F NMR (376 MHz, Chloroform-*d*) δ −79.87. IR (KBr): 3446, 2844, 2820, 2802, 2726, 2703, 1675, 1641, 1602, 1391, 1373, 1344, 1049, 791, 767 cm^–1^. HRMS (ESI) *m/z* calculated for C_21_H_14_BrF_3_NO_3_ [M + H]^+^ 464.0109, found 464.0133.

#### 6-Iodo-3-(1,1,1-trifluoro-2-hydroxy-3-(quinolin-2-yl)propan-2-yl)-2*H*-chromen-2-one (**3fa**)

(PE:EA = 5:1, *R*_f_ = 0.27, white solid, mp = 173.9–174.4 °C, 67% yield). ^1^H NMR (400 MHz, Chloroform-*d*) δ 8.67 (s, 1H), 8.37 (s, 1H), 8.11 (d, *J* = 8.4 Hz, 1H), 7.92 (d, *J* = 8.5 Hz, 1H), 7.81–7.74 (m, 2H), 7.73–7.65 (m, 2H), 7.50 (t, *J* = 7.5 Hz, 1H), 7.44 (d, *J* = 8.4 Hz, 1H), 6.95 (d, *J* = 8.7 Hz, 1H), 4.53 (d, *J* = 14.7 Hz, 1H), 3.72 (d, *J* = 14.7 Hz, 1H). ^13^C NMR (100 MHz, Chloroform-*d*) δ 158.4, 158.1, 153.5, 146.1, 144.7, 140.7, 137.8, 137.0, 130.2, 128.1, 127.8, 127.1, 126.7, 126.1, 124.4 (q, *J* = 284.0 Hz), 123.0, 120.7, 118.1, 87.7, 76.7 (q, *J* = 30.3 Hz), 37.3. ^19^F NMR (376 MHz, Chloroform-*d*) δ −79.83. IR (KBr): 3439, 2847, 2813, 2740, 1634, 1600, 1386, 1350, 1266, 786, 768 cm^–1^. HRMS (ESI) *m/z* calculated for C_21_H_14_F_3_INO_3_ [M + H]^+^ 511.9970, found 511.9993.

#### 7-Methoxy-3-(1,1,1-trifluoro-2-hydroxy-3-(quinolin-2-yl)propan-2-yl)-2*H*-chromen-2-one (**3ga**)

(PE:EA = 5:1, *R*_f_ = 0.37, white solid, mp = 84.8–85.4 °C, 86% yield). ^1^H NMR (400 MHz, Chloroform-*d*) δ 8.56 (s, 1H), 8.39 (s, 1H), 8.10 (d, *J* = 8.4 Hz, 1H), 7.92 (d, *J* = 8.3 Hz, 1H), 7.76 (d, *J* = 9.6 Hz, 1H), 7.67 (t, 1H), 7.50 (d, *J* = 7.9 Hz, 2H), 7.36 (d, *J* = 8.7 Hz, 1H), 6.77 (dd, *J* = 8.7, 2.4 Hz, 1H), 6.68 (d, *J* = 2.4 Hz, 1H), 4.55 (d, *J* = 14.6 Hz, 1H), 3.80 (s, 3H), 3.70 (d, *J* = 14.6 Hz, 1H). ^13^C NMR (100 MHz, Chloroform-*d*) δ 163.4, 159.6, 158.4, 155.9, 146.2, 146.1, 137.7, 130.1, 129.7, 128.2, 127.8, 127.1, 126.6, 124.7 (q, *J* = 285.7 Hz), 123.2, 120.8, 112.9, 112.4, 100.0, 76.6 (q, *J* = 29.4 Hz), 55.8, 37.4. ^19^F NMR (376 MHz, Chloroform-*d*) δ −80.20. IR (KBr): 3439, 2855, 2800, 2659, 1633, 1616, 1593, 1391, 1372, 1346, 778, 736 cm^–1^. HRMS (ESI) *m/z* calculated for C_22_H_17_F_3_NO_4_ [M + H]^+^ 416.1110, found 416.1129.

#### 8-Methoxy-3-(1,1,1-trifluoro-2-hydroxy-3-(quinolin-2-yl)propan-2-yl)-2*H*-chromen-2-one (**3ha**)

(PE:EA = 5:1, *R*_f_ = 0.32, white solid, mp = 176.1–177.2 °C, 88% yield). ^1^H NMR (400 MHz, Chloroform-*d*) δ 8.59 (s, 1H), 8.43 (s, 1H), 8.09 (d, *J* = 8.4 Hz, 1H), 7.92 (d, *J* = 8.5 Hz, 1H), 7.75 (d, *J* = 7.8 Hz, 1H), 7.66 (t, 1H), 7.52–7.44 (m, 2H), 7.12 (t, *J* = 7.9 Hz, 1H), 7.03 (d, *J* = 6.4 Hz, 1H), 6.98 (d, *J* = 8.0 Hz, 1H), 4.59 (d, *J* = 14.6 Hz, 1H), 3.88 (s, 3H), 3.70 (d, *J* = 14.4 Hz, 1H). ^13^C NMR (100 MHz, Chloroform-*d*) δ 158.6, 158.2, 146.7, 146.3, 143.6, 137.8, 130.1, 128.1, 127.8, 127.1, 126.7, 125.0, 124.5 (q, *J* = 284.0 Hz), 124.3, 123.2, 120.3, 119.9, 119.2, 113.9, 76.4 (q, *J* = 29.9 Hz), 56.2, 37.3. ^19^F NMR (376 MHz, Chloroform-*d*) δ −79.97. IR (KBr): 3462, 2852, 2821, 1741, 1625, 1617, 1609, 1597, 1586, 1579, 1369, 1360, 1281, 1106, 945, 830, 734 cm^–1^. HRMS (ESI) *m/z* calculated for C_22_H_17_F_3_NO_4_ [M + H]^+^ 416.1110, found 416.1090.

#### 8-Ethoxy-3-(1,1,1-trifluoro-2-hydroxy-3-(quinolin-2-yl)propan-2-yl)-2*H*-chromen-2-one (**3ia**)

(PE:EA = 5:1, *R*_f_ = 0.42, white solid, mp = 147.2–147.9 °C, 83% yield). ^1^H NMR (400 MHz, Chloroform-*d*) δ 8.59 (s, 1H), 8.43 (s, 1H), 8.09 (d, *J* = 8.4 Hz, 1H), 7.91 (d, *J* = 8.5 Hz, 1H), 7.75 (d, *J* = 9.5 Hz, 1H), 7.69–7.64 (m, 1H), 7.48 (t, *J* = 9.0 Hz, 2H), 7.10 (t, *J* = 7.9 Hz, 1H), 7.03 (d, *J* = 7.9 Hz, 1H), 6.98 (d, *J* = 8.0 Hz, 1H), 4.60 (d, *J* = 14.7 Hz, 1H), 4.09 (q, *J* = 7.0 Hz, 2H), 3.70 (d, *J* = 14.6 Hz, 1H), 1.44 (t, *J* = 7.0 Hz, 3H). ^13^C NMR (100 MHz, Chloroform-*d*) δ 158.8, 158.3, 146.3, 146.2, 146.1, 143.8, 137.7, 130.1, 128.1, 127.8, 127.1, 126.6, 124.9, 124.5 (q, *J* = 284.0 Hz), 124.3, 123.2, 119.9, 119.4, 115.1, 76.7 (q, *J* = 29.9 Hz), 64.9, 37.4, 14.7. ^19^F NMR (376 MHz, Chloroform-d) δ −80.05. IR (KBr): 3454, 2852, 2807, 2732, 1657, 1597, 1383, 1352, 1269, 786, 763 cm^–1^. HRMS (ESI) *m/z* calculated for C_23_H_18_F_3_NO_4_[M + H]^+^ 430.1266, found 430.1262.

#### 6,8-Dichloro-3-(1,1,1-trifluoro-2-hydroxy-3-(quinolin-2-yl)propan-2-yl)-2*H*-chromen-2-one (**3ja**)

(PE:EA = 5:1, *R*_f_ = 0.32, white solid, mp = 195.1–196.1 °C, 84% yield). ^1^H NMR (400 MHz, Chloroform-*d*) δ 8.74 (s, 1H), 8.39 (s, 1H), 8.13 (d, *J* = 8.3 Hz, 1H), 7.92 (d, *J* = 8.5 Hz, 1H), 7.78 (d, *J* = 8.2 Hz, 1H), 7.69 (t, *J* = 7.7 Hz, 1H), 7.52 (d, *J* = 7.5 Hz, 1H), 7.49 (d, *J* = 2.3 Hz, 1H), 7.45 (d, *J* = 8.4 Hz, 1H), 7.37 (s, 1H), 4.53 (d, *J* = 14.7 Hz, 1H), 3.72 (d, *J* = 15.0 Hz, 1H). ^13^C NMR (100 MHz, Chloroform-*d*) δ 158.0, 157.5, 148.4, 146.1, 144.8, 138.1, 132.2, 130.3, 129.6, 128.1, 127.9, 127.4, 127.2, 126.9, 126.4, 125.7, 124.3 (q, *J* = 285.8 Hz), 123.0, 122.9, 122.1, 76.5 (q, *J* = 29.9 Hz), 37.9. ^19^F NMR (376 MHz, Chloroform-*d*) δ −79.86. IR (KBr): 3442, 2842, 2803, 2737, 1673, 1634, 1587, 1394, 1370, 1350, 1263, 1190, 780, 746 cm^–1^. HRMS (ESI) *m/z* calculated for C_21_H_13_Cl_2_F_3_NO_3_ [M + H]^+^ 454.0237, found 454.0237.

#### 6,8-Dibromo-3-(1,1,1-trifluoro-2-hydroxy-3-(quinolin-2-yl)propan-2-yl)-2H-chromen-2-one (**3ka**)

(PE:EA = 5:1, *R*_f_ = 0.43, white solid, mp = 189.1–189.6 °C, 89% yield). ^1^H NMR (400 MHz, Chloroform-*d*) δ 8.28 (s, 1H), 8.05 (d, *J* = 8.4 Hz, 1H), 7.83 (d, *J* = 8.5 Hz, 1H), 7.72–7.66 (m, 2H), 7.63–7.58 (m, 1H), 7.47–7.40 (m, 2H), 7.36 (d, *J* = 8.4 Hz, 1H), 4.45 (d, *J* = 14.8 Hz, 1H), 3.64 (d, *J* = 14.7 Hz, 1H). ^13^C NMR (100 MHz, Chloroform-*d*) δ 157.9, 157.4, 149.8, 146.0, 144.6, 138.0, 137.6, 130.3, 130.1, 128.0, 127.8, 127.3, 127.1, 126.8, 124.3 (q, *J* = 285.8 Hz), 123.0, 120.8, 116.9, 110.6, 76.8 (q, *J* = 29.9 Hz), 37.1. ^19^F NMR (376 MHz, Chloroform-*d*) δ −79.80. IR (KBr): 3431, 2844, 2805, 2732, 1736, 1673, 1634, 1590, 1389, 1347, 1269, 1193, 1154, 783, 763, 737 cm^–1^. HRMS (ESI) *m/z* calculated for C_21_H_13_Br_2_F_3_NO_3_ [M + H]^+^ 543.9194, found 543.9181.

#### 2-(1,1,1-Trifluoro-2-hydroxy-3-(quinolin-2-yl)propan-2-yl)-3*H*-benzo[*f*]chromen-3-one (**3la**)

(PE:DCM = 1:1, *R*_f_ = 0.33, white solid, mp = 146.9–147.3 °C, 91% yield). ^1^H NMR (400 MHz, Chloroform-*d*) δ 9.26 (s, 1H), 8.71 (s, 1H), 8.35 (d, *J* = 8.4 Hz, 1H), 8.11 (d, *J* = 8.4 Hz, 1H), 7.92 (d, *J* = 9.2 Hz, 2H), 7.84 (d, *J* = 8.2 Hz, 1H), 7.74 (d, *J* = 7.9 Hz, 1H), 7.69 (t, 1H), 7.63 (t, 1H), 7.56–7.50 (m, 2H), 7.46 (t, *J* = 7.5 Hz, 1H), 7.34 (d, *J* = 9.0 Hz, 1H), 4.62 (d, *J* = 14.6 Hz, 1H), 3.78 (d, *J* = 14.7 Hz, 1H). ^13^C NMR (100 MHz, Chloroform-*d*) δ 159.4, 158.3, 153.9, 146.2, 141.8, 137.7, 133.8, 130.2, 129.2, 128.9, 128.4, 128.2, 127.8, 127.1, 126.6, 126.1, 125.4 (q, *J* = 283.1 Hz), 123.4, 123.3, 123.1, 121.8, 116.3, 112.9, 76.9 (q, *J* = 26.3 Hz), 37.4. ^19^F NMR (376 MHz, Chloroform-*d*) δ −79.96. IR (KBr): 3420, 2843, 2805, 2732, 1673, 1644, 1584, 1389, 1376, 1347, 1268, 794, 768, 739 cm^–1^. HRMS (ESI) *m/z* calculated for C_25_H_16_F_3_NO_3_ [M + H]^+^ 436.1161, found 436.1171.

#### 1,1,1-Trifluoro-2-phenyl-3-(quinolin-2-yl)propan-2-ol (**3ma**)

(PE:EA = 3:1, *R*_f_ = 0.45, white solid, mp = 97.9–98.8 °C, 52% yield). ^1^H NMR (400 MHz, Chloroform-*d*) δ 8.52 (s, 1H), 8.07 (d, *J* = 8.3 Hz, 1H), 7.98 (d, *J* = 8.5 Hz, 1H), 7.76 (d, *J* = 8.2 Hz, 1H), 7.70 (d, *J* = 7.9 Hz, 3H), 7.51 (t, *J* = 8.1 Hz, 1H), 7.31 (t, *J* = 7.4 Hz, 2H), 7.25 (dd, *J* = 7.8, 4.7 Hz, 3H), 3.80 (d, *J* = 15.1 Hz, 1H), 3.69 (d, *J* = 15.1 Hz, 1H). ^13^C NMR (100 MHz, Chloroform-*d*) δ 157.8, 146.3, 138.5, 137.6, 130.2, 129.5, 128.4, 128.2, 128.1, 127.7, 126.8, 126.7, 126.6, 125.3 (q, *J* = 282.8 Hz), 123.8, 122.5, 77.4 (q, *J* = 29.7 Hz), 40.2. ^19^F NMR (376 MHz, Chloroform-*d*) δ −79.28. IR (KBr): 3428, 2810, 2732, 2658, 1654, 1633, 1592, 1386, 1349, 1266, 1180, 796, 741, 700 cm^–1^. HRMS (ESI) *m/z* calculated for C_18_H_14_F_3_NO [M + H]^+^ 318.1106, found 318.1114.

### General Procedure for the Evaluation of Fungicidal Activities

16.6 mg of the test sample was taken and dissolved in 0.66 mL of DMSO, and then, an aqueous solution containing 1% Tween 80 was added to it to make 5 mg/mL of the original drug. An appropriate amount of the test drug was pipetted into a conical flask under aseptic conditions and shaken well, and then the same amount was poured into three petri dishes with a diameter of 9 cm to make a 500 μg/mL drug-containing plate. In the abovementioned experiments, the treatment without the drug was set as a blank control, and each treatment was repeated three times. The cultured pathogenic bacteria were cut along the edge of the colony with a hole punch with a diameter of 6.5 mm under aseptic conditions, and the bacterial cake was inoculated in the center of the drug-containing plate with an inoculator. The culture dish was cultured in a constant temperature incubator at 25 °C. When the diameter of the control colony expanded to more than 6 cm, the colony diameter was measured by the cross method, the average value was taken, and the bacteriostatic rate was calculated at the end of the culture.

## Supporting Information Available

^1^H NMR, ^19^F NMR, ^13^C NMR, and HRMS spectra for all new compounds (PDF)
