# Supplementary material for: Highly Chemoselective Synthesis of Azaarene-Equipped CF3-Tertiary Alcohols under Metal-Free Conditions and Their Fungicidal Activities
Source: ACS Omega. 2022 Oct 10;7(42):38084–93. doi: 10.1021/acsomega.2c05855 (PMC9609063; doi:10.1021/acsomega.2c05855)

Highly Chemoselective Synthesis of Azaarene-Equipped CF_3-Tertiary Alcohols under Metal-Free Conditions and Their Fungicidal Activities

Bingyi Zhou, [a, b] Guoyu Yang, [a] Caixia Wang, [a] Lijie Liu, [a] Lijun Shi, [a] Zhenliang Pan, [a] Xiaoming Ji, [b, *] Lulu Wu, [a] Huayu Zheng, [c] Cuilian Xu [a, *] and Liangxin Fan[a, *]

[a] College of Sciences, Henan Agricultural University, Zhengzhou 450002, China

[b] College of Tobacco Sciences, Henan Agricultural University, Zhengzhou 450002, China

[c] College of Sciences, Chang'an University, Xi'an 710064, China

Email: fanlx@henau.edu.cn; xucuilian666@126.com; xiaomingji@henau.edu.cn

Table of Contents

A. Large-scale preparation of **3ka** 2

B. The original data of fungicidal activities 2

C. EC_{50} value of compound **3al** 3

D. NMR spectra 4

E. HRMS spectra 49

A. Large-scale preparation of 3ka

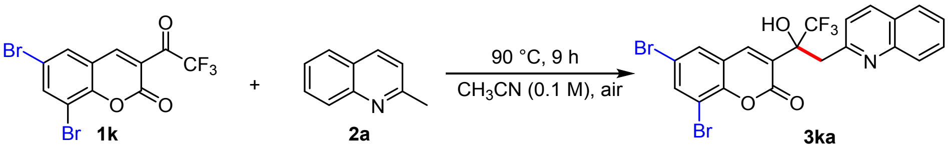

A 100 mL vial equipped with a stirring bar was charged with trifluoromethyl ketone **1k** (3.0 mmol, 1.20 g, 1.0 equiv.), 2-methylquinoline **2a** (6.0 mmol, 0.86 g, 2.0 equiv.) and CH_3CN (30 mL) was added. The vial was sealed with a Teflon screw cap and the reaction mixture was heated at 90 ºC for 9 h under pressure. After the reaction vessel was cooled to room temperature, the crude reaction mixture was filtered with celite and washed with DCM. The solvent was removed under reduced pressure. Then the residue was purified by silica gel column chromatography to afford the desired product **3ka**. (PE:EA = 8:1, R_f = 0.33, 1.43 g, 88% yield).

B. The original data of fungicidal activities

Compound	*F.moniliforme*	*F.graminearum*	*F.oxysporum*	*R.solani*	*P.nicotianae*
**3aa**	32.32	16.09	29.84	52.84	20.21
**3ab**	16.09	24.52	8.67	61.06	11.26
**3ac**	20.54	16.99	14.67	68.76	15.10
**3ad**	22.31	25.75	33.33	70.35	10.54
**3ae**	31.30	20.45	18.22	83.51	27.77
**3af**	18.25	25.73	7.34	59.43	13.12
**3ag**	20.31	21.37	8.86	62.28	12.54
**3ah**	26.21	6.46	13.95	63.14	8.74
**3ai**	40.33	5.41	11.89	59.28	5.08
**3aj**	19.42	17.82	25.76	71.02	10.00
**3ak**	24.26	19.28	12.89	66.70	14.11
**3al**	16.46	29.37	8.00	91.65	10.27
**3am**	19.31	29.25	22.22	66.16	27.97
**3an**	31.55	8.44	11.89	63.92	11.60
**3ao**	41.46	51.09	45.11	88.94	44.86
**3ap**	35.64	41.12	17.56	81.56	14.11
**3aq**	45.54	39.77	44.32	72.02	37.84
**3ar**	16.46	24.78	10.00	72.67	17.45
**3ba**	30.15	9.50	18.60	63.14	14.21
**3ca**	32.95	5.54	18.60	49.23	6.91
**3da**	25.58	27.18	16.34	62.28	16.91
**3ea**	17.35	26.39	10.39	61.79	23.62

**3fa**	18.69	35.76	26.00	51.95	18.32
**3ga**	21.37	13.98	24.42	65.46	13.56
**3ha**	25.45	9.89	11.11	57.73	9.52
**3ia**	2.48	22.61	9.56	65.40	16.09
**3ja**	28.63	4.22	17.96	47.16	13.95
**3ka**	33.33	12.14	2.45	67.01	9.78
**3la**	12.60	27.44	0.83	66.50	14.58
**3ma**	12.13	16.48	15.33	84.82	12.38
**Triazolone**	85.96	42.98	45.37	85.58	35.97

C. EC_{50} value of compound 3al

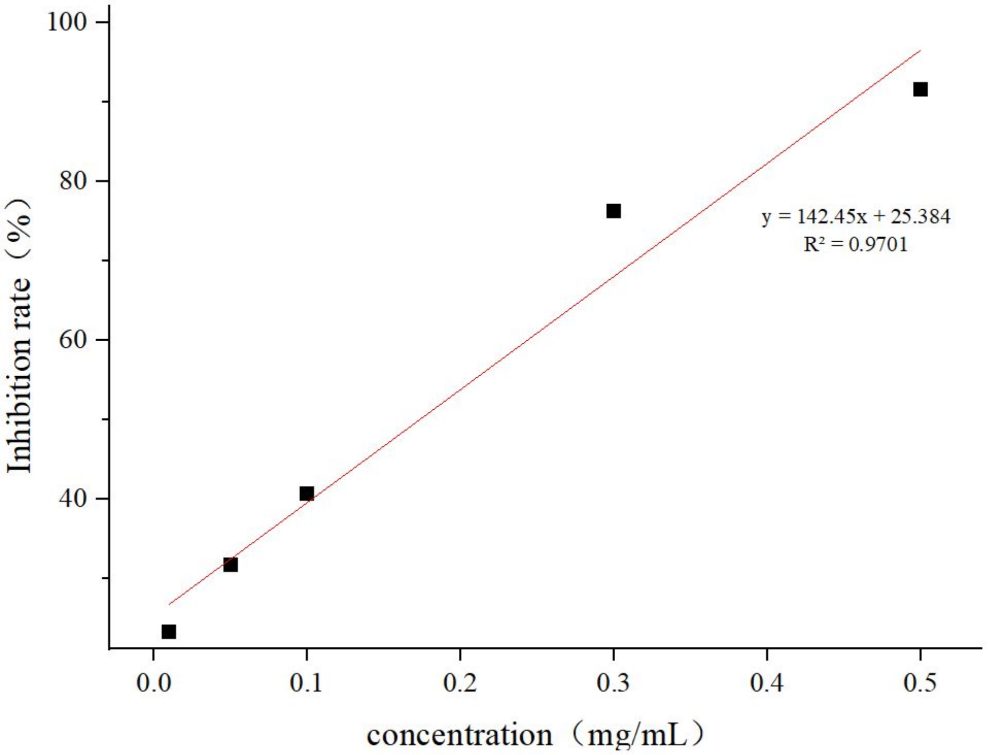

D. NMR spectra

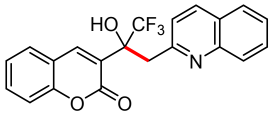

(3aa)

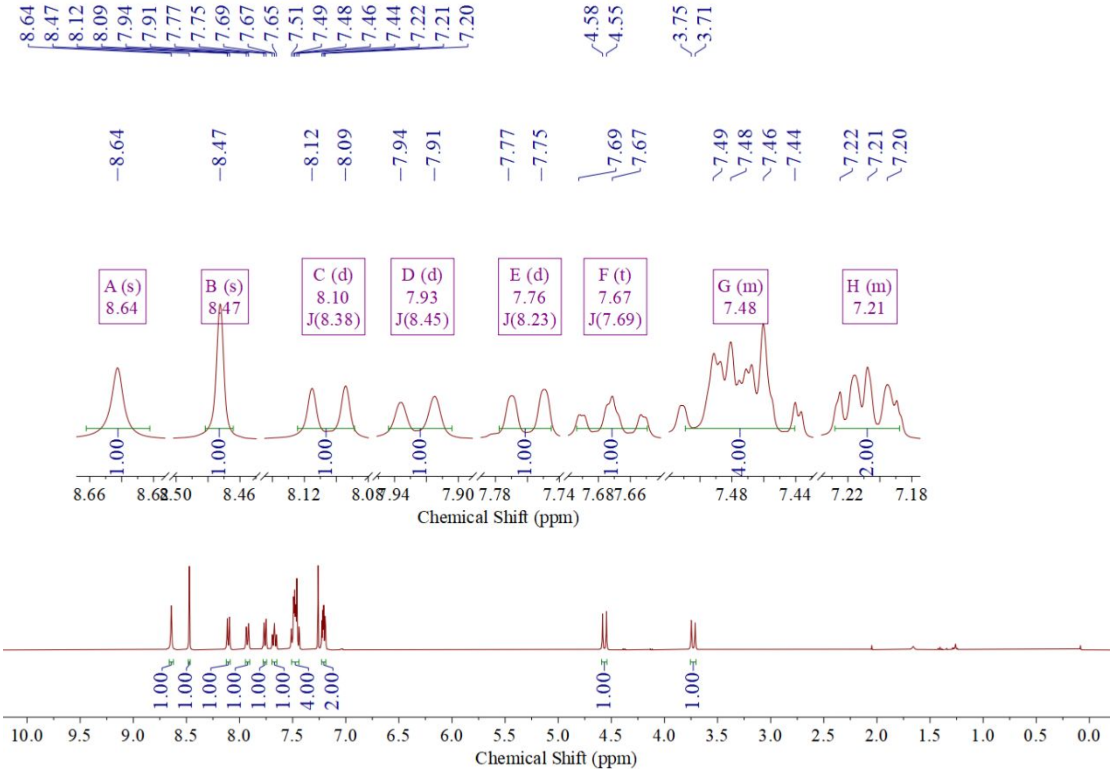

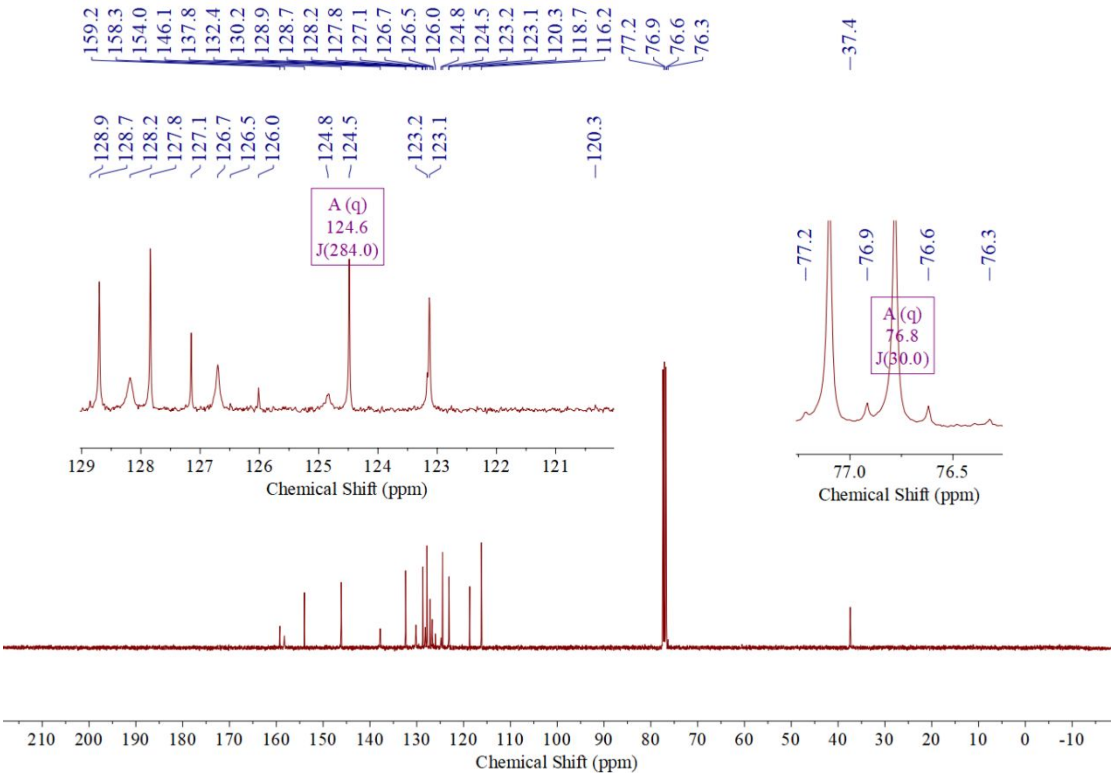

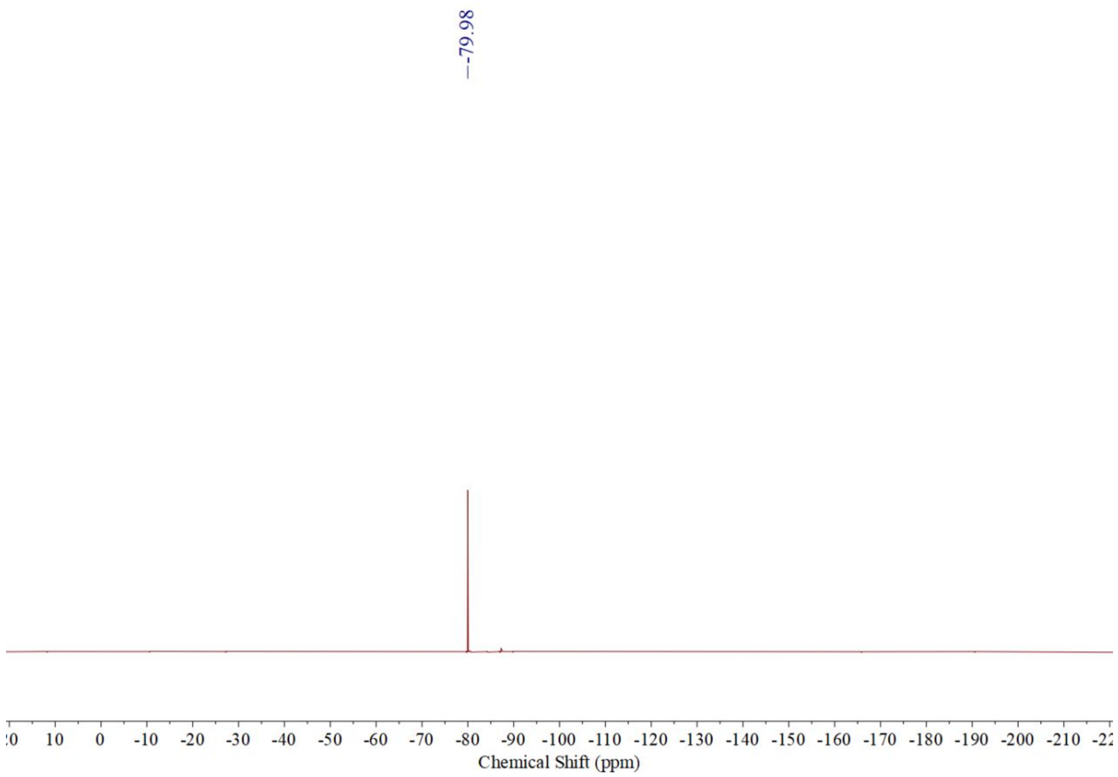
-79.98
Chemical Shift (ppm)

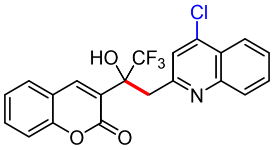
Cl
HO
CF3
N
O
O

(3ab)

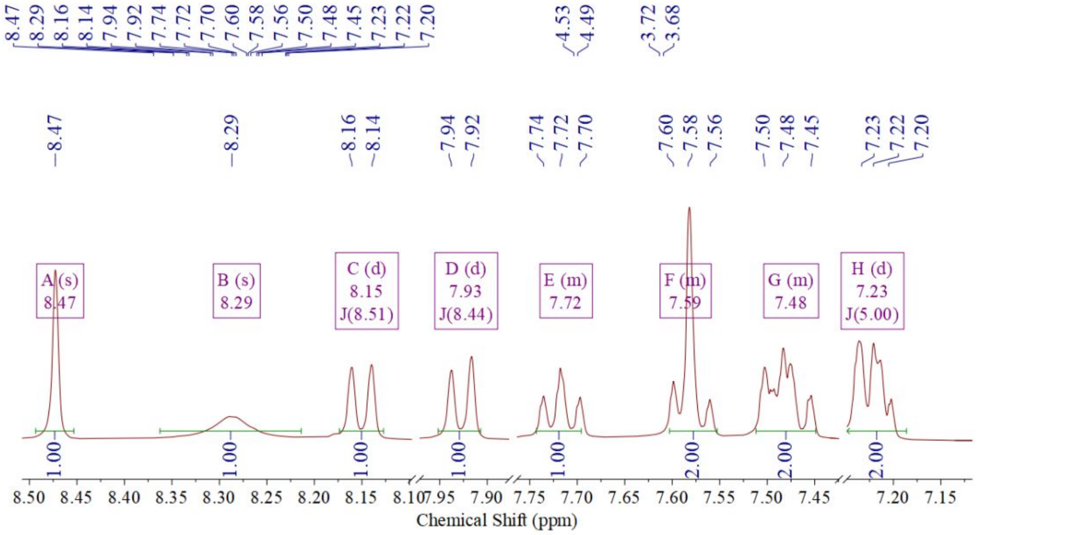
A (s) 8.47
B (s) 8.29
C (d) 8.15 J(8.51)
D (d) 7.93 J(8.44)
E (m) 7.72
F (m) 7.59
G (m) 7.48
H (d) 7.23 J(5.00)
Chemical Shift (ppm)

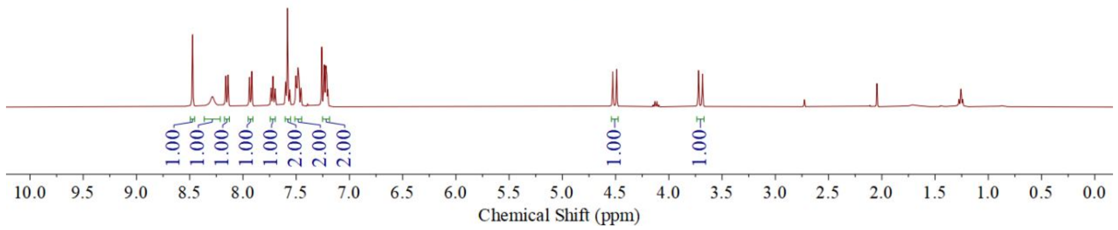
Chemical Shift (ppm)

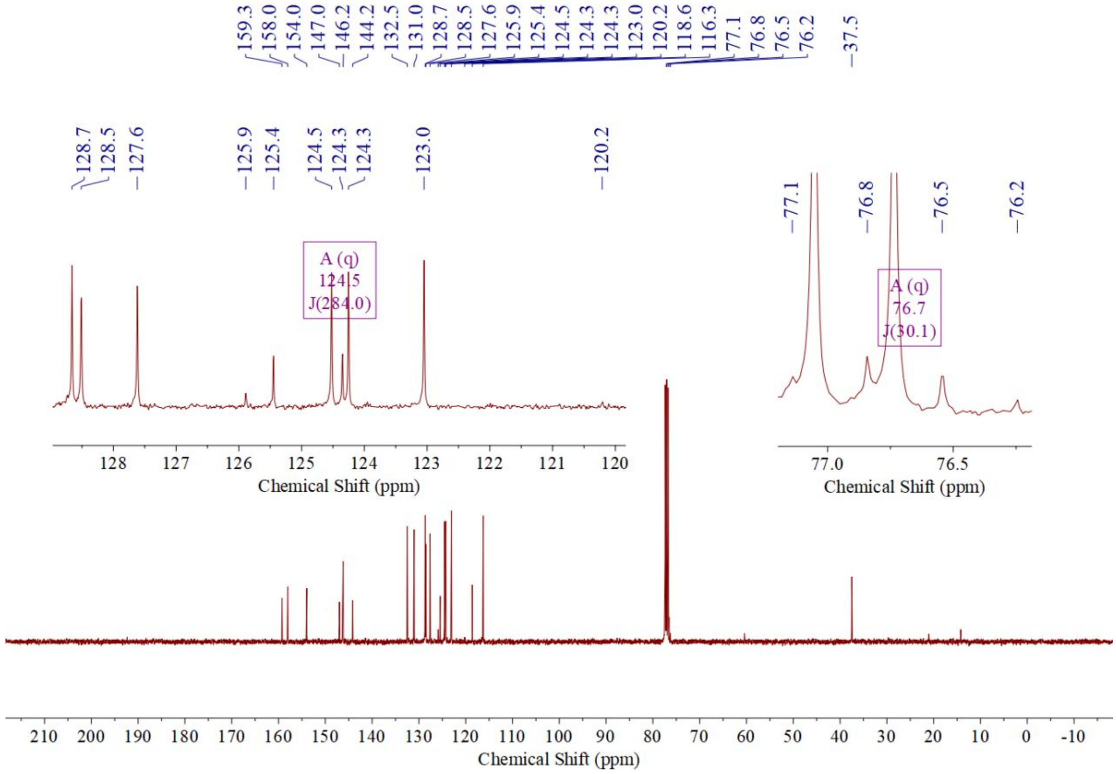
159.3
158.0
154.0
147.0
146.2
144.2
132.5
131.0
128.7
128.5
127.6
125.9
125.4
124.5
124.3
124.3
123.0
120.2
118.6
116.3
77.1
76.8
76.5
76.2
37.5
A (q)
124.5
J(284.0)
A (q)
76.7
J(30.1)
Chemical Shift (ppm)
Chemical Shift (ppm)
Chemical Shift (ppm)

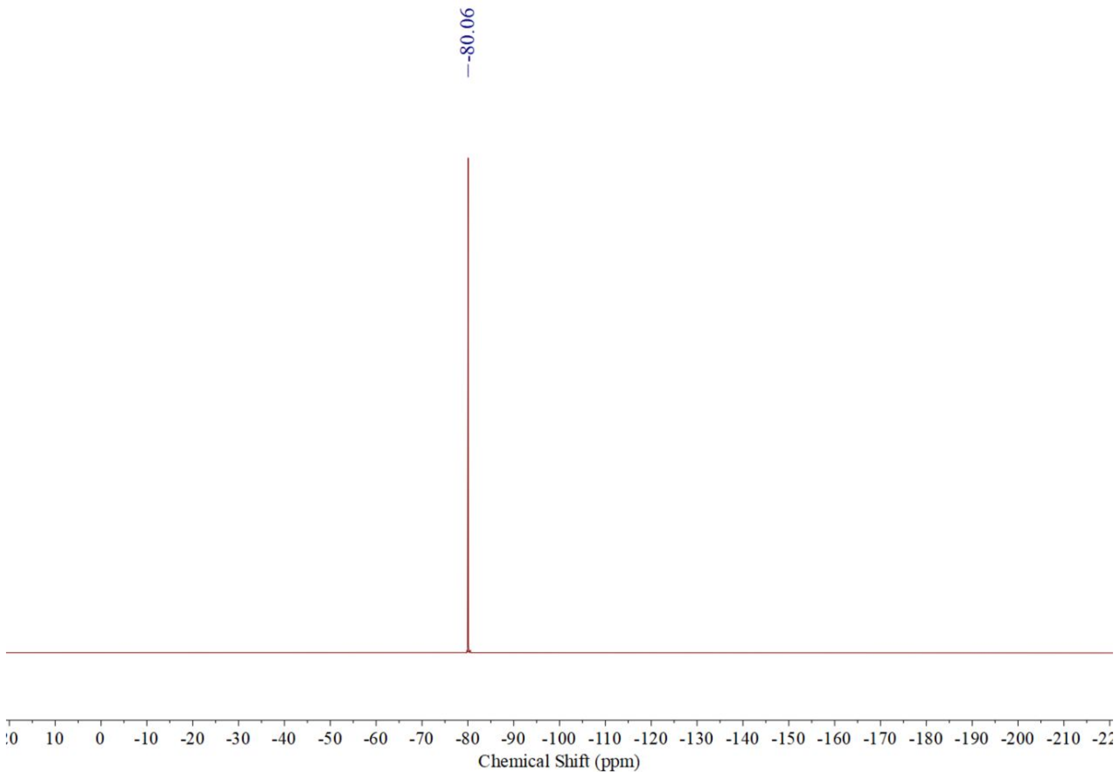
-80.06
Chemical Shift (ppm)

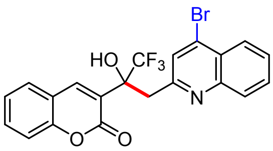
Br
HO
CF3
N
O
O

(3ac)

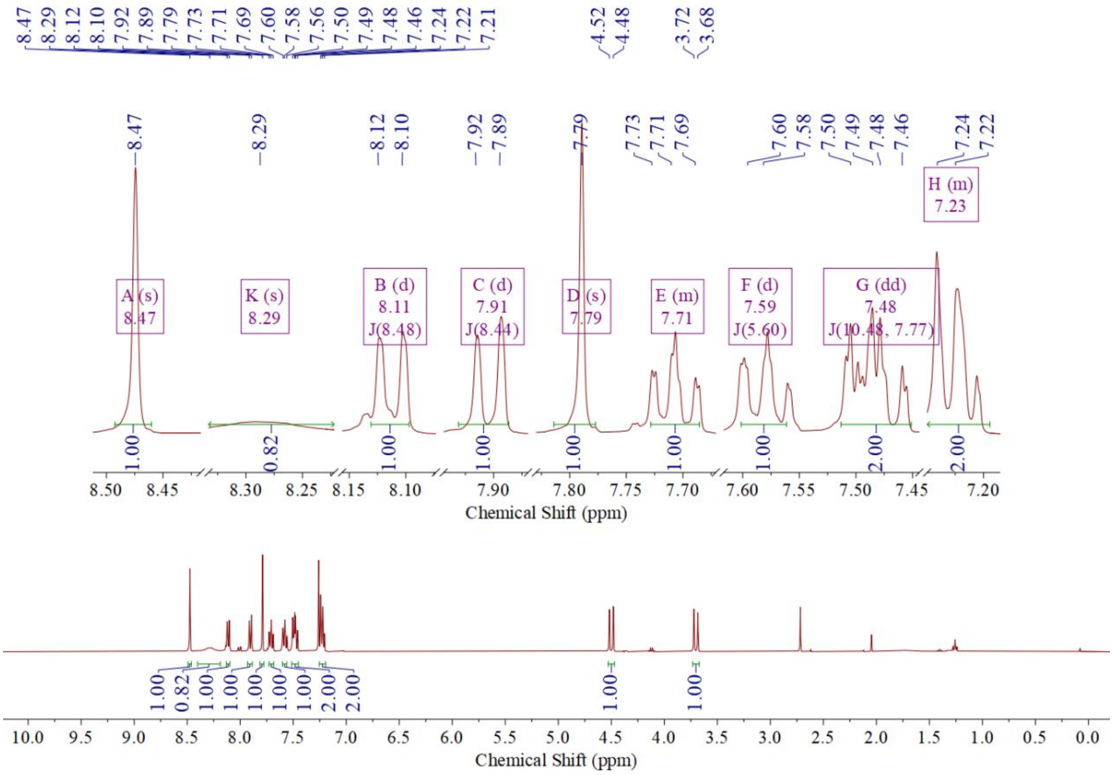
8.47 8.29 8.12 8.10 7.92 7.89 7.79 7.73 7.71 7.69 7.60 7.58 7.56 7.50 7.49 7.48 7.46 7.24 7.22 7.21
4.52 4.48
3.72 3.68
A (s) 8.47
K (s) 8.29
B (d) 8.11 J(8.48)
C (d) 7.91 J(8.44)
D (s) 7.79
E (m) 7.71
F (d) 7.59 J(5.60)
G (dd) 7.48 J(10.48, 7.77)
H (m) 7.23
1.00 0.82 1.00 1.00 1.00 1.00 1.00 2.00 2.00
Chemical Shift (ppm)
1.00 0.82 1.00 1.00 1.00 1.00 1.00 2.00 2.00 1.00 1.00
Chemical Shift (ppm)

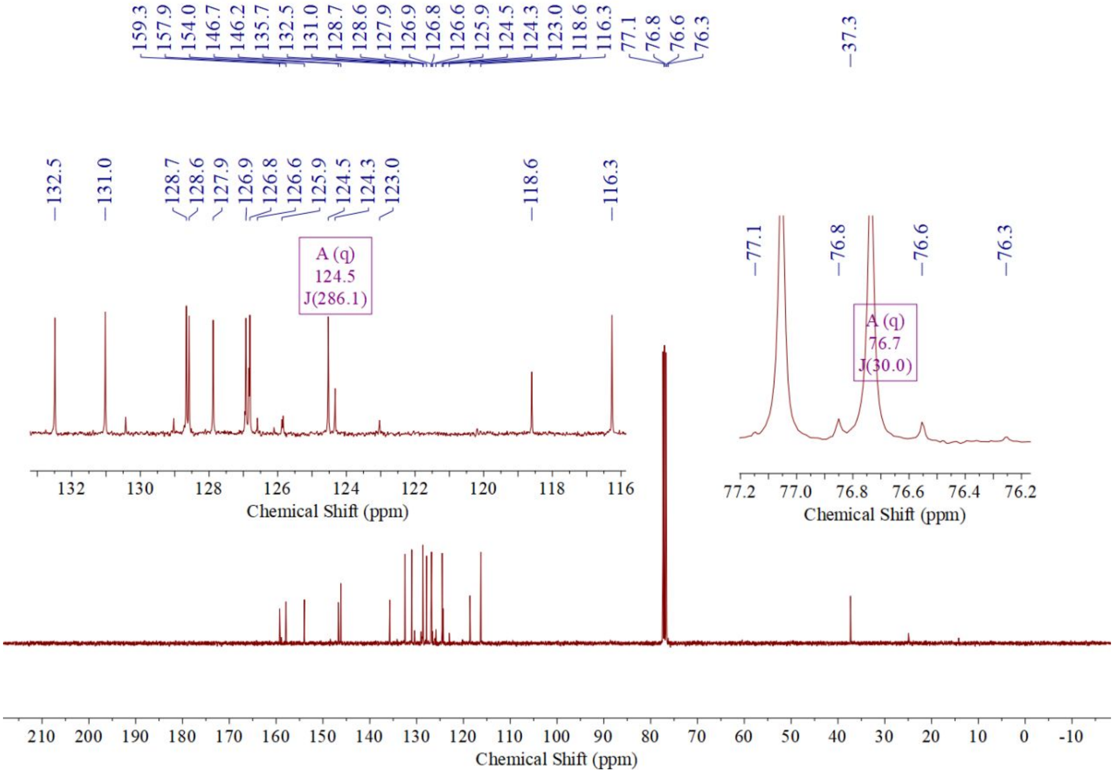
159.3 157.9 154.0 146.7 146.2 135.7 132.5 131.0 128.7 128.6 127.9 126.9 126.8 126.6 125.9 124.5 124.3 123.0 118.6 116.3 77.1 76.8 76.6 76.3
37.3
A (q) 124.5 J(286.1)
Chemical Shift (ppm)
A (q) 76.7 J(30.0)
Chemical Shift (ppm)
Chemical Shift (ppm)

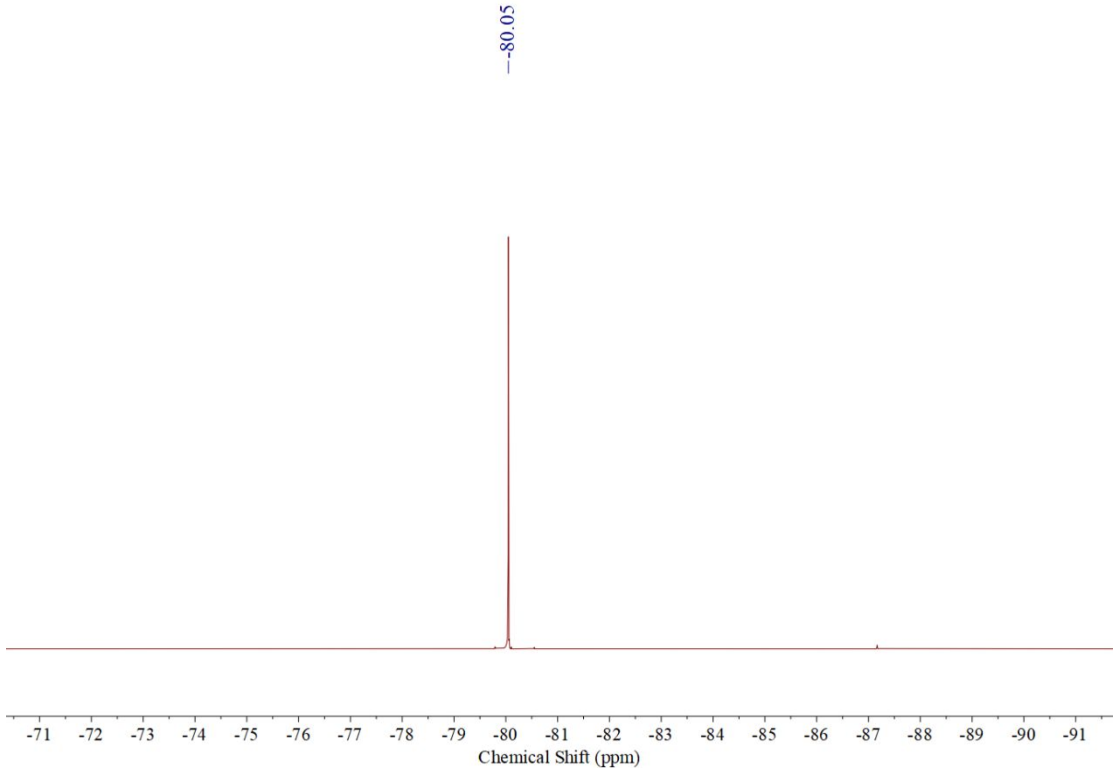
-80.05
-71 -72 -73 -74 -75 -76 -77 -78 -79 -80 -81 -82 -83 -84 -85 -86 -87 -88 -89 -90 -91
Chemical Shift (ppm)

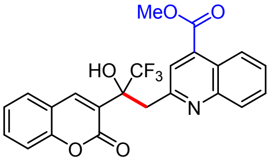
MeO
O
HO
CF3
N
O
O

(3ad)

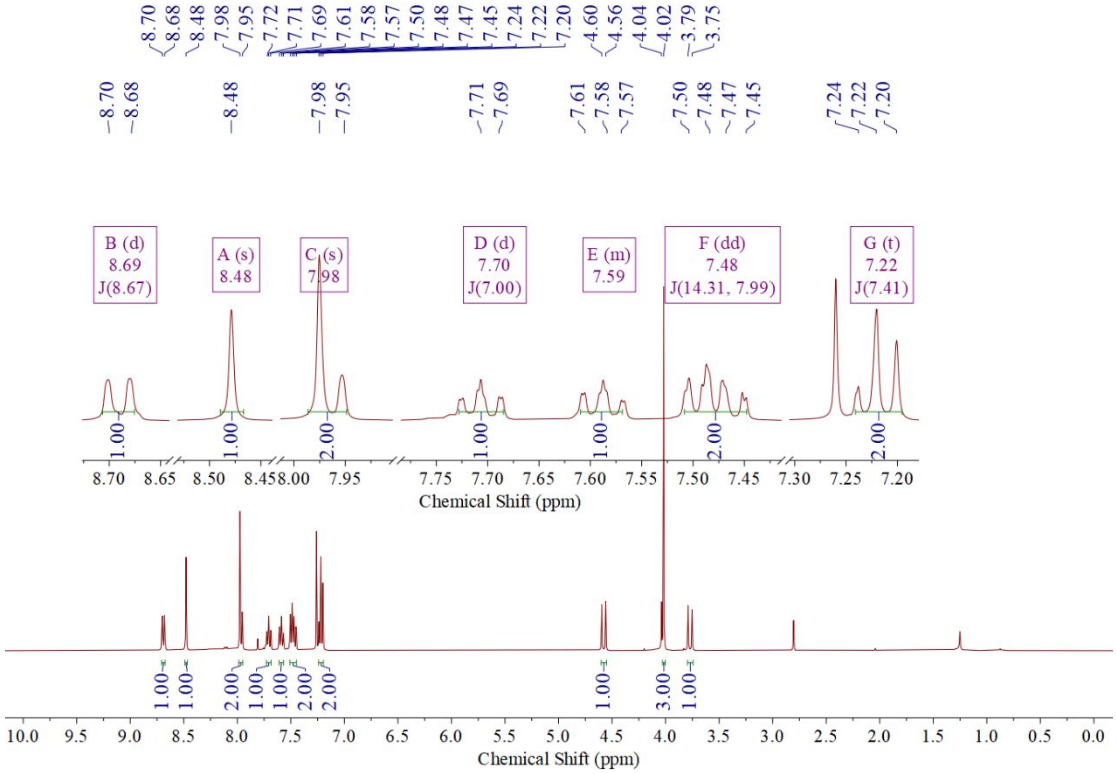
B (d) 8.69 J(8.67)
A (s) 8.48
C (s) 7.98
D (d) 7.70 J(7.00)
E (m) 7.59
F (dd) 7.48 J(14.31, 7.99)
G (t) 7.22 J(7.41)
Chemical Shift (ppm)
Chemical Shift (ppm)

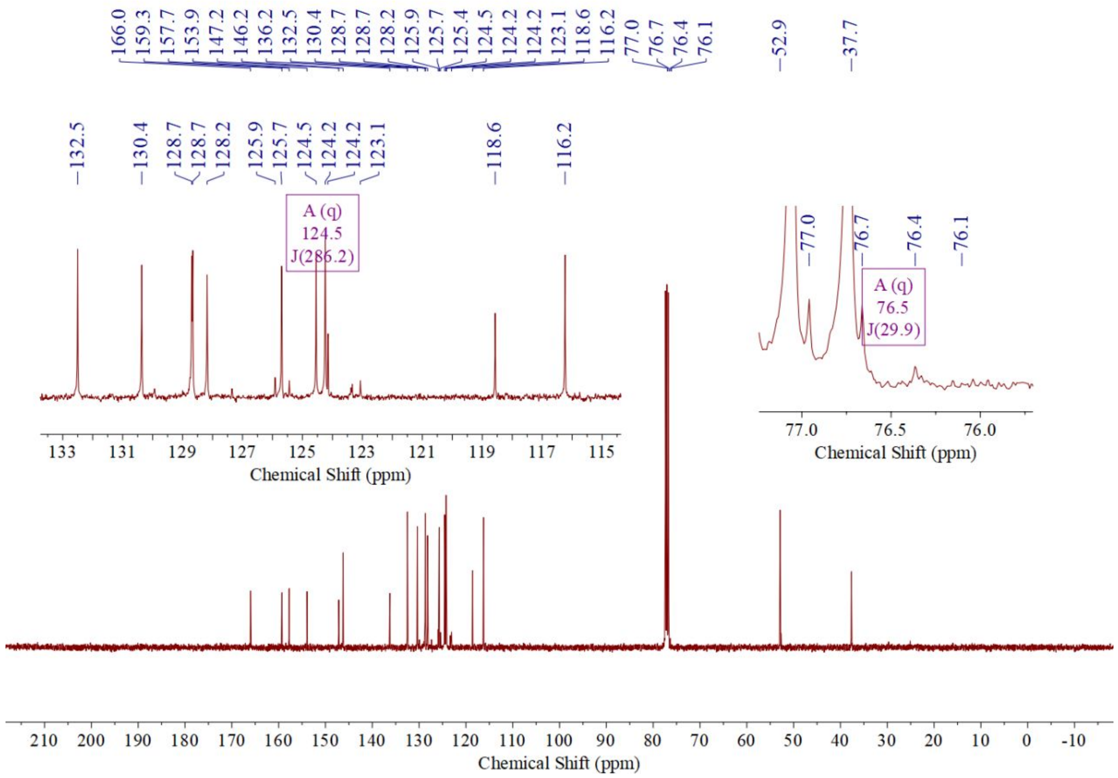
166.0
159.3
157.7
153.9
147.2
146.2
136.2
132.5
130.4
128.7
128.7
128.2
125.9
125.7
125.4
124.5
124.2
124.2
123.1
118.6
116.2
77.0
76.7
76.4
76.1
52.9
37.7
A (q)
124.5
J(286.2)
A (q)
76.5
J(29.9)
Chemical Shift (ppm)

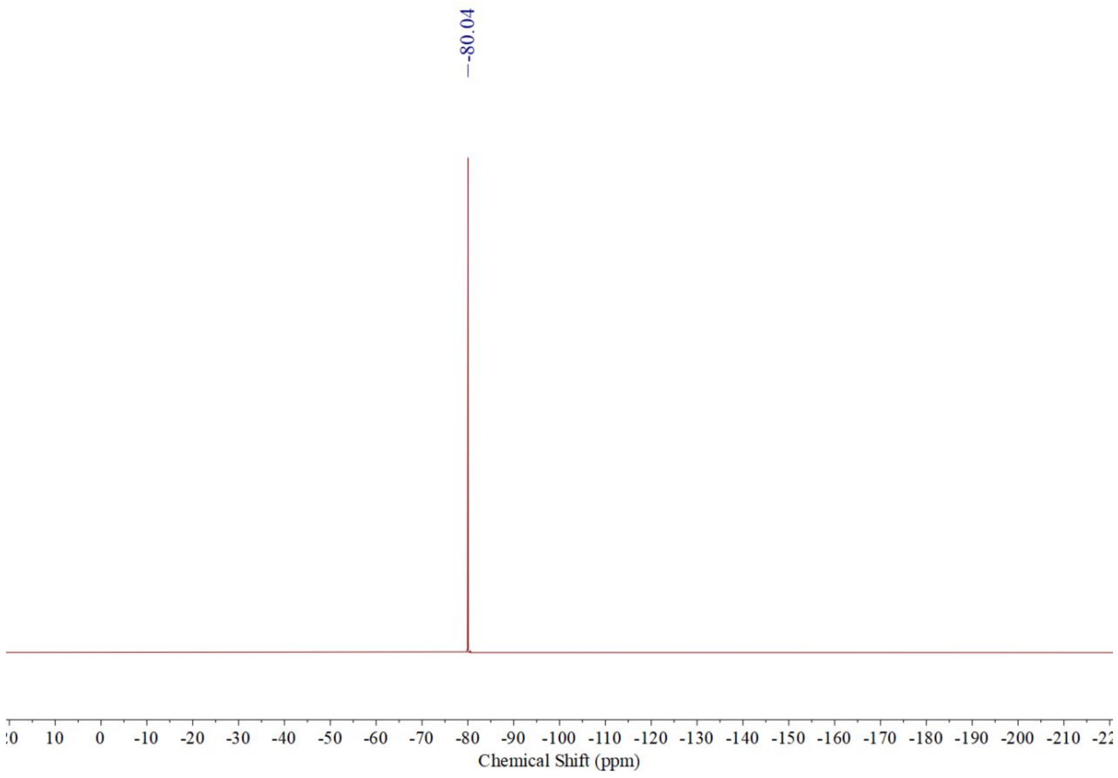
-80.04
Chemical Shift (ppm)

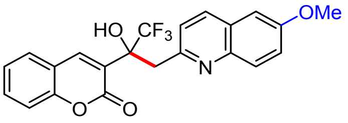
HO
CF3
OMe
N
O
O

(3ae)

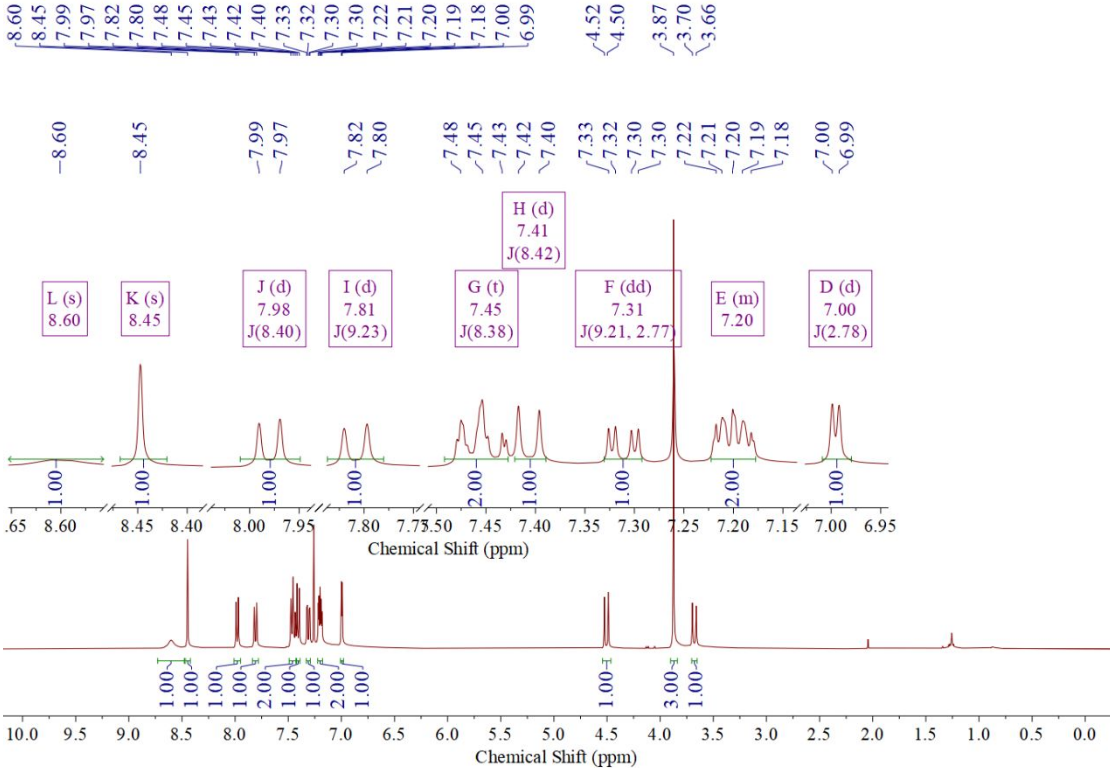
8.60 8.45 7.99 7.97 7.82 7.80 7.48 7.45 7.43 7.42 7.40 7.33 7.32 7.30 7.30 7.22 7.21 7.20 7.19 7.18 7.00 6.99
4.52 4.50
3.87 3.70 3.66
H (d) 7.41 J(8.42)
L (s) 8.60
K (s) 8.45
J (d) 7.98 J(8.40)
I (d) 7.81 J(9.23)
G (t) 7.45 J(8.38)
F (dd) 7.31 J(9.21, 2.77)
E (m) 7.20
D (d) 7.00 J(2.78)
1.00 1.00 1.00 1.00 2.00 1.00 1.00 2.00 1.00
1.00 3.00 1.00
Chemical Shift (ppm)
Chemical Shift (ppm)

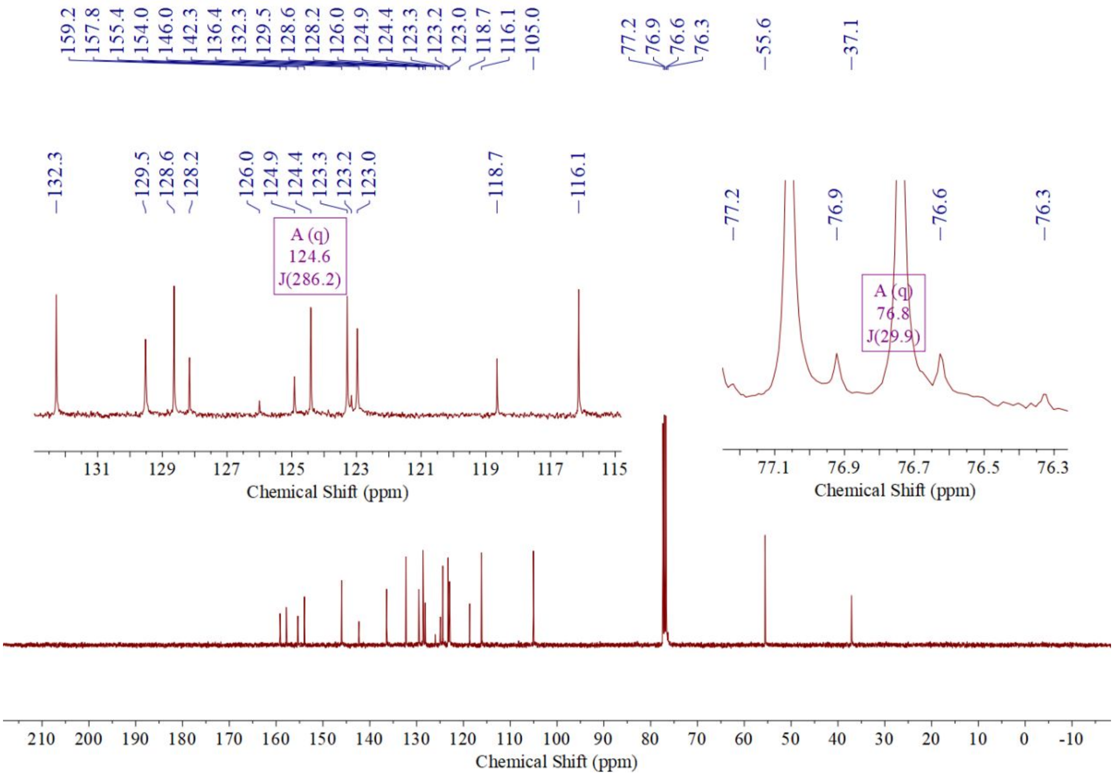
159.2 157.8 155.4 154.0 146.0 142.3 136.4 132.3 129.5 128.6 128.2 126.0 124.9 124.4 123.3 123.2 123.0 118.7 116.1 105.0
77.2 76.9 76.6 76.3
55.6
37.1
A (q) 124.6 J(286.2)
A (q) 76.8 J(29.9)
Chemical Shift (ppm)
Chemical Shift (ppm)
Chemical Shift (ppm)

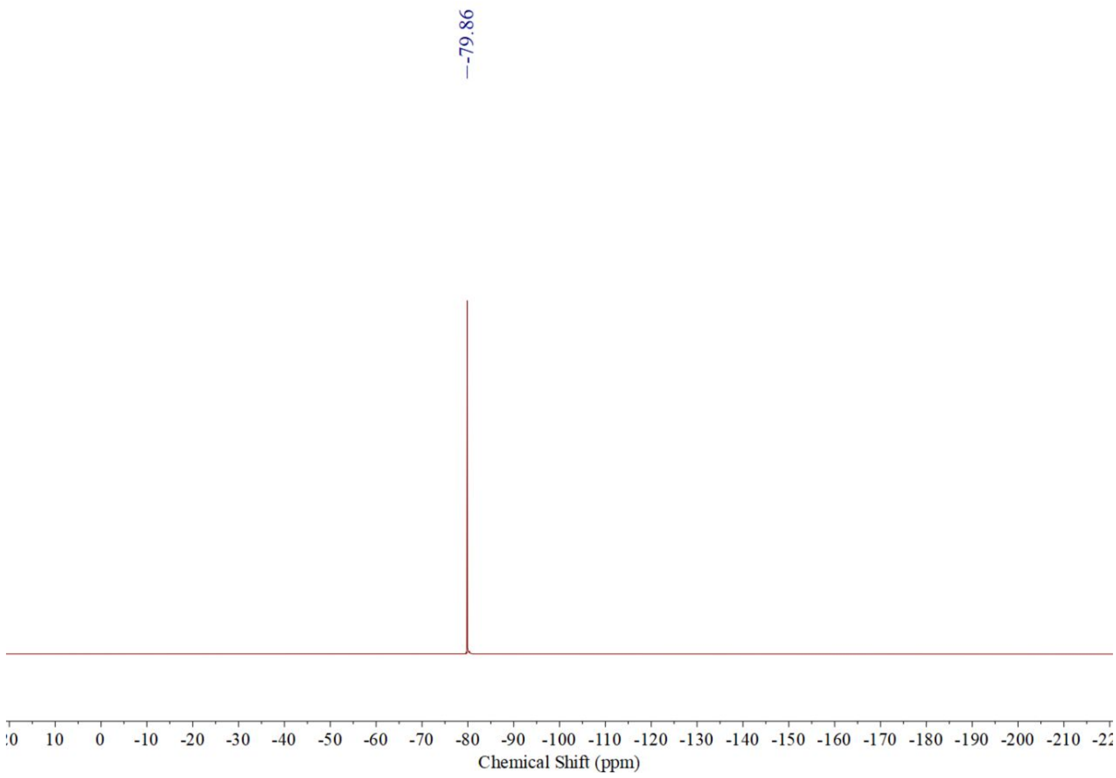
-79.86
Chemical Shift (ppm)

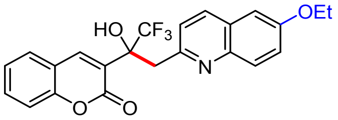

(3af)

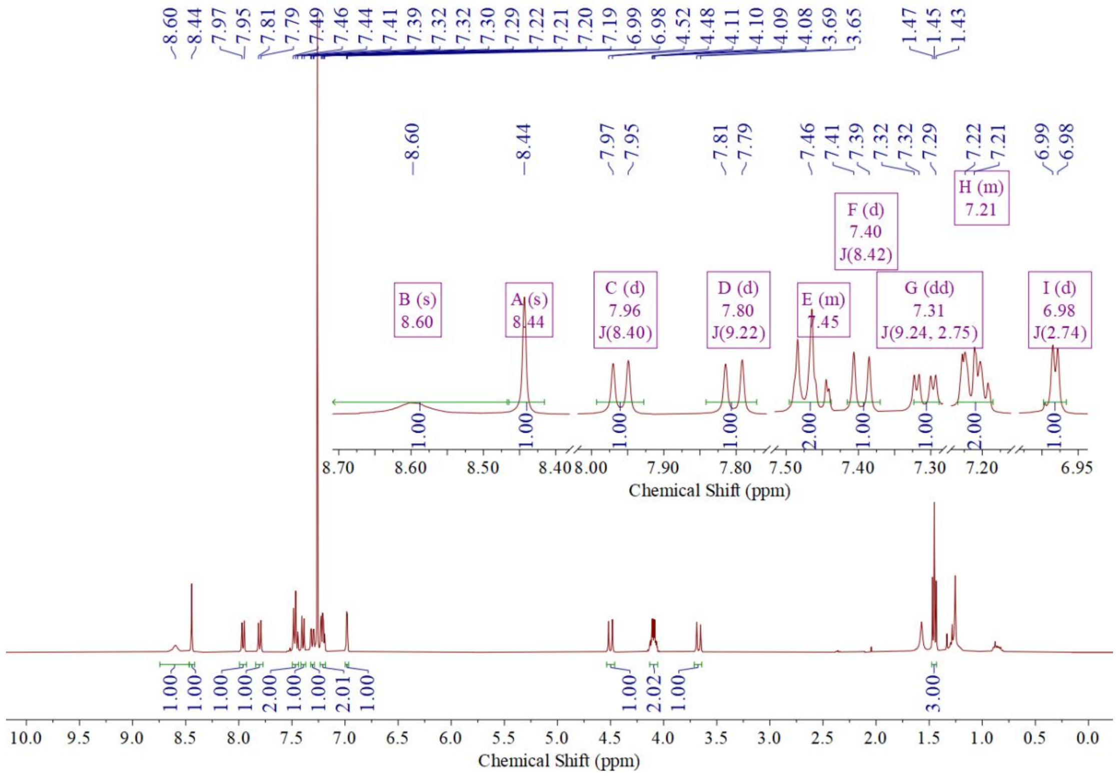
B (s) 8.60
A (s) 8.44
C (d) 7.96 J(8.40)
D (d) 7.80 J(9.22)
E (m) 7.45
F (d) 7.40 J(8.42)
G (dd) 7.31 J(9.24, 2.75)
H (m) 7.21
I (d) 6.98 J(2.74)
Chemical Shift (ppm)

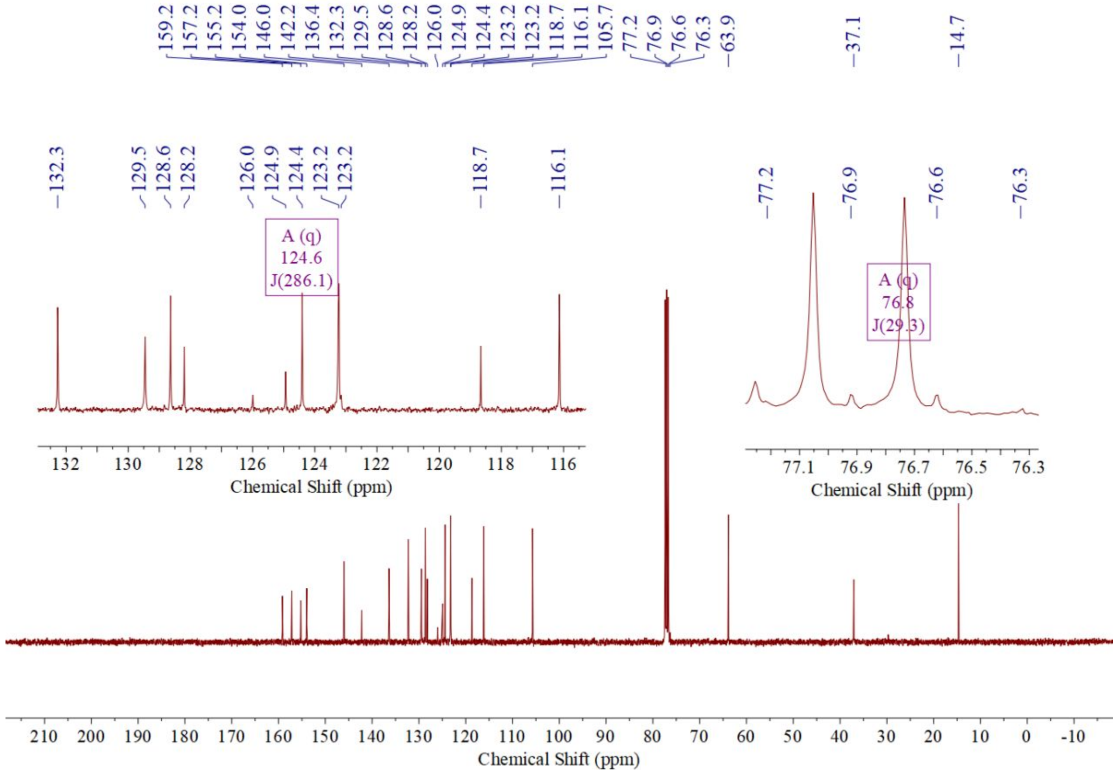

159.2
157.2
155.2
154.0
146.0
142.2
136.4
132.3
129.5
128.6
128.2
126.0
124.9
124.4
123.2
123.2
118.7
116.1
105.7
77.2
76.9
76.6
76.3
63.9
37.1
14.7
A (q)
124.6
J(286.1)
A (q)
76.8
J(29.3)
Chemical Shift (ppm)
Chemical Shift (ppm)
Chemical Shift (ppm)

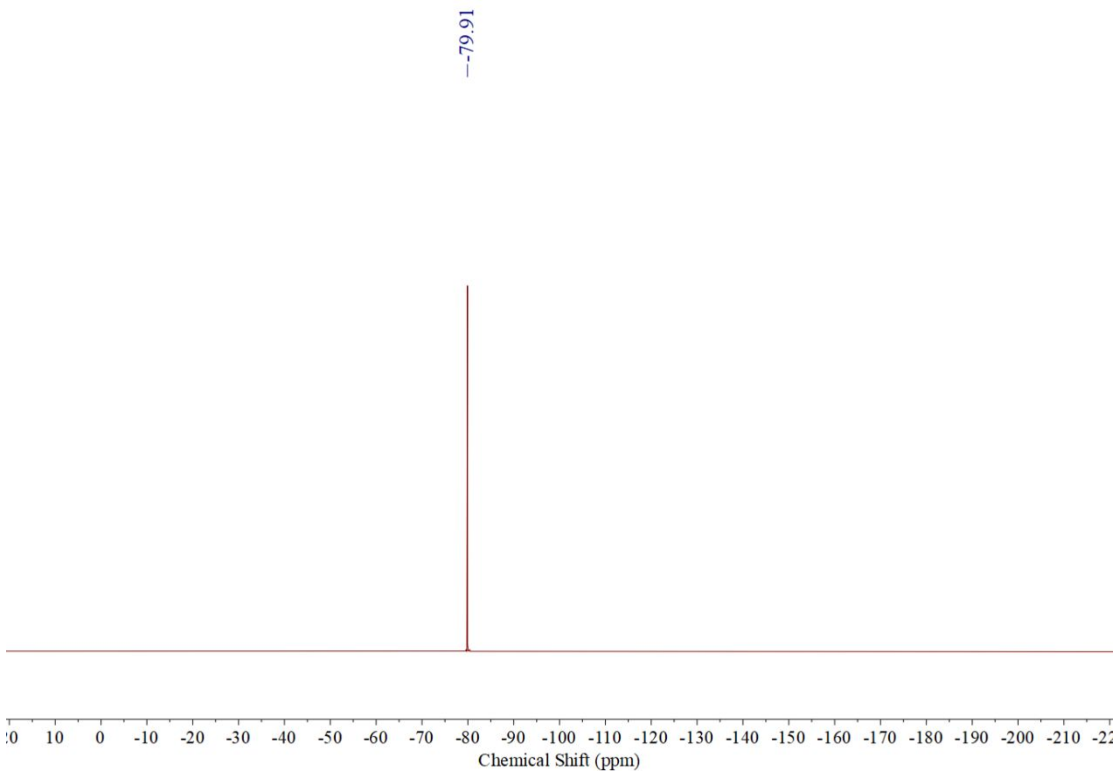

-79.91
Chemical Shift (ppm)

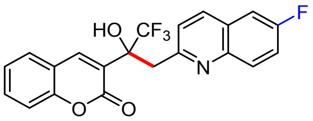
HO
CF3
F
N
O
O

(3ag)

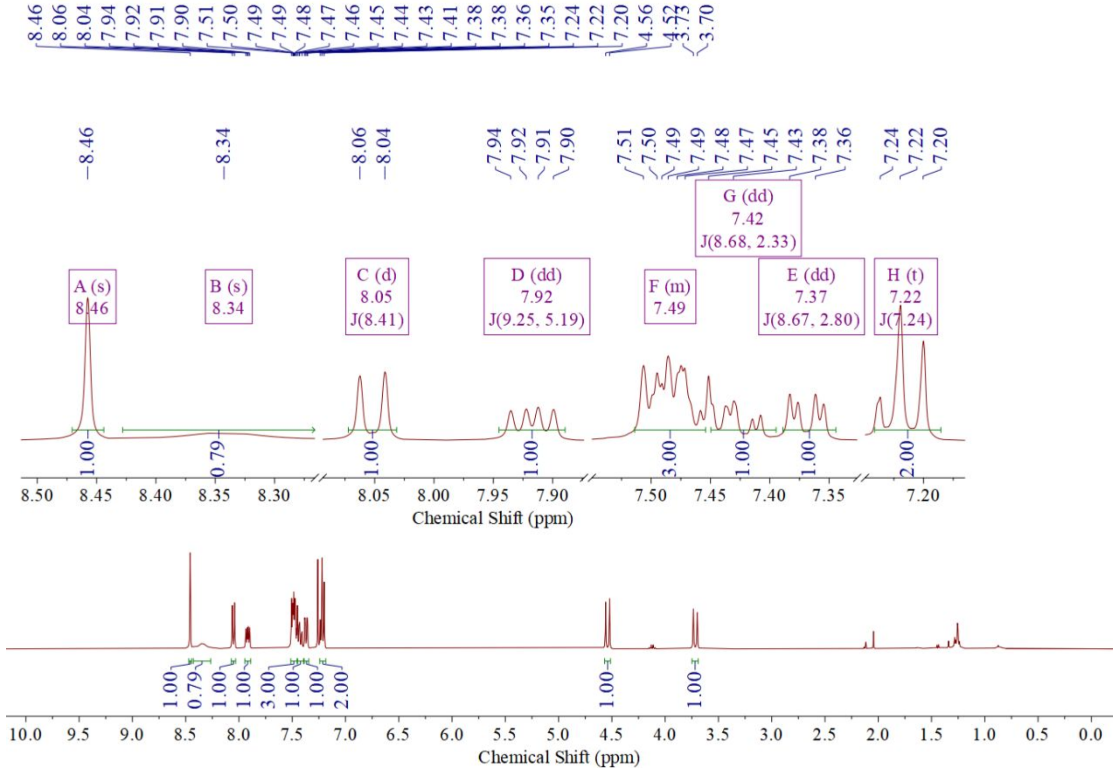
A (s) 8.46
B (s) 8.34
C (d) 8.05 J(8.41)
D (dd) 7.92 J(9.25, 5.19)
F (m) 7.49
G (dd) 7.42 J(8.68, 2.33)
E (dd) 7.37 J(8.67, 2.80)
H (t) 7.22 J(7.24)
Chemical Shift (ppm)
Chemical Shift (ppm)

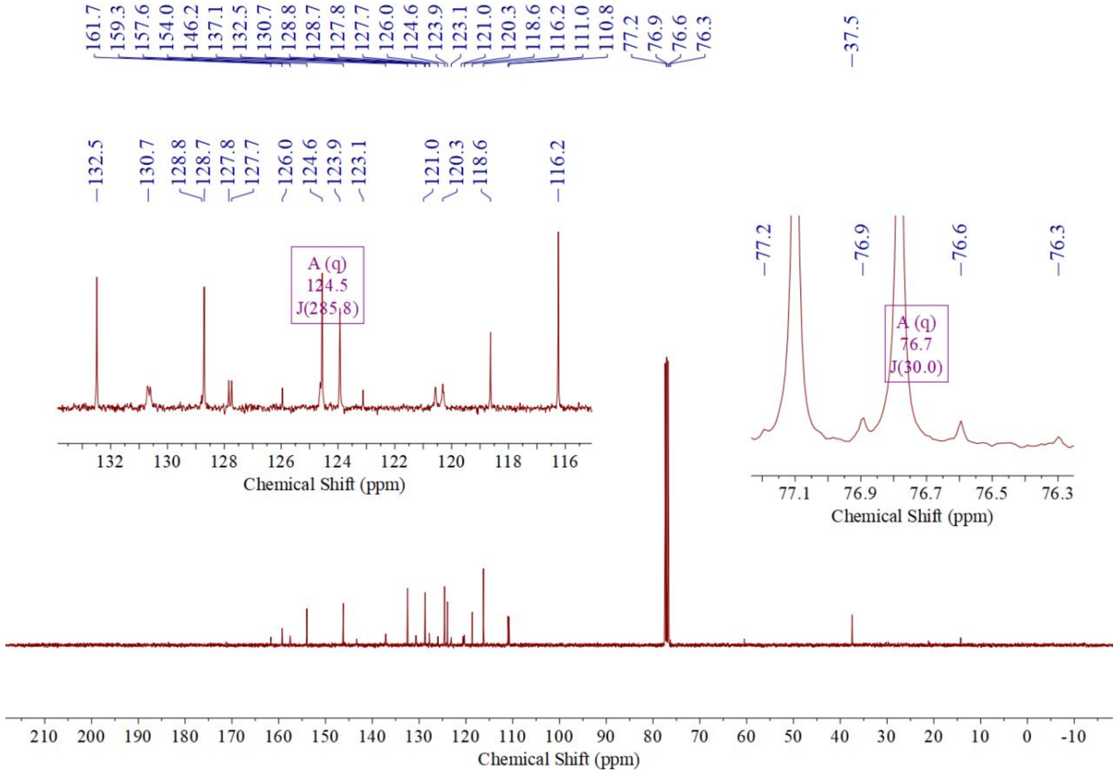
A (q) 124.5 J(285.8)
A (q) 76.7 J(30.0)
Chemical Shift (ppm)
Chemical Shift (ppm)
Chemical Shift (ppm)

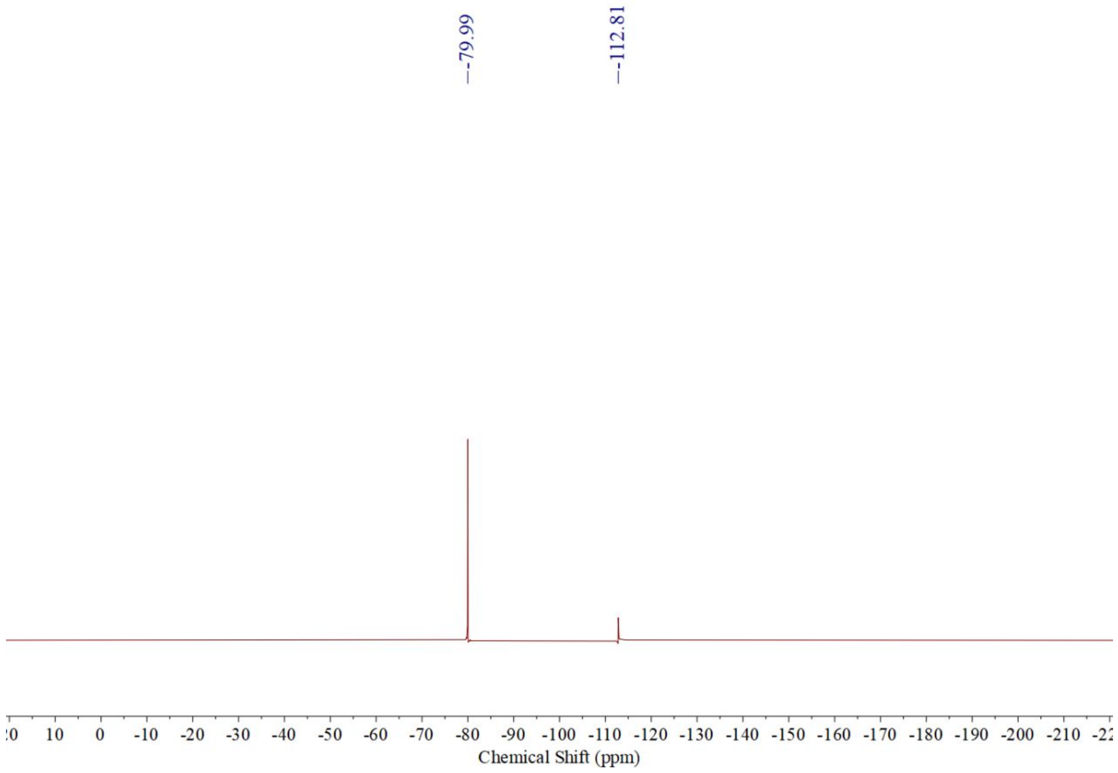
-79.99
-112.81
Chemical Shift (ppm)

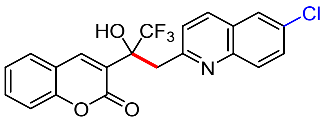
HO
CF3
Cl
N
O
O

(3ah)

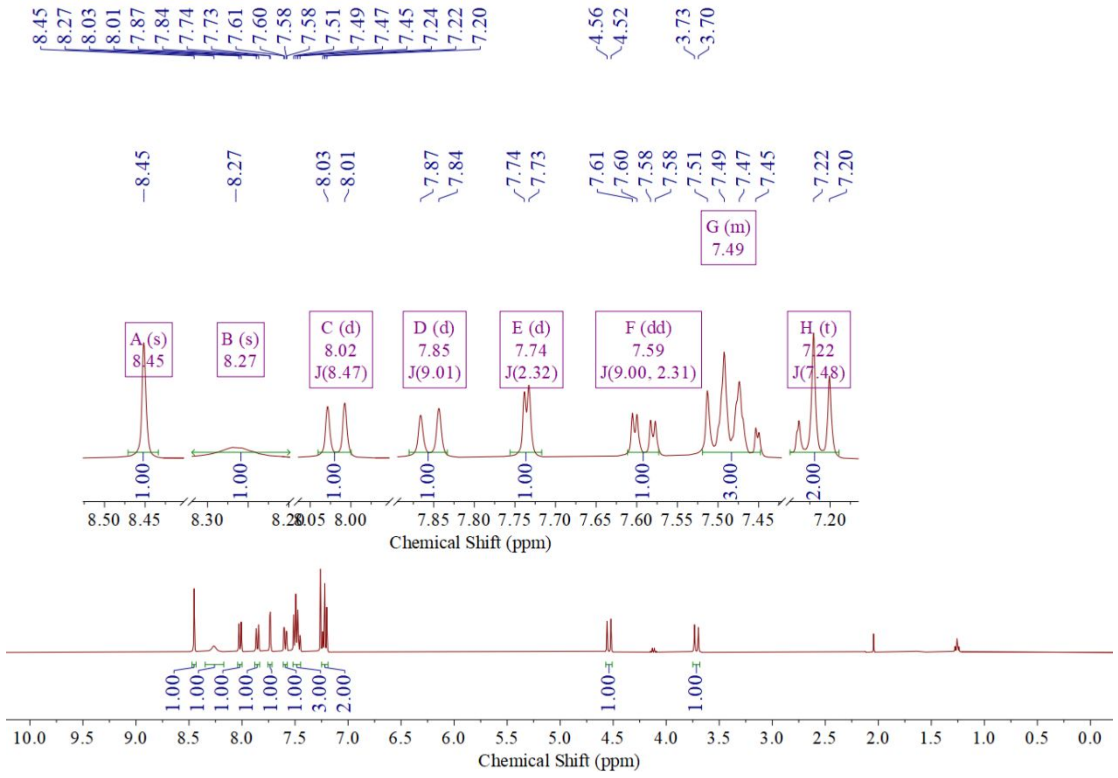
A (s) 8.45
B (s) 8.27
C (d) 8.02 J(8.47)
D (d) 7.85 J(9.01)
E (d) 7.74 J(2.32)
F (dd) 7.59 J(9.00, 2.31)
G (m) 7.49
H (t) 7.22 J(7.48)
Chemical Shift (ppm)

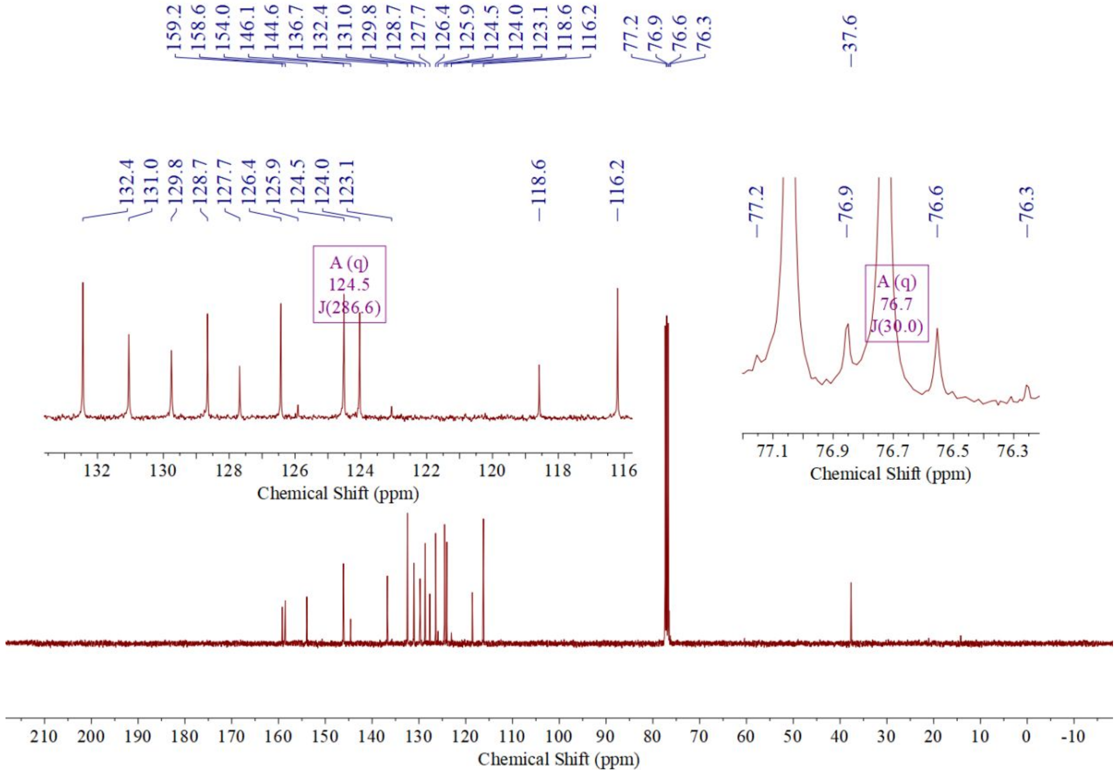
159.2
158.6
154.0
146.1
144.6
136.7
132.4
131.0
129.8
128.7
127.7
126.4
125.9
124.5
124.0
123.1
118.6
116.2
77.2
76.9
76.6
76.3
37.6
A (q)
124.5
J(286.6)
A (q)
76.7
J(30.0)
Chemical Shift (ppm)

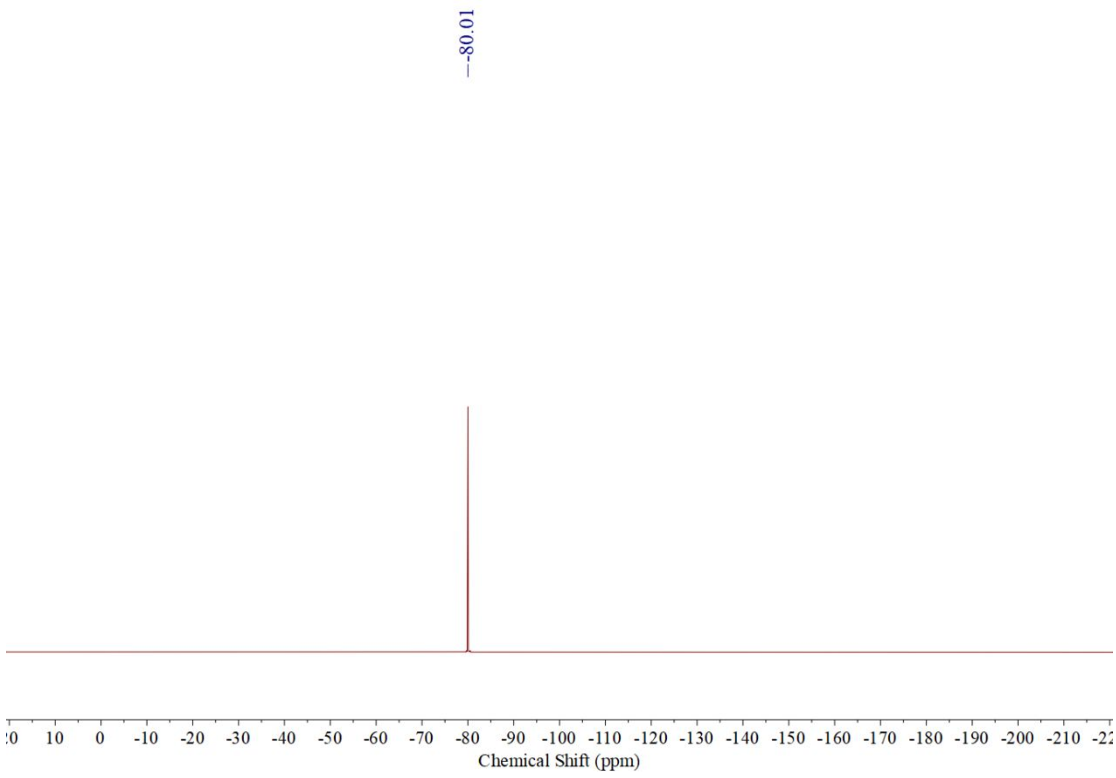
-80.01
Chemical Shift (ppm)

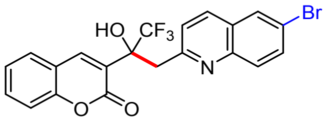

(3ai)

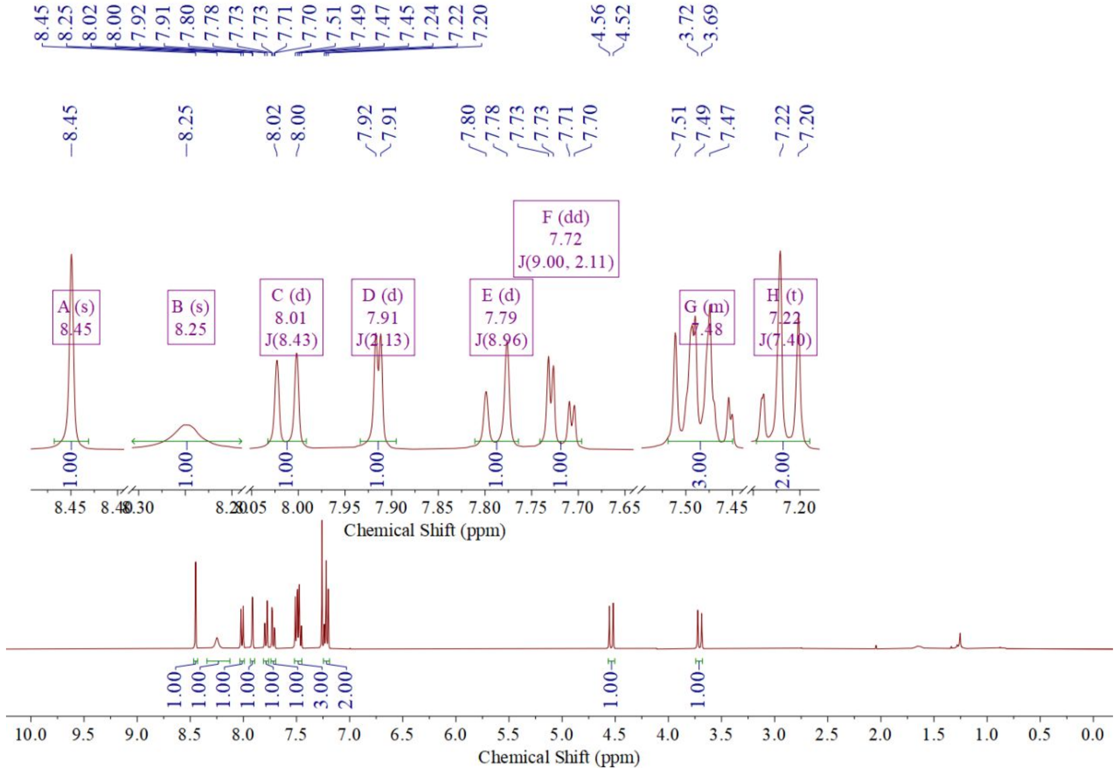

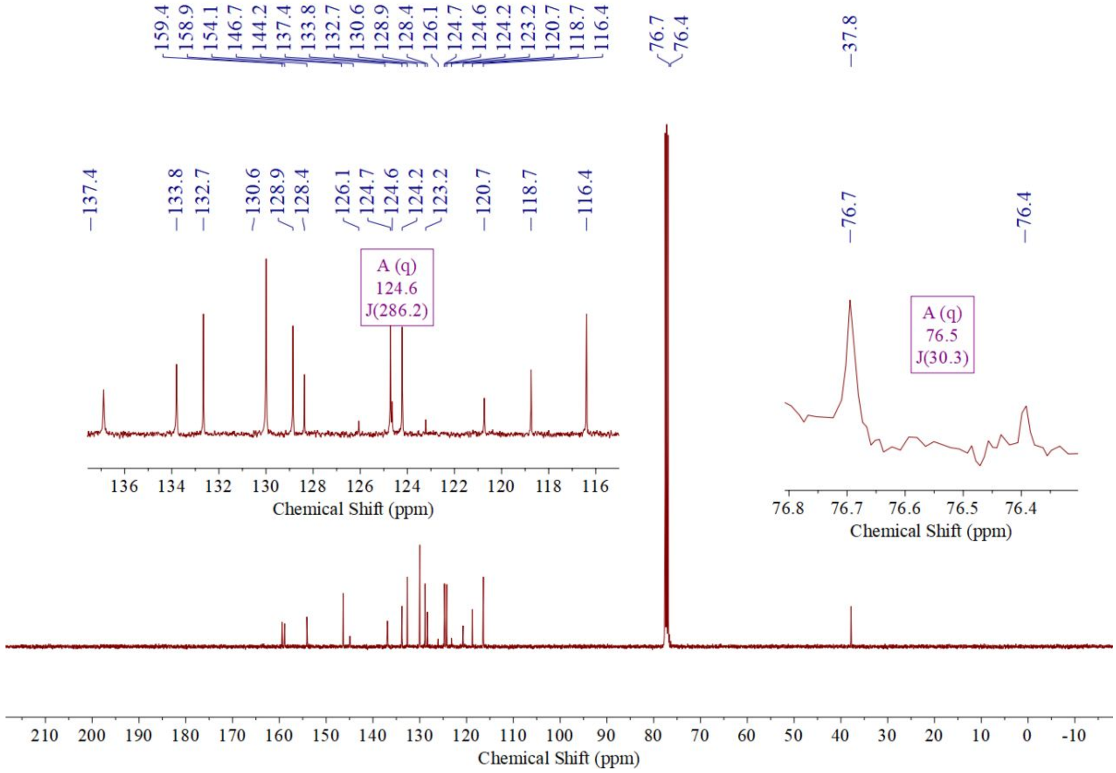

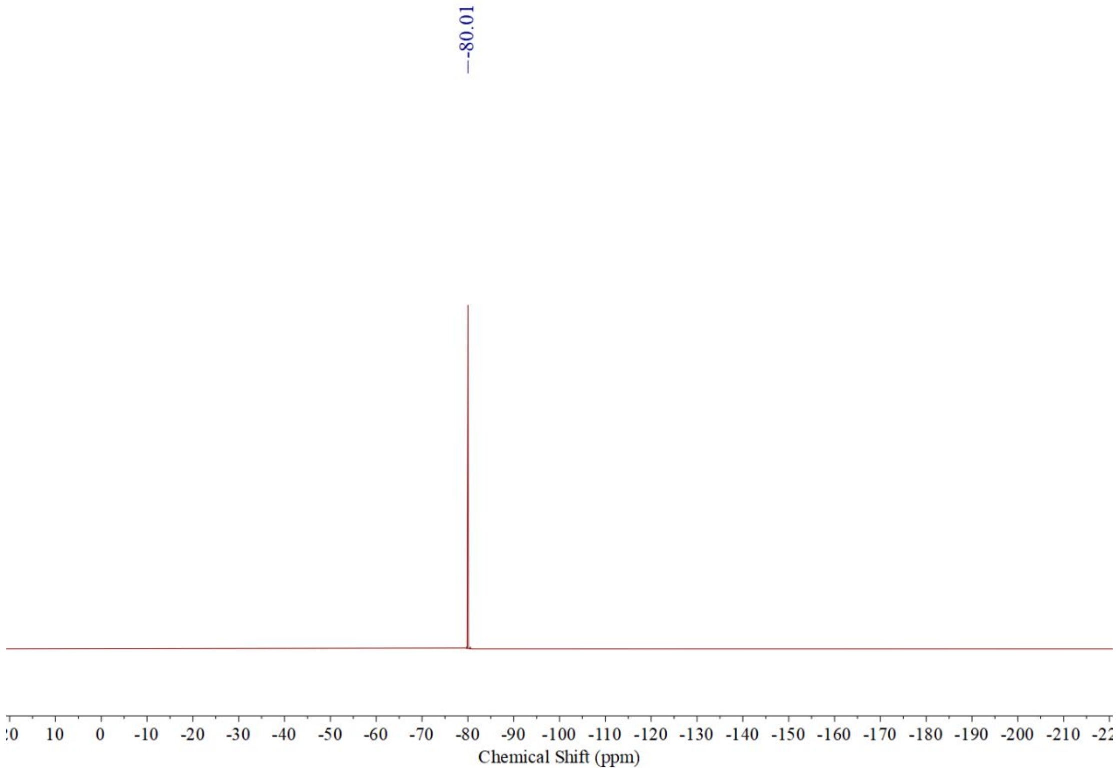
-80.01
20 10 0 -10 -20 -30 -40 -50 -60 -70 -80 -90 -100 -110 -120 -130 -140 -150 -160 -170 -180 -190 -200 -210 -220
Chemical Shift (ppm)

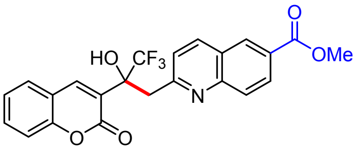
HO
CF3
OMe
O
N
O
O

(3aj)

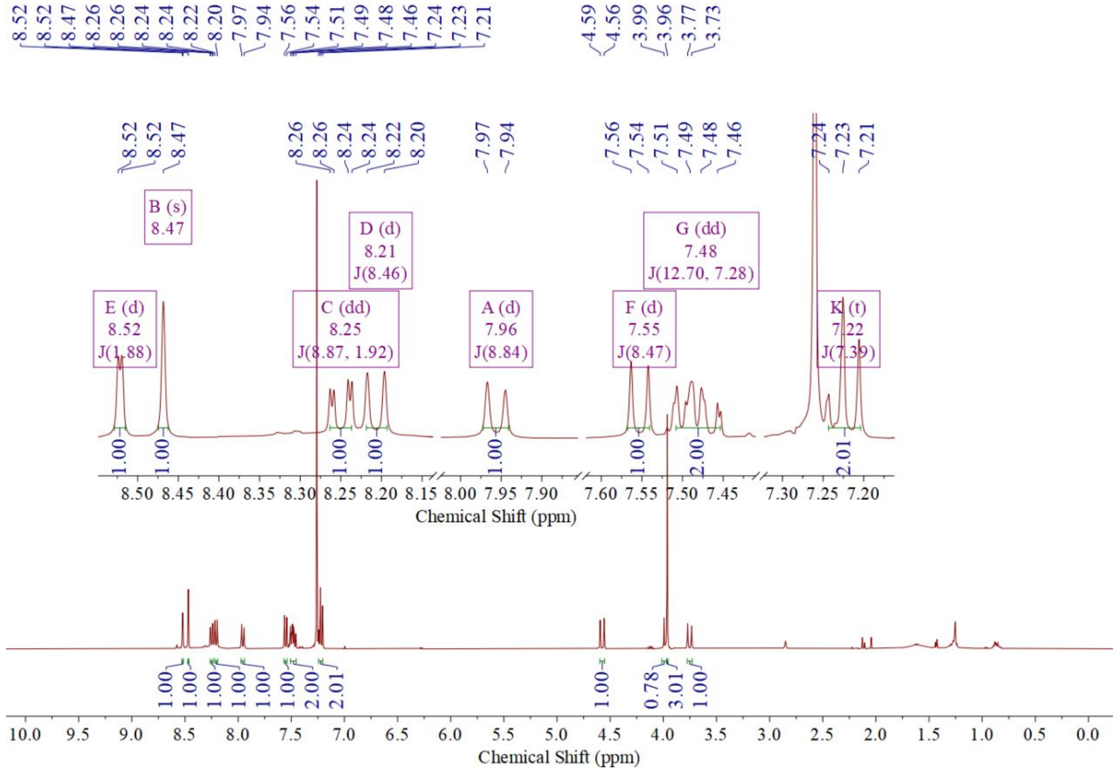
E (d) 8.52 J(1.88)
B (s) 8.47
C (dd) 8.25 J(8.87, 1.92)
D (d) 8.21 J(8.46)
A (d) 7.96 J(8.84)
F (d) 7.55 J(8.47)
G (dd) 7.48 J(12.70, 7.28)
K (t) 7.22 J(7.39)
Chemical Shift (ppm)

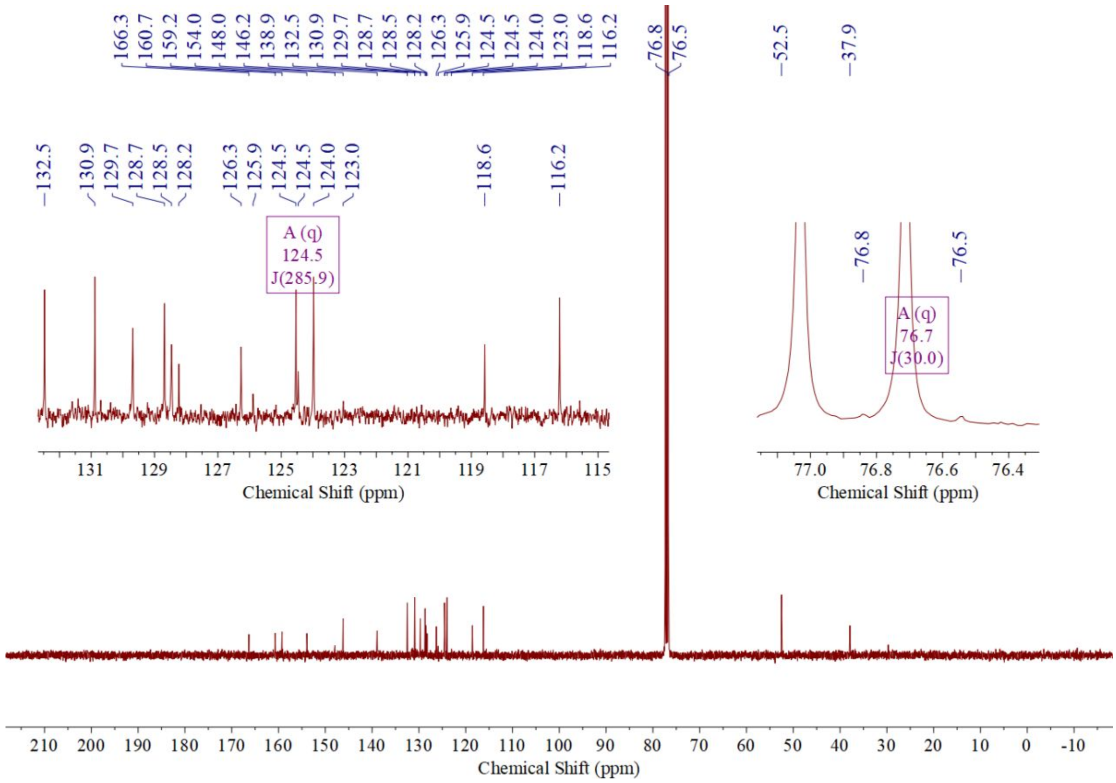
166.3
160.7
159.2
154.0
148.0
146.2
138.9
132.5
130.9
129.7
128.7
128.5
128.2
126.3
125.9
124.5
124.5
124.0
123.0
118.6
116.2
76.8
76.5
52.5
37.9
A (q)
124.5
J(285.9)
A (q)
76.7
J(30.0)
Chemical Shift (ppm)

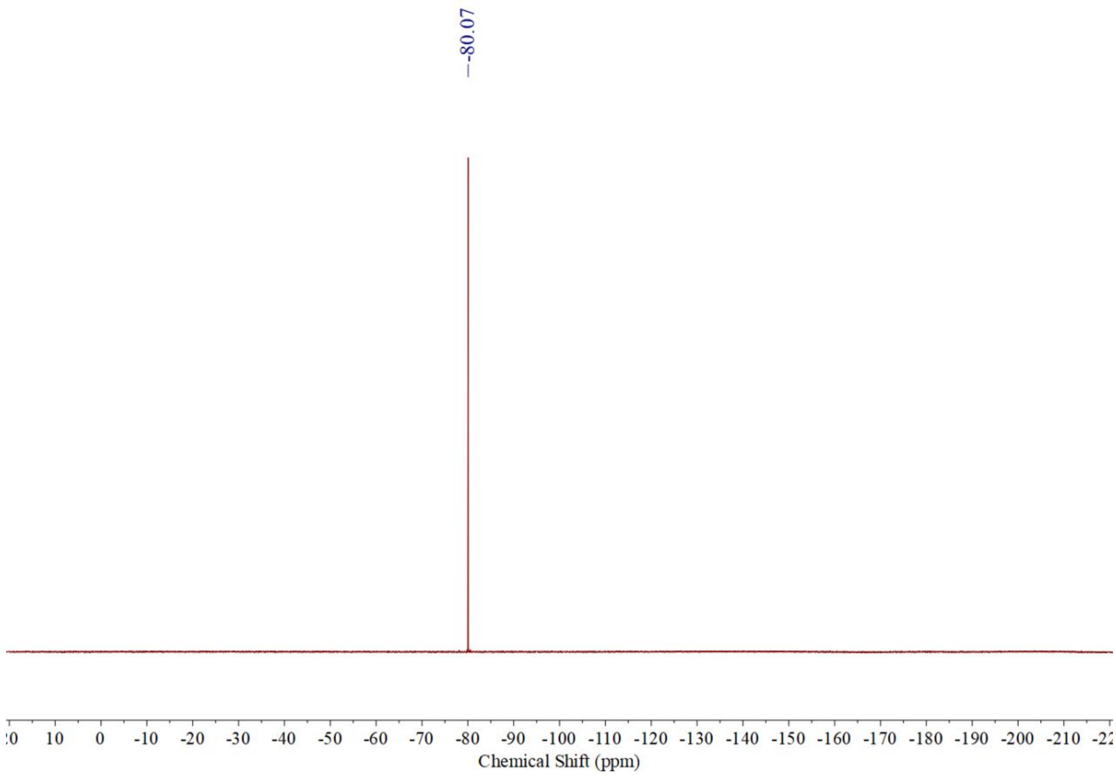
-80.07
Chemical Shift (ppm)

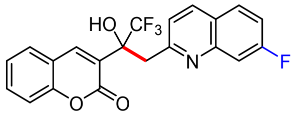

HO
CF3
N
F
O
O

(3ak)

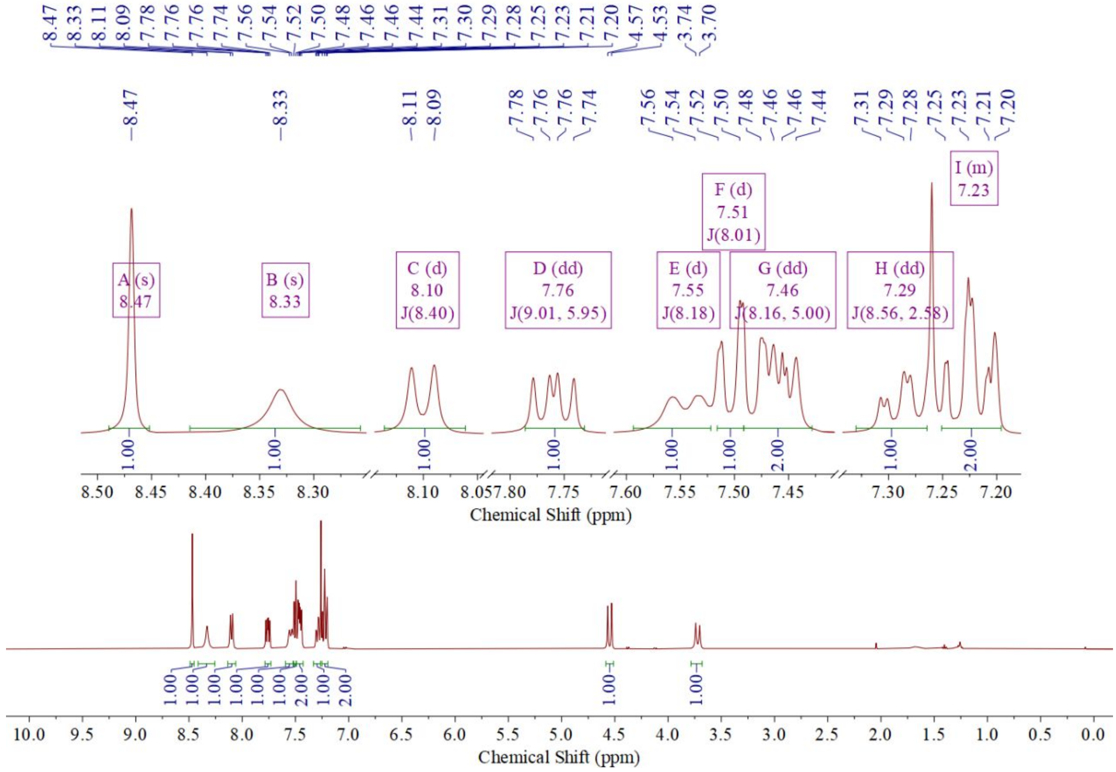

8.47
8.33
8.11
8.09
7.78
7.76
7.76
7.74
7.56
7.54
7.52
7.50
7.48
7.46
7.46
7.44
7.31
7.30
7.29
7.28
7.25
7.23
7.21
7.20
4.57
4.53
3.74
3.70
A (s) 8.47
B (s) 8.33
C (d) 8.10 J(8.40)
D (dd) 7.76 J(9.01, 5.95)
E (d) 7.55 J(8.18)
F (d) 7.51 J(8.01)
G (dd) 7.46 J(8.16, 5.00)
H (dd) 7.29 J(8.56, 2.58)
I (m) 7.23
Chemical Shift (ppm)
Chemical Shift (ppm)

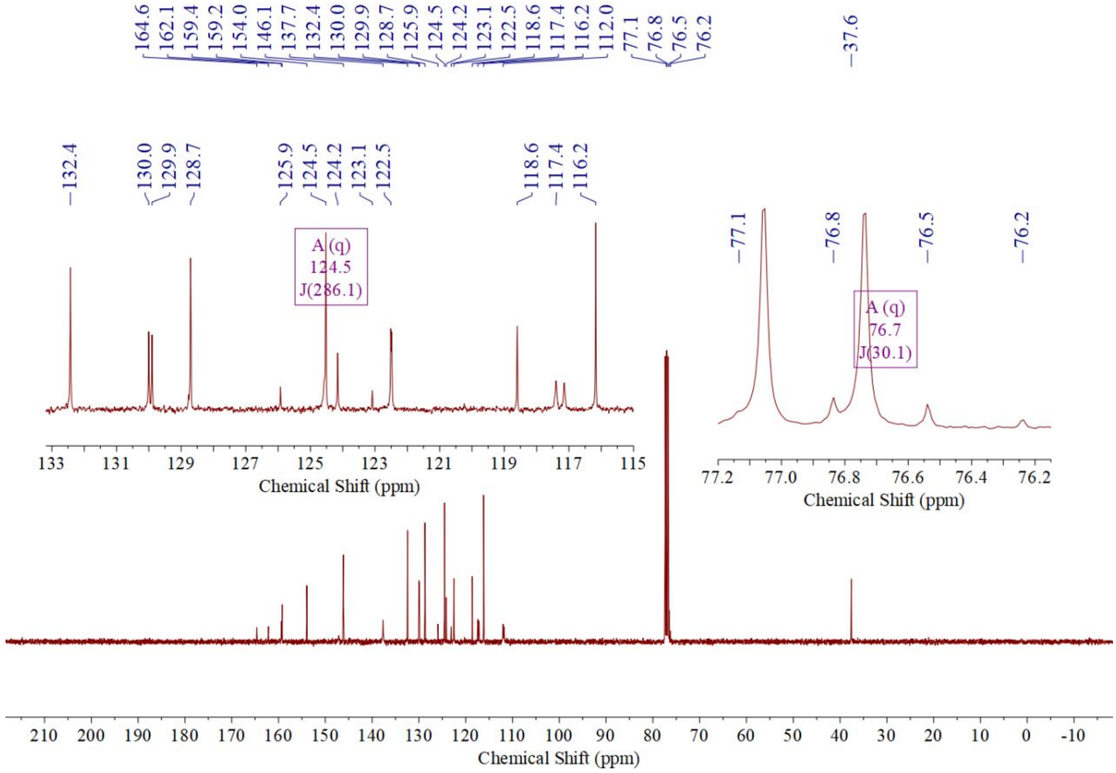

164.6
162.1
159.4
159.2
154.0
146.1
137.7
132.4
130.0
129.9
128.7
125.9
124.5
124.2
123.1
122.5
118.6
117.4
116.2
112.0
77.1
76.8
76.5
76.2
37.6
A (q) 124.5 J(286.1)
A (q) 76.7 J(30.1)
Chemical Shift (ppm)
Chemical Shift (ppm)
Chemical Shift (ppm)

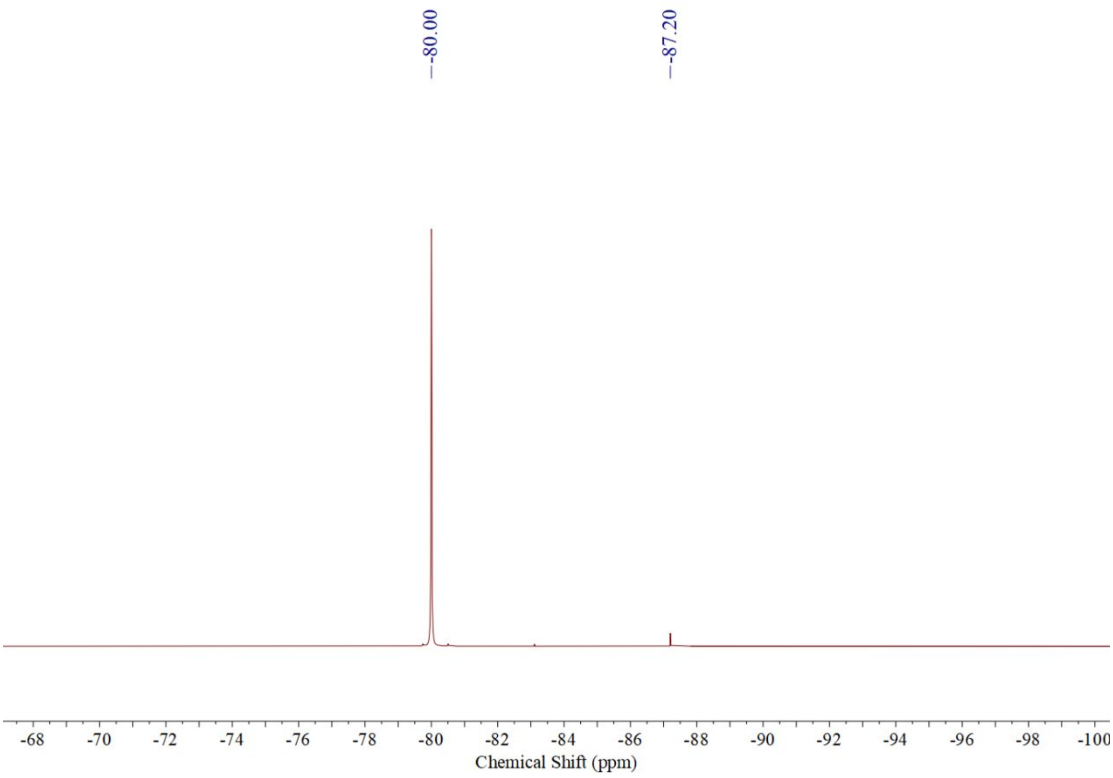
-80.00
-87.20
-68 -70 -72 -74 -76 -78 -80 -82 -84 -86 -88 -90 -92 -94 -96 -98 -100
Chemical Shift (ppm)

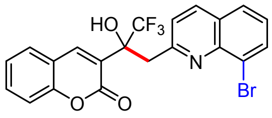
HO
CF3
N
Br
O
O

(3al)

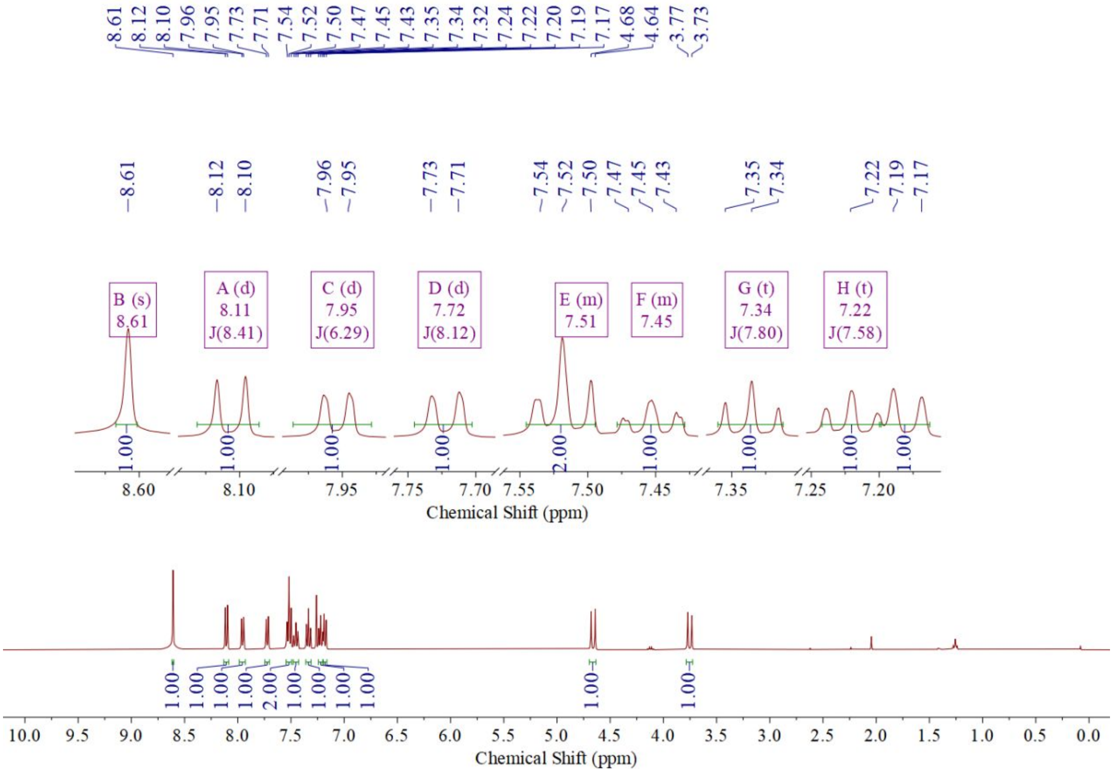
8.61 8.12 8.10 7.96 7.95 7.73 7.71 7.54 7.52 7.50 7.47 7.45 7.43 7.35 7.34 7.32 7.24 7.22 7.20 7.19 7.17 4.68 4.64 3.77 3.73
B (s) 8.61
A (d) 8.11 J(8.41)
C (d) 7.95 J(6.29)
D (d) 7.72 J(8.12)
E (m) 7.51
F (m) 7.45
G (t) 7.34 J(7.80)
H (t) 7.22 J(7.58)
Chemical Shift (ppm)
10.0 9.5 9.0 8.5 8.0 7.5 7.0 6.5 6.0 5.5 5.0 4.5 4.0 3.5 3.0 2.5 2.0 1.5 1.0 0.5 0.0
Chemical Shift (ppm)

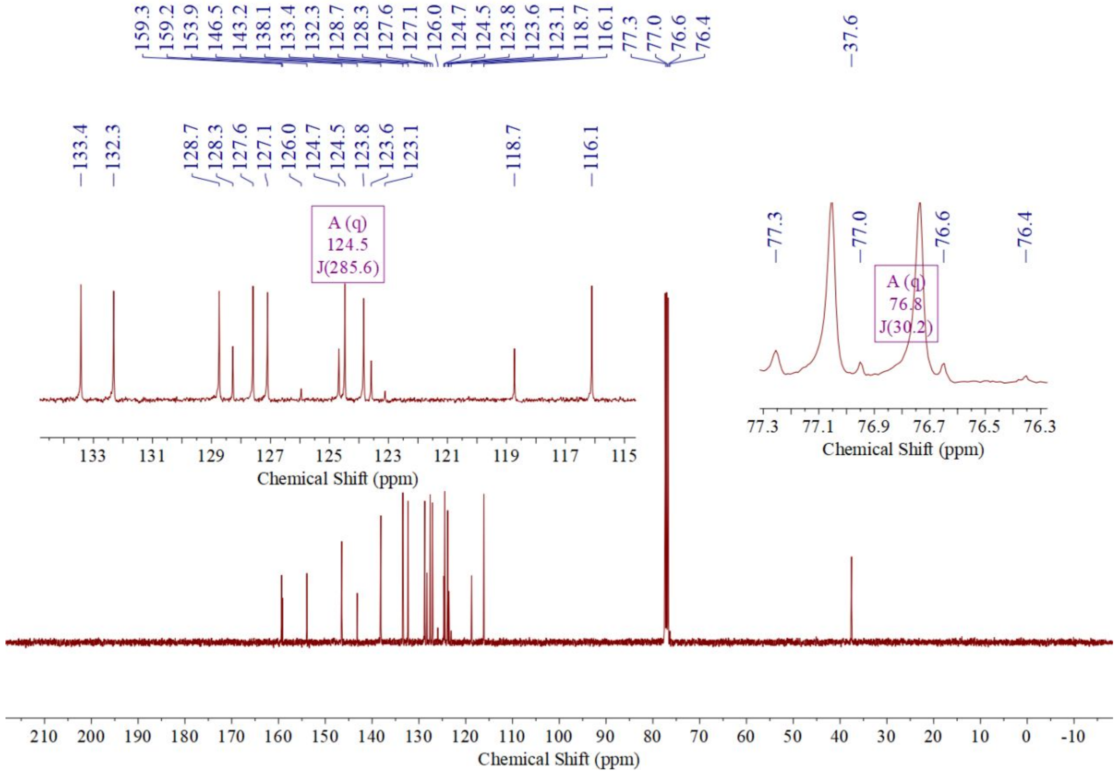
159.3
159.2
153.9
146.5
143.2
138.1
133.4
132.3
128.7
128.3
127.6
127.1
126.0
124.7
124.5
123.8
123.6
123.1
118.7
116.1
77.3
77.0
76.6
76.4
37.6
A (q)
124.5
J(285.6)
A (q)
76.8
J(30.2)
Chemical Shift (ppm)

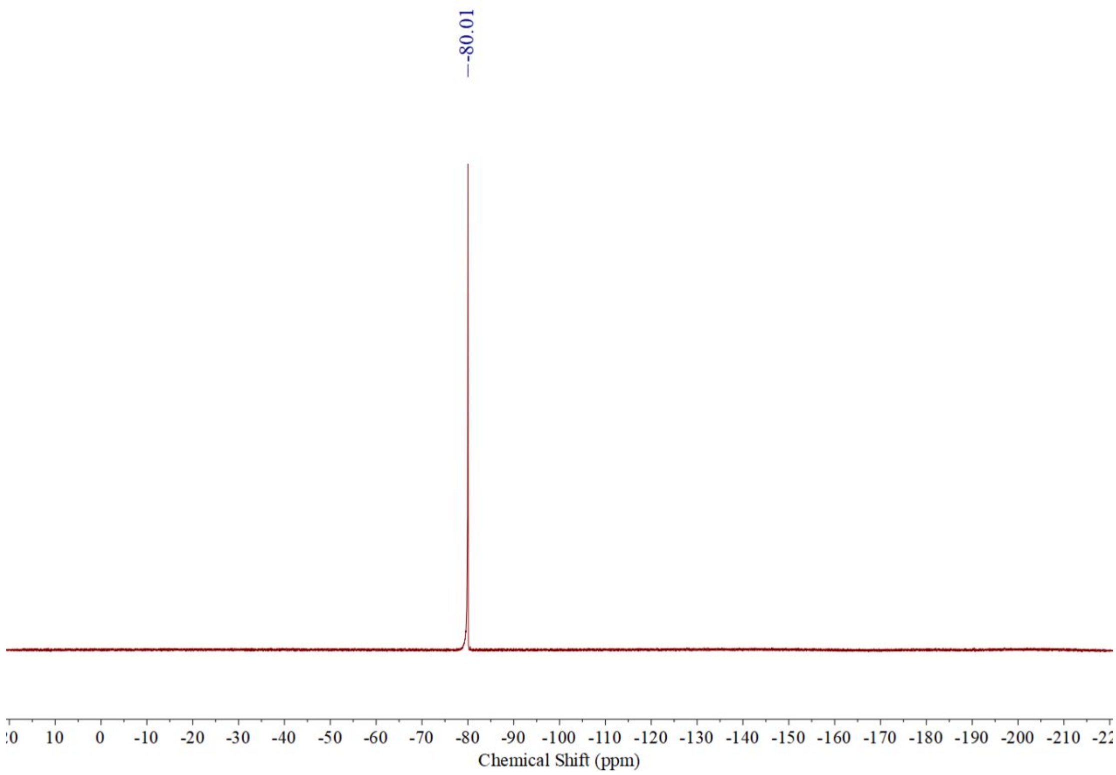
-80.01
Chemical Shift (ppm)

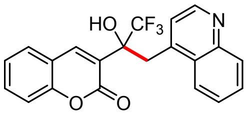

(3am)

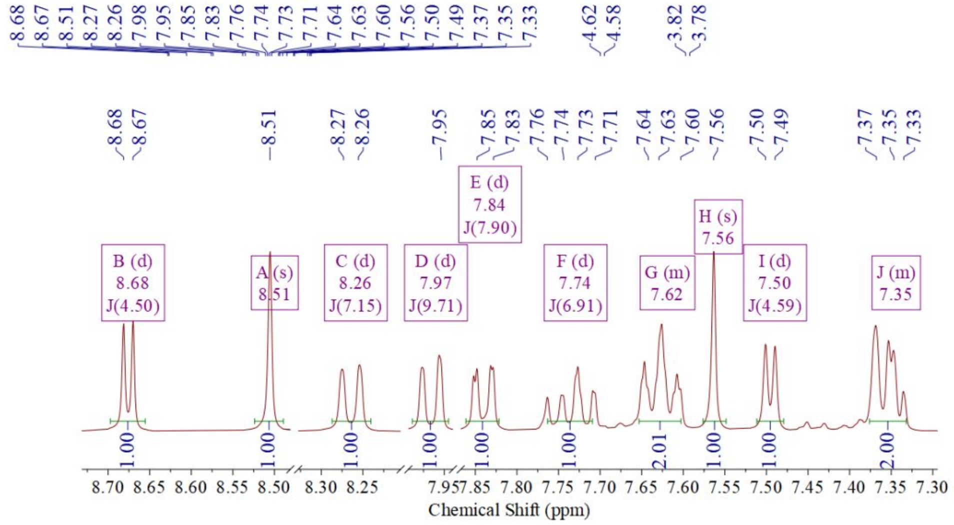

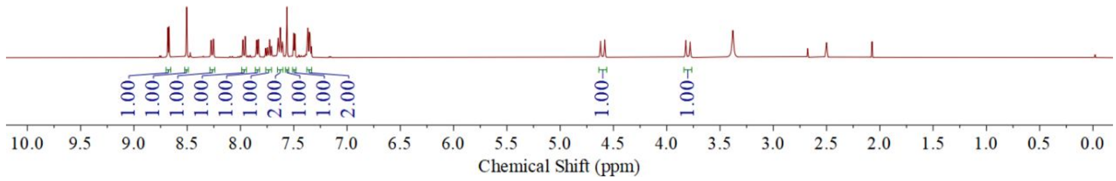

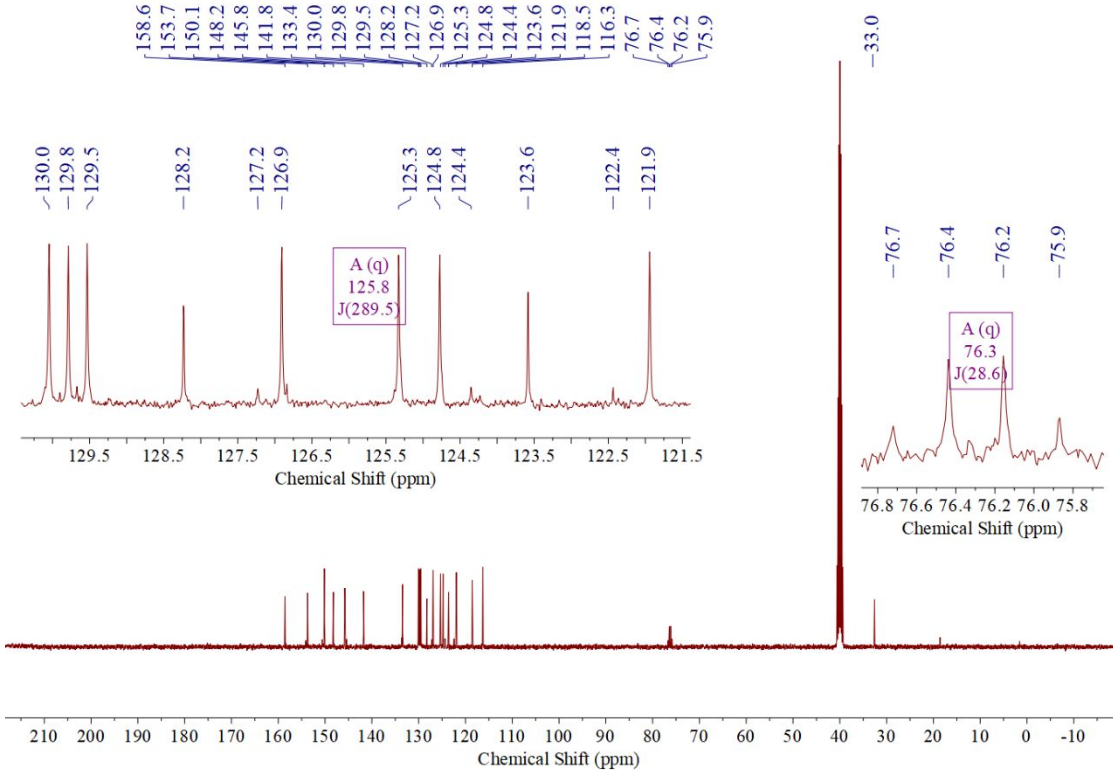

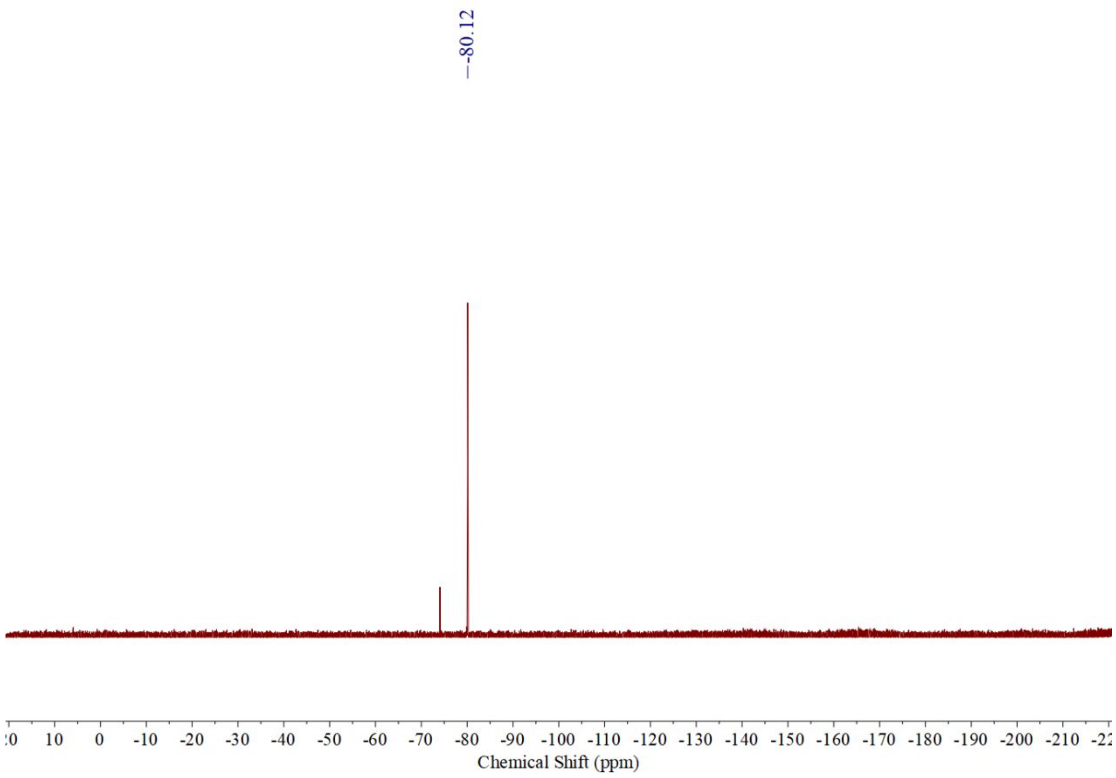
-80.12
Chemical Shift (ppm)

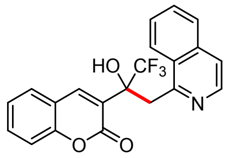
HO
CF3
N
O
O

(3an)

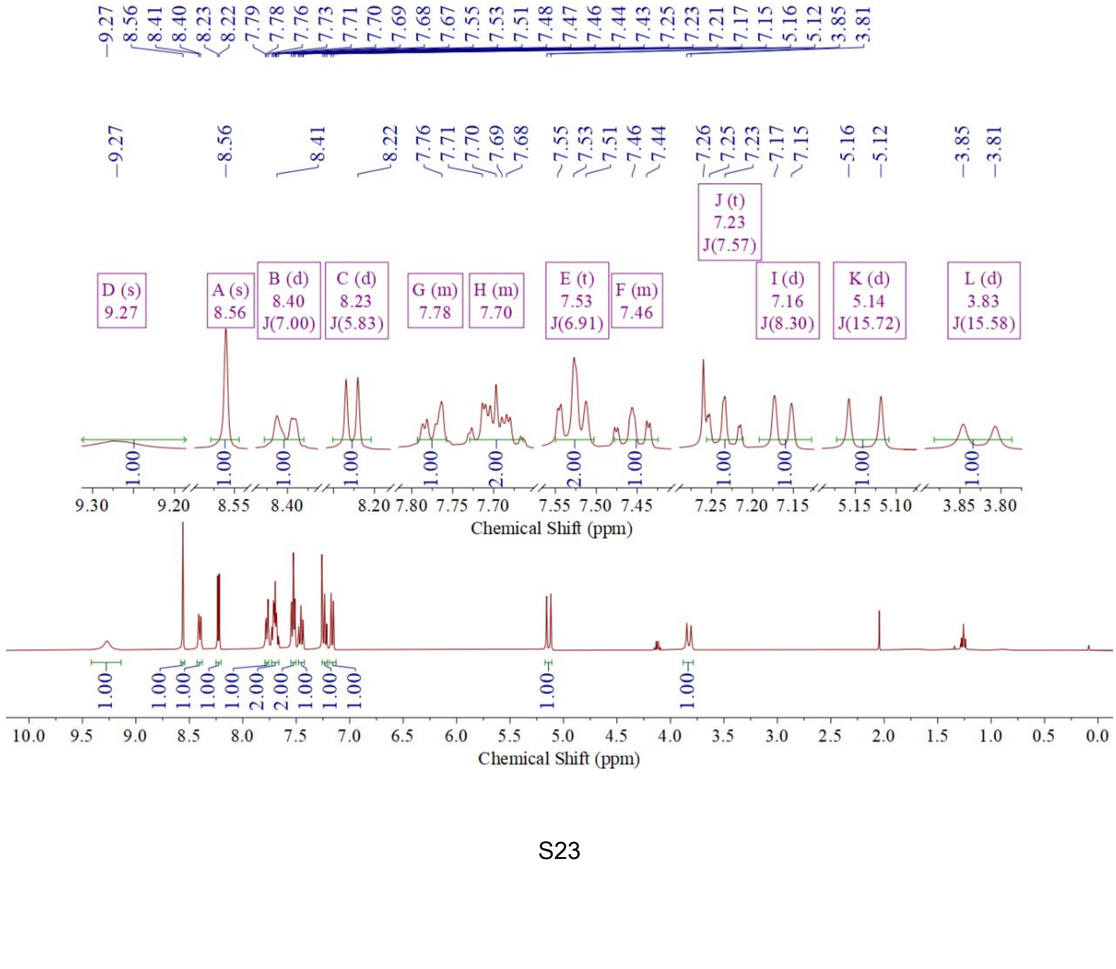
D (s)
9.27
A (s)
8.56
B (d)
8.40
J(7.00)
C (d)
8.23
J(5.83)
G (m)
7.78
H (m)
7.70
E (t)
7.53
J(6.91)
F (m)
7.46
J (t)
7.23
J(7.57)
I (d)
7.16
J(8.30)
K (d)
5.14
J(15.72)
L (d)
3.83
J(15.58)
Chemical Shift (ppm)

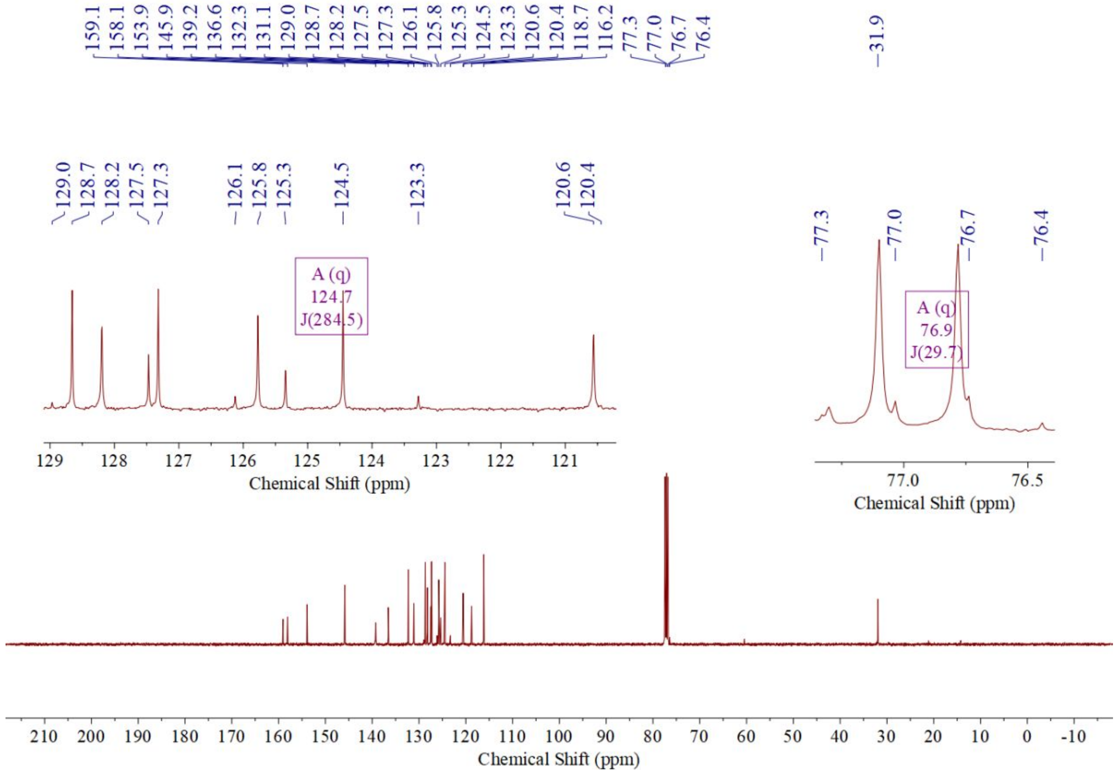
159.1
158.1
153.9
145.9
139.2
136.6
132.3
131.1
129.0
128.7
128.2
127.5
127.3
126.1
125.8
125.3
124.5
123.3
120.6
120.4
118.7
116.2
77.3
77.0
76.7
76.4
31.9
A (q)
124.7
J(284.5)
A (q)
76.9
J(29.7)
Chemical Shift (ppm)

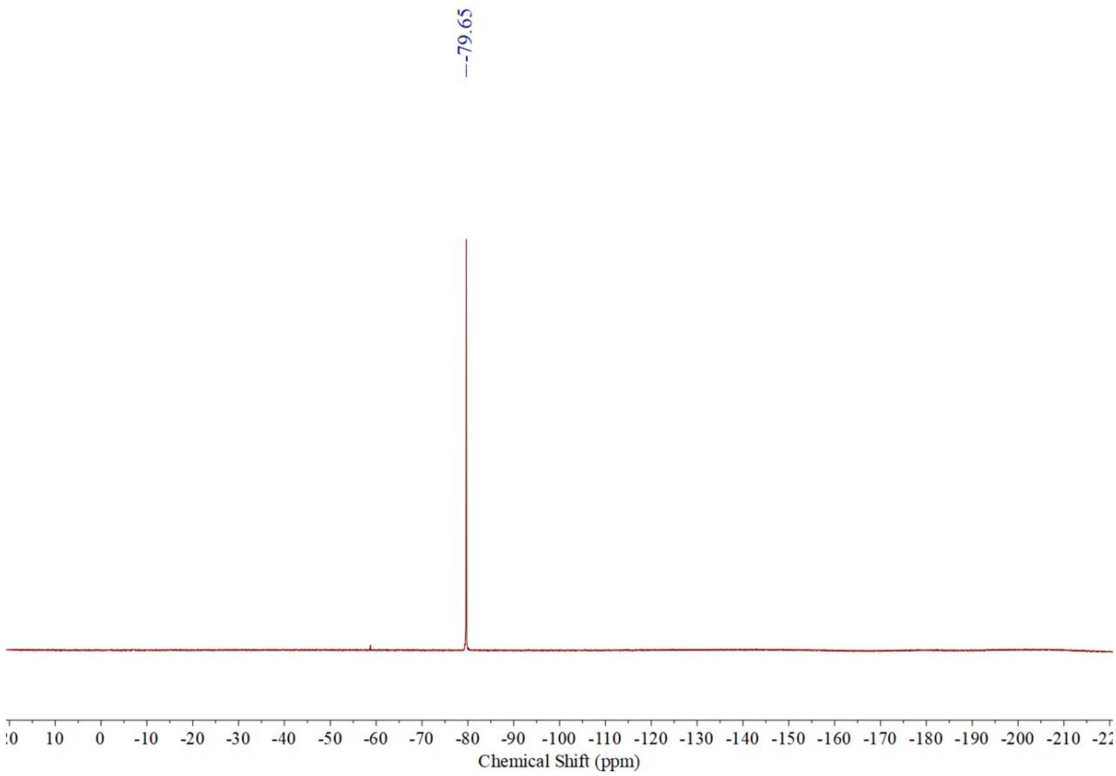
-79.65
Chemical Shift (ppm)

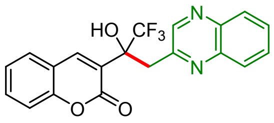

(3ao)

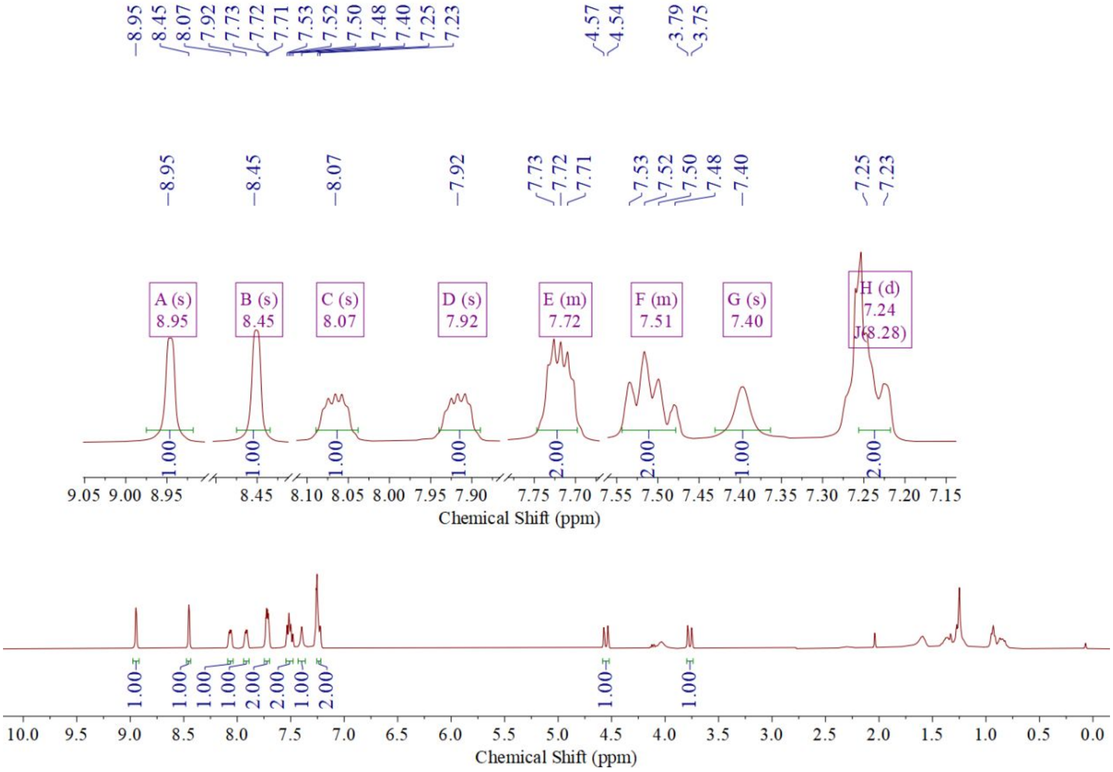
8.95 8.45 8.07 7.92 7.73 7.72 7.71 7.53 7.52 7.50 7.48 7.40 7.25 7.23 4.57 4.54 3.79 3.75
A (s) 8.95
B (s) 8.45
C (s) 8.07
D (s) 7.92
E (m) 7.72
F (m) 7.51
G (s) 7.40
H (d) 7.24 J(8.28)
Chemical Shift (ppm)
Chemical Shift (ppm)

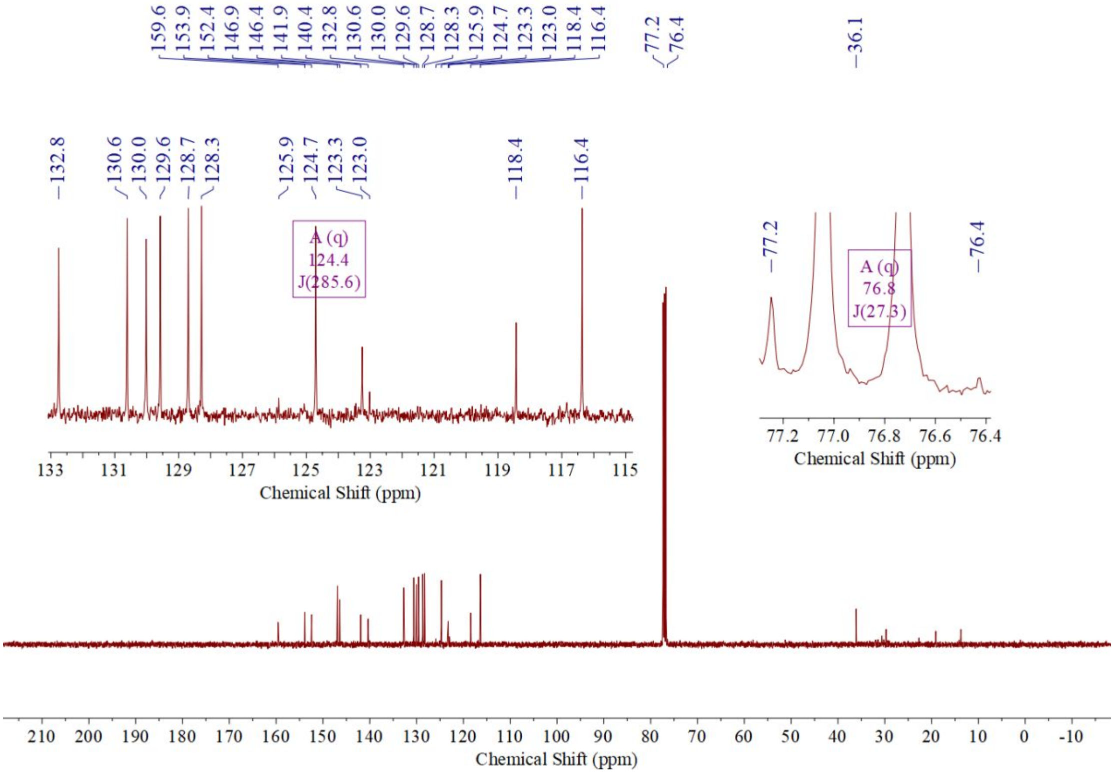
159.6 153.9 152.4 146.9 146.4 141.9 140.4 132.8 130.6 130.0 129.6 128.7 128.3 125.9 124.7 123.3 123.0 118.4 116.4 77.2 76.4 36.1
A (q) 124.4 J(285.6)
Chemical Shift (ppm)
A (q) 76.8 J(27.3)
Chemical Shift (ppm)
Chemical Shift (ppm)

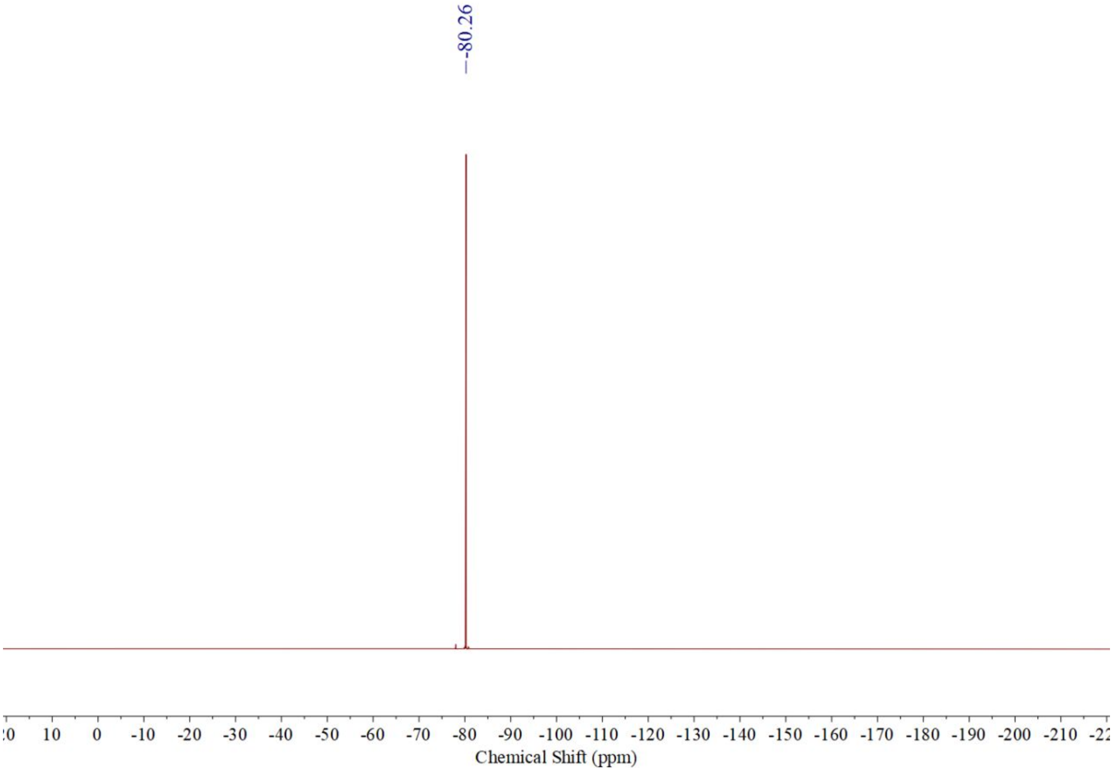
-80.26
Chemical Shift (ppm)

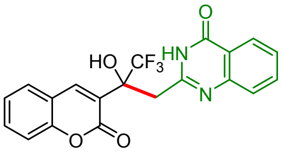
HO
CF3
HN
N
O

(3ap)

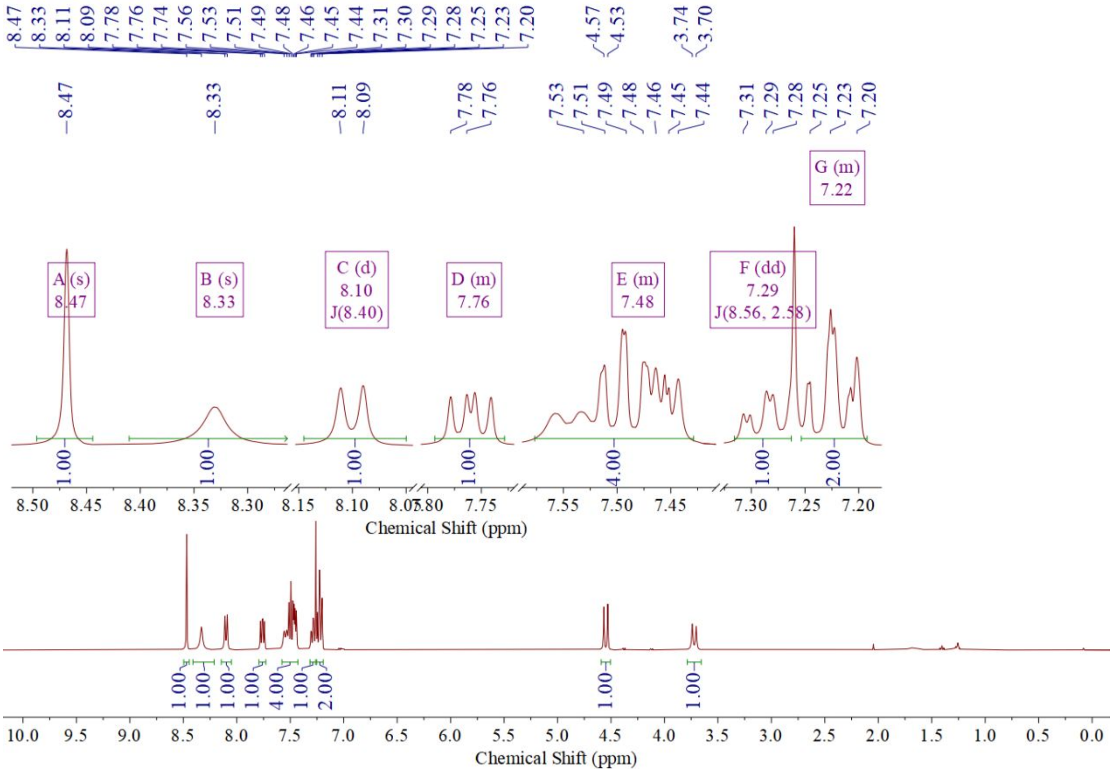
A (s)
8.47
B (s)
8.33
C (d)
8.10
J(8.40)
D (m)
7.76
E (m)
7.48
F (dd)
7.29
J(8.56, 2.58)
G (m)
7.22
Chemical Shift (ppm)
Chemical Shift (ppm)

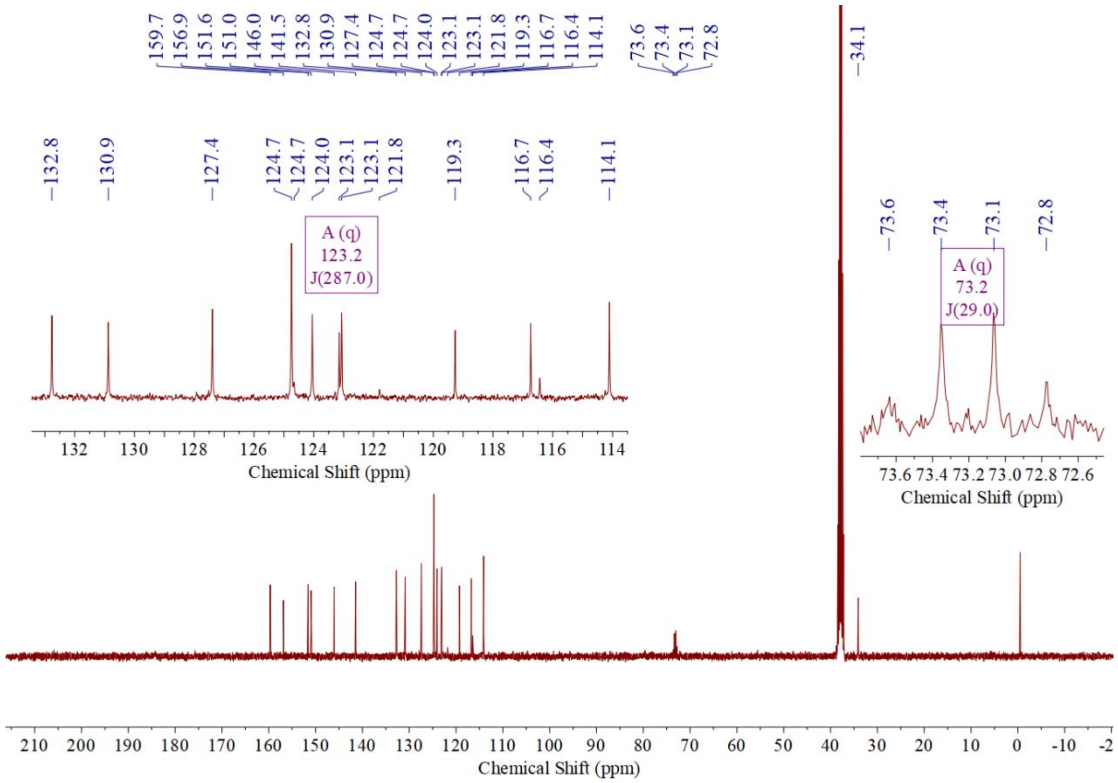
159.7
156.9
151.6
151.0
146.0
141.5
132.8
130.9
127.4
124.7
124.7
124.0
123.1
123.1
121.8
119.3
116.7
116.4
114.1
73.6
73.4
73.1
72.8
34.1
A (q)
123.2
J(287.0)
A (q)
73.2
J(29.0)
Chemical Shift (ppm)
Chemical Shift (ppm)
Chemical Shift (ppm)

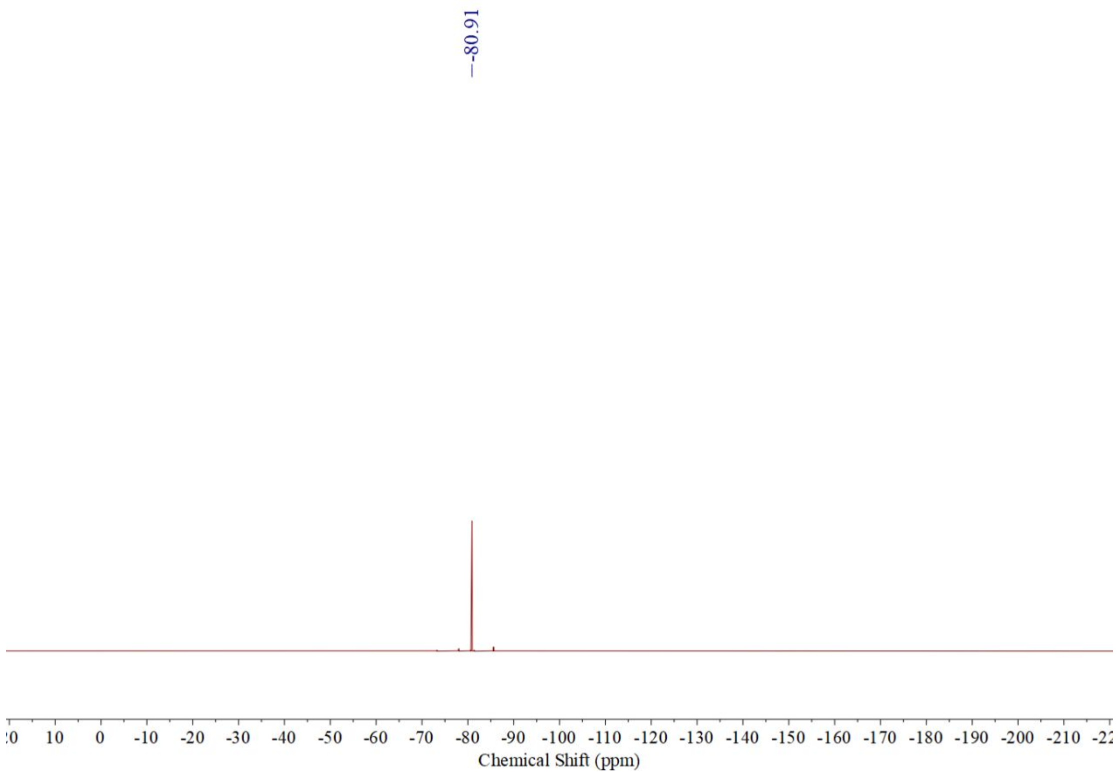
-80.91
Chemical Shift (ppm)

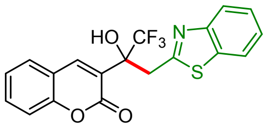
HO
CF3
N
S
O
O

S27

(3aq)

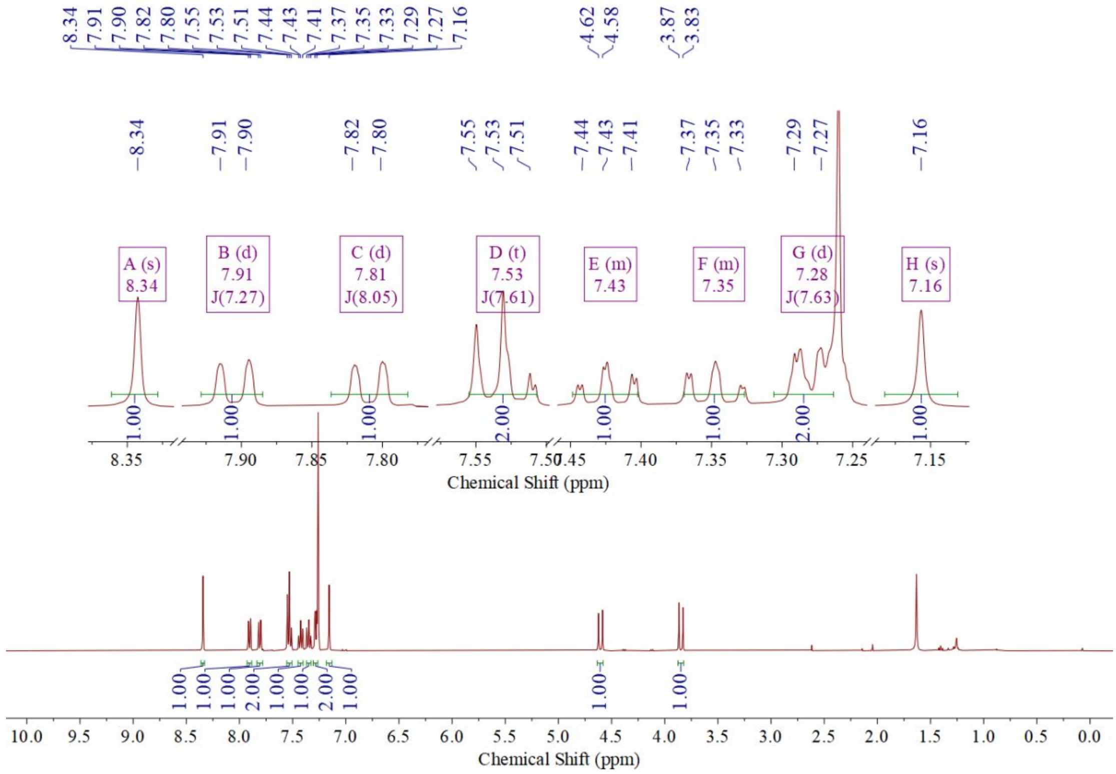

8.34
7.91
7.90
7.82
7.80
7.55
7.53
7.51
7.44
7.43
7.41
7.37
7.35
7.33
7.29
7.27
7.16
4.62
4.58
3.87
3.83
A (s) 8.34
B (d) 7.91 J(7.27)
C (d) 7.81 J(8.05)
D (t) 7.53 J(7.61)
E (m) 7.43
F (m) 7.35
G (d) 7.28 J(7.63)
H (s) 7.16
Chemical Shift (ppm)

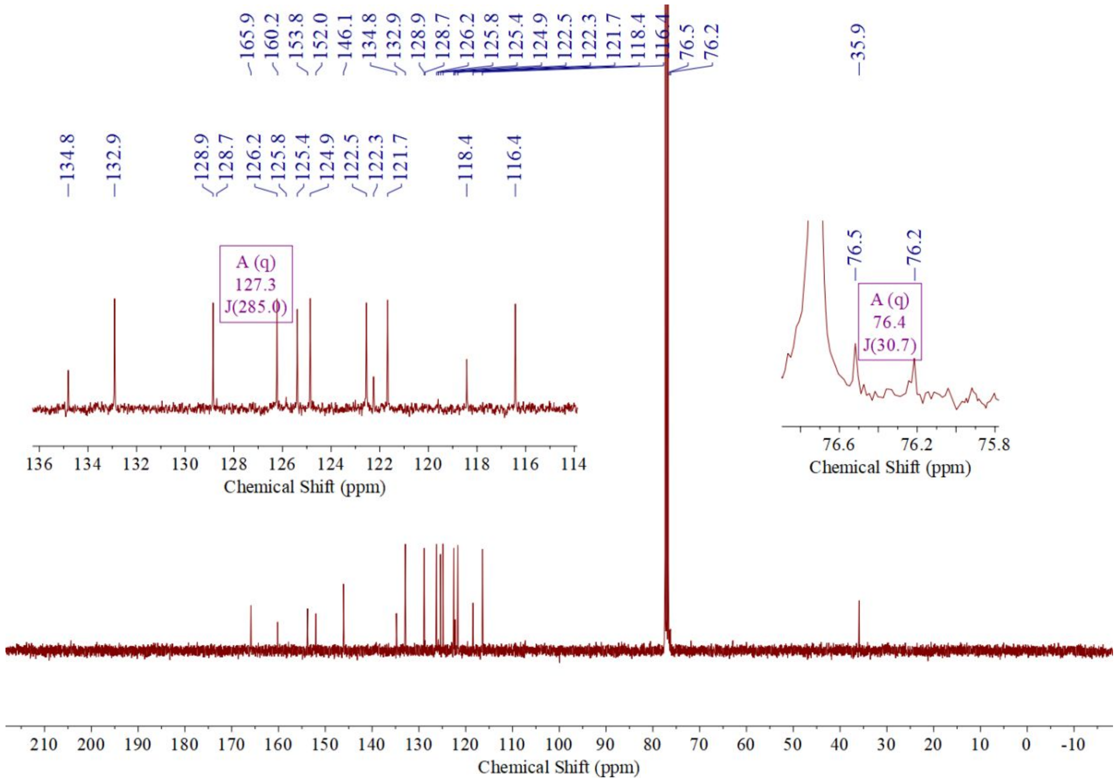

165.9
160.2
153.8
152.0
146.1
134.8
132.9
128.9
128.7
126.2
125.8
125.4
124.9
122.5
122.3
121.7
118.4
116.4
76.5
76.2
35.9
A (q) 127.3 J(285.0)
A (q) 76.4 J(30.7)
Chemical Shift (ppm)

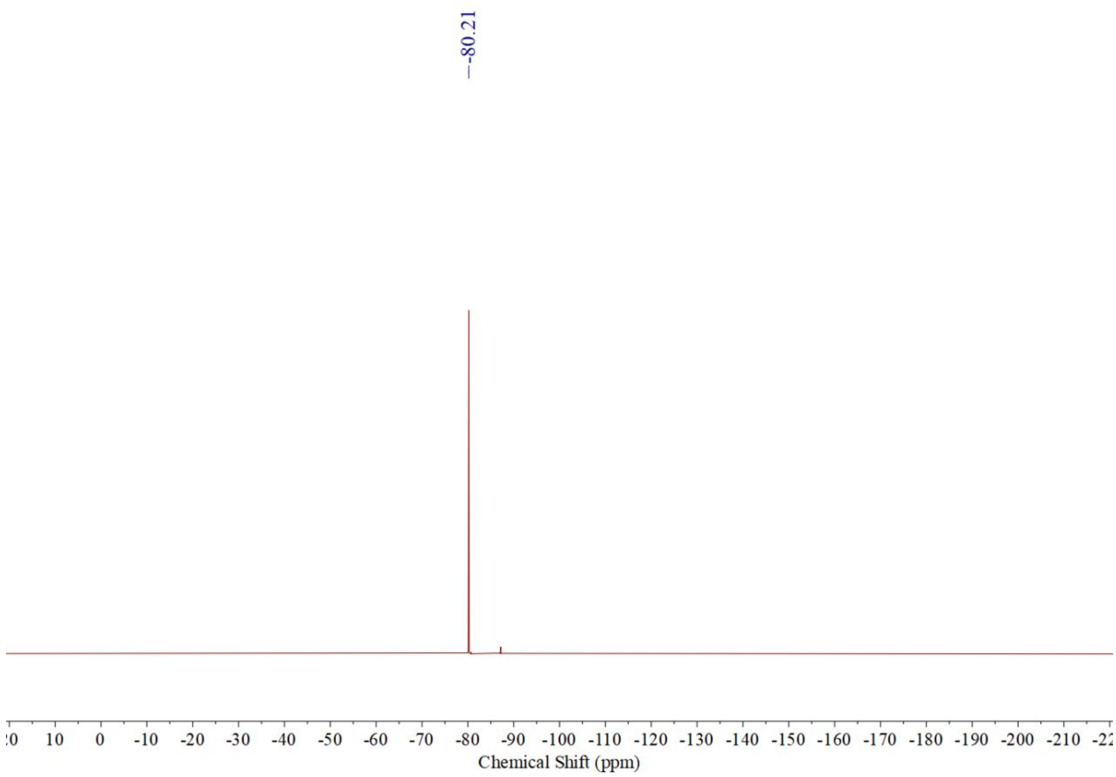
-80.21
Chemical Shift (ppm)

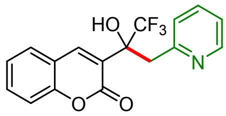

(3ar)

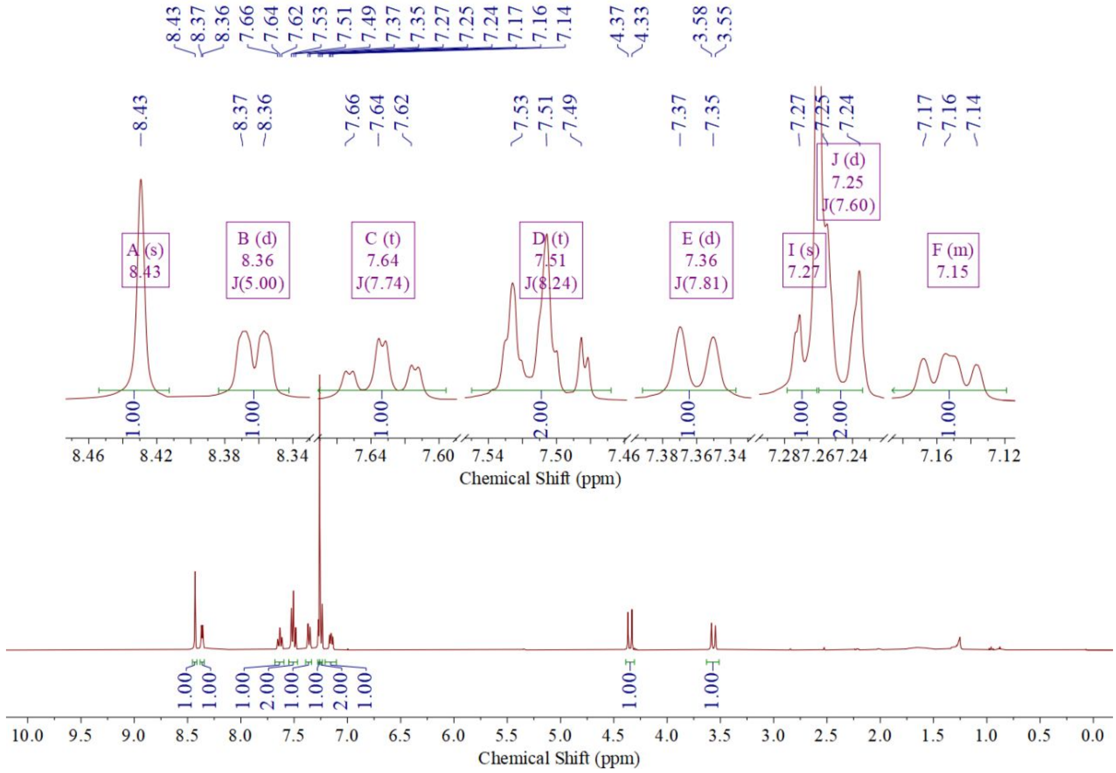
A (s)
8.43
B (d)
8.36
J(5.00)
C (t)
7.64
J(7.74)
D (t)
7.51
J(8.24)
E (d)
7.36
J(7.81)
I (s)
7.27
J (d)
7.25
J(7.60)
F (m)
7.15
Chemical Shift (ppm)

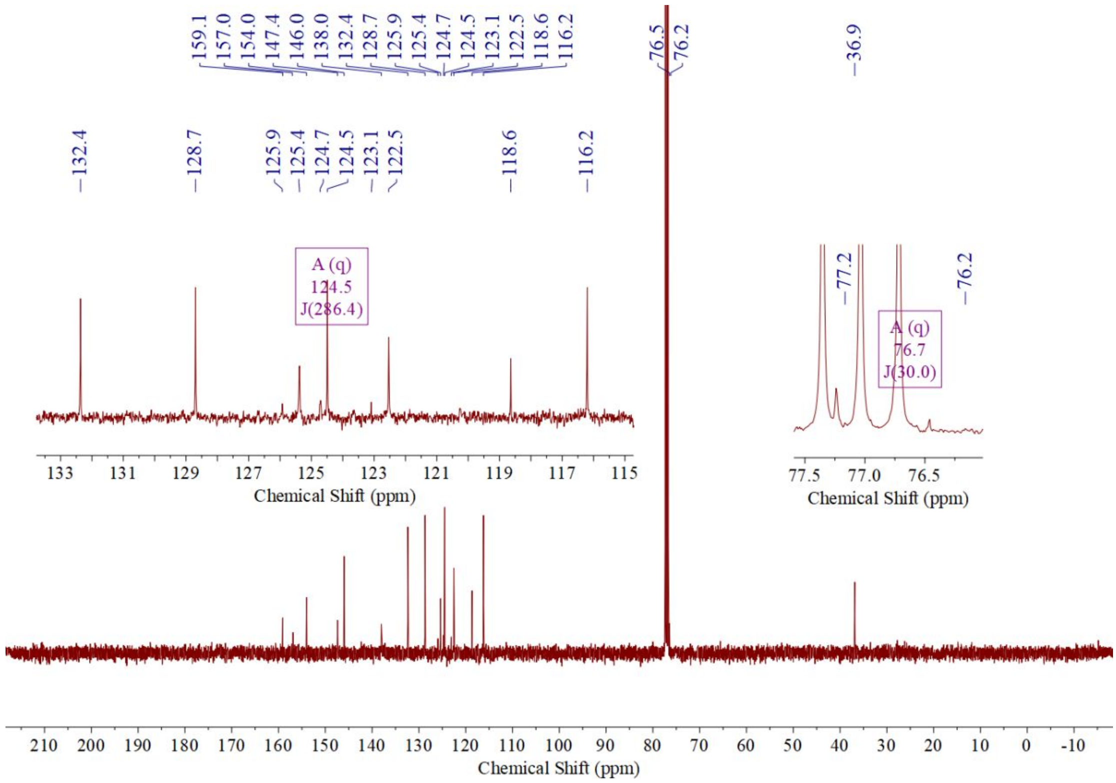
159.1
157.0
154.0
147.4
146.0
138.0
132.4
128.7
125.9
125.4
124.7
124.5
123.1
122.5
118.6
116.2
76.5
76.2
36.9
A (q)
124.5
J(286.4)
A (q)
76.7
J(30.0)
77.2
76.2
Chemical Shift (ppm)
Chemical Shift (ppm)
Chemical Shift (ppm)

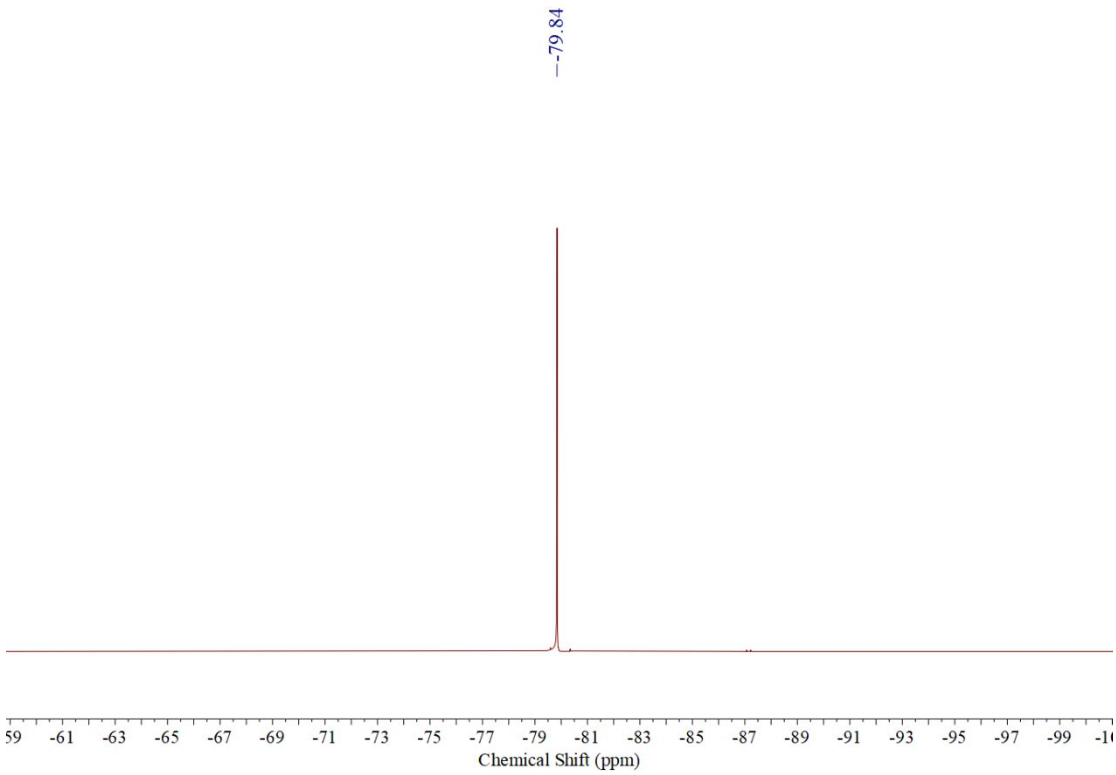
-79.84
Chemical Shift (ppm)

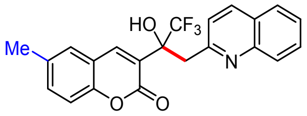
Me
HO
CF3
N
O
O

(3ba)

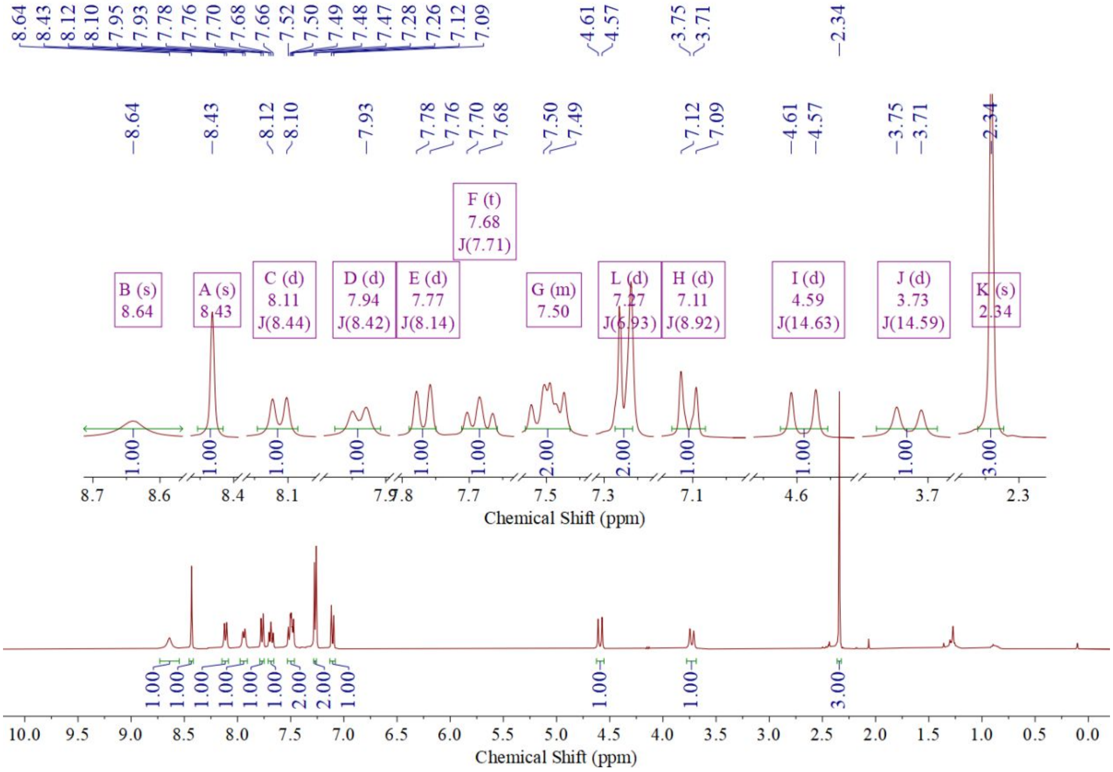
8.64 8.43 8.12 8.10 7.95 7.93 7.78 7.76 7.70 7.68 7.66 7.52 7.50 7.49 7.48 7.47 7.28 7.26 7.12 7.09 4.61 4.57 3.75 3.71 2.34
8.64 8.43 8.12 8.10 7.93 7.78 7.76 7.70 7.68 7.50 7.49 7.12 7.09 4.61 4.57 3.75 3.71 2.34
F (t) 7.68 J(7.71)
B (s) 8.64
A (s) 8.43
C (d) 8.11 J(8.44)
D (d) 7.94 J(8.42)
E (d) 7.77 J(8.14)
G (m) 7.50
L (d) 7.27 J(6.93)
H (d) 7.11 J(8.92)
I (d) 4.59 J(14.63)
J (d) 3.73 J(14.59)
K (s) 2.34
1.00 1.00 1.00 1.00 1.00 1.00 2.00 2.00 1.00 1.00 1.00 3.00
8.7 8.6 8.4 8.1 7.9 7.8 7.7 7.5 7.3 7.1 4.6 3.7 2.3
Chemical Shift (ppm)
1.00 1.00 1.00 1.00 1.00 1.00 2.00 2.00 1.00 1.00 1.00 3.00
10.0 9.5 9.0 8.5 8.0 7.5 7.0 6.5 6.0 5.5 5.0 4.5 4.0 3.5 3.0 2.5 2.0 1.5 1.0 0.5 0.0
Chemical Shift (ppm)

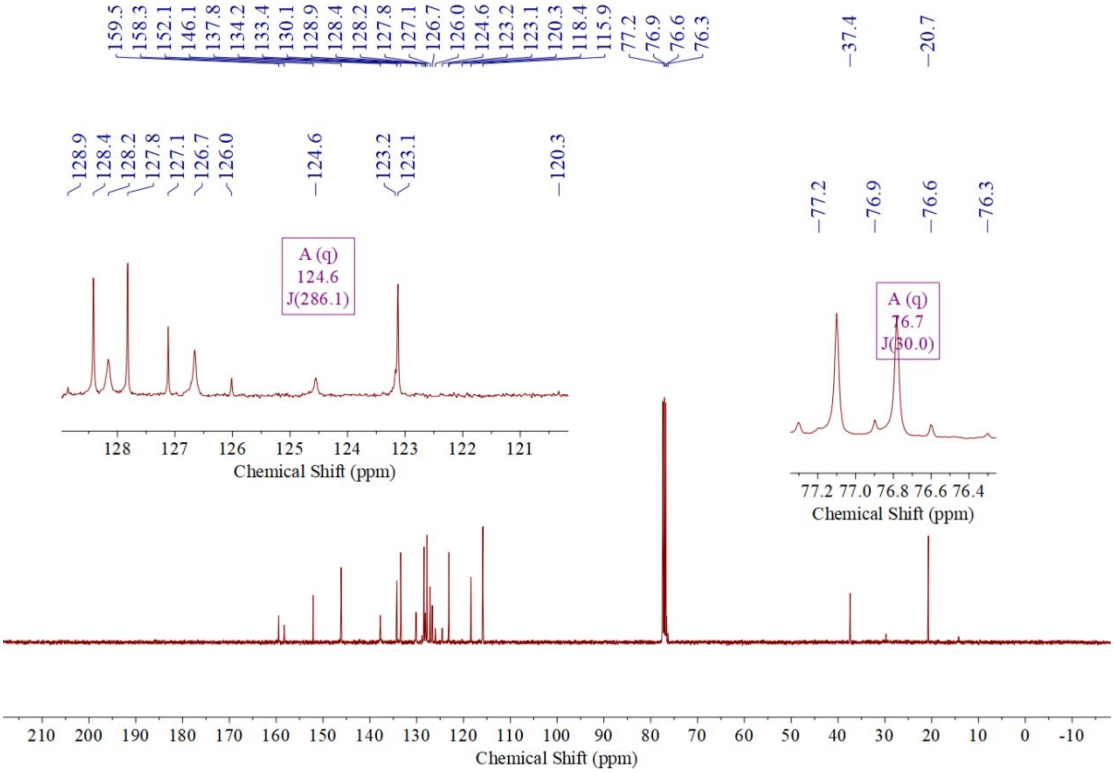
159.5 158.3 152.1 146.1 137.8 134.2 133.4 130.1 128.9 128.4 128.2 127.8 127.1 126.7 126.0 124.6 123.2 123.1 120.3 118.4 115.9 77.2 76.9 76.6 76.3 37.4 20.7
128.9 128.4 128.2 127.8 127.1 126.7 126.0 124.6 123.2 123.1 120.3
77.2 76.9 76.6 76.3
A (q) 124.6 J(286.1)
A (q) 76.7 J(30.0)
128 127 126 125 124 123 122 121
Chemical Shift (ppm)
77.2 77.0 76.8 76.6 76.4
Chemical Shift (ppm)
210 200 190 180 170 160 150 140 130 120 110 100 90 80 70 60 50 40 30 20 10 0 -10
Chemical Shift (ppm)

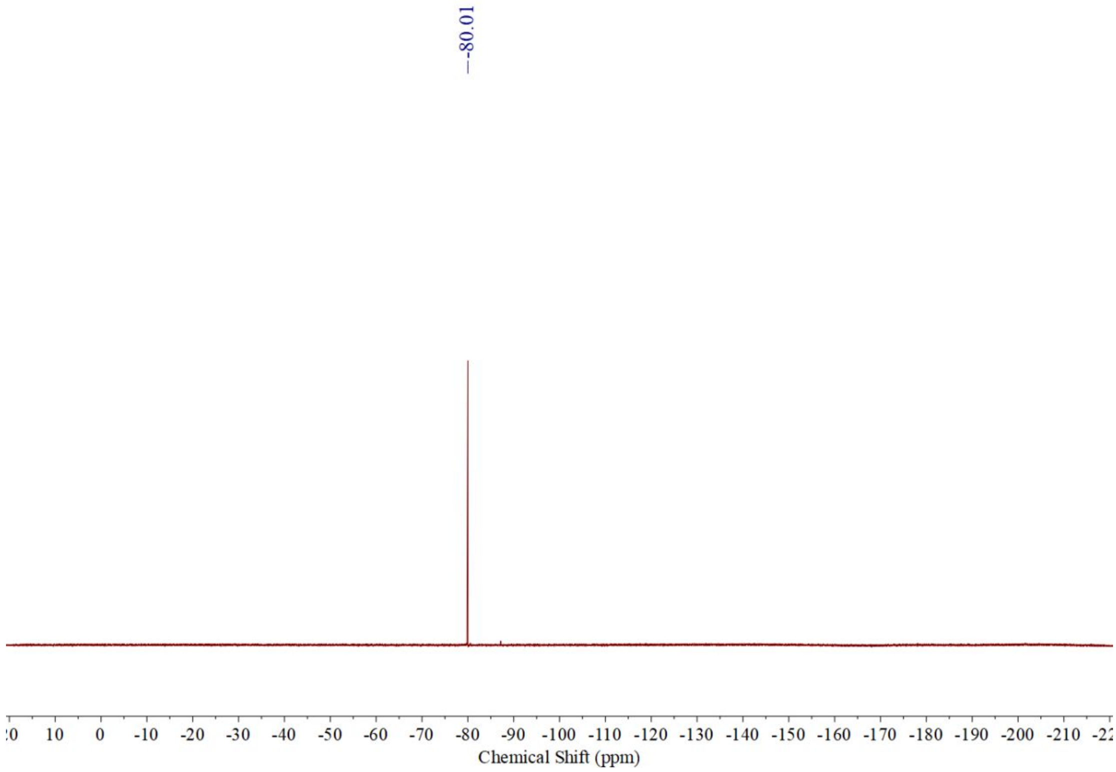

-80.01
Chemical Shift (ppm)

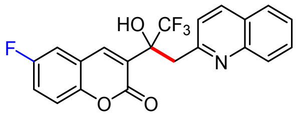

HO
CF3
F
N
O
O

(3ca)

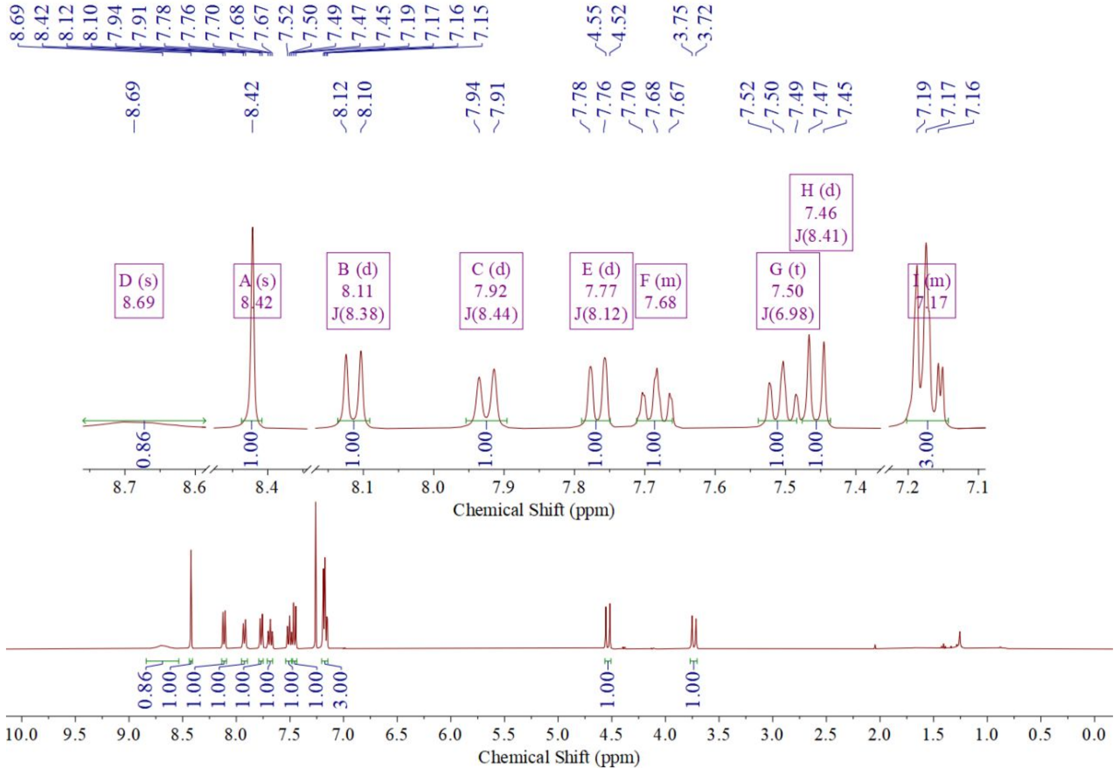

8.69 8.42 8.12 8.10 7.94 7.91 7.78 7.76 7.70 7.68 7.67 7.52 7.50 7.49 7.47 7.45 7.19 7.17 7.16 7.15
4.55 4.52
3.75 3.72
D (s) 8.69
A (s) 8.42
B (d) 8.11 J(8.38)
C (d) 7.92 J(8.44)
E (d) 7.77 J(8.12)
F (m) 7.68
G (t) 7.50 J(6.98)
H (d) 7.46 J(8.41)
I (m) 7.17
Chemical Shift (ppm)
Chemical Shift (ppm)

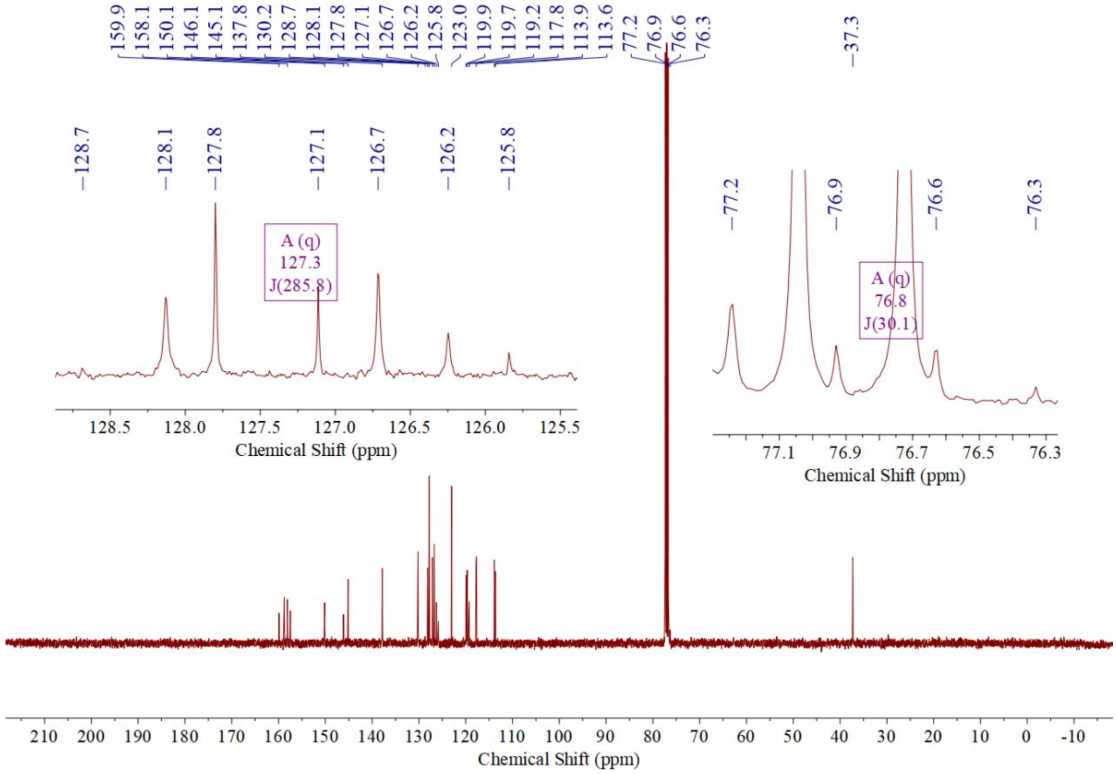
159.9
158.1
150.1
146.1
145.1
137.8
130.2
128.7
128.1
127.8
127.1
126.7
126.2
125.8
123.0
119.9
119.7
119.2
117.8
113.9
113.6
77.2
76.9
76.6
76.3
37.3
A (q)
127.3
J(285.8)
A (q)
76.8
J(30.1)
Chemical Shift (ppm)

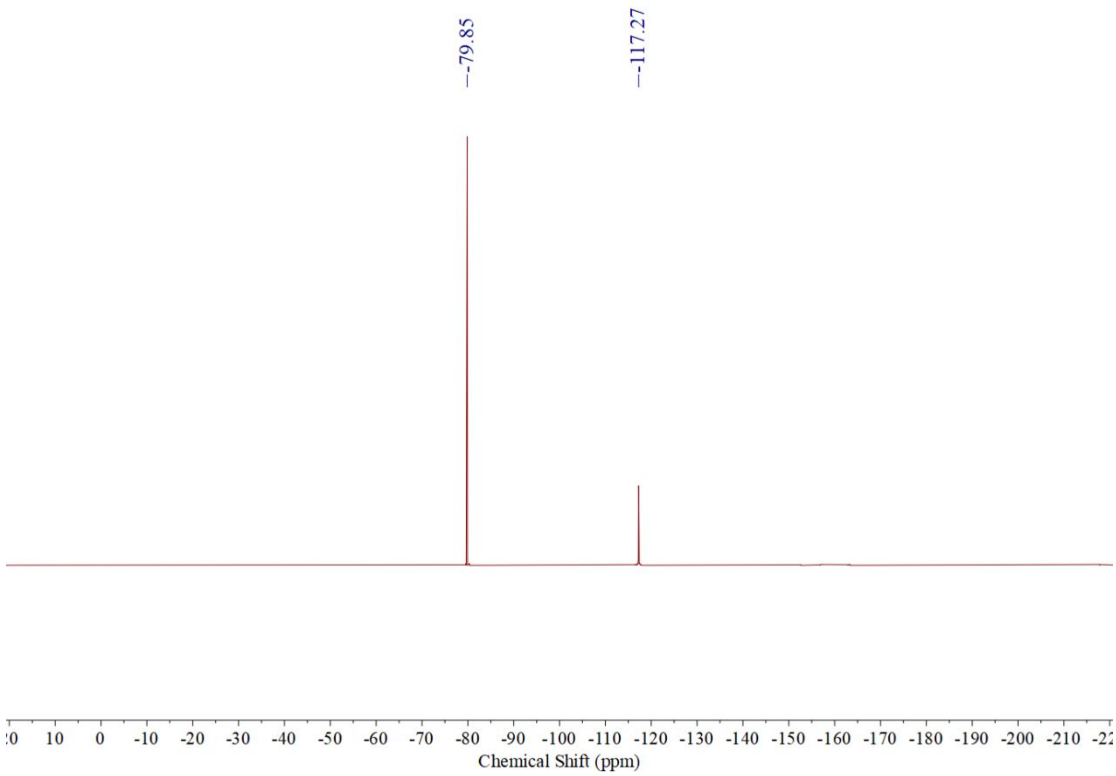
-79.85
-117.27
Chemical Shift (ppm)

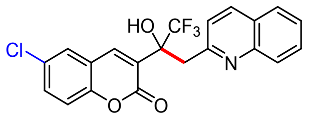

Cl
HO
CF3
N
O
O

(3da)

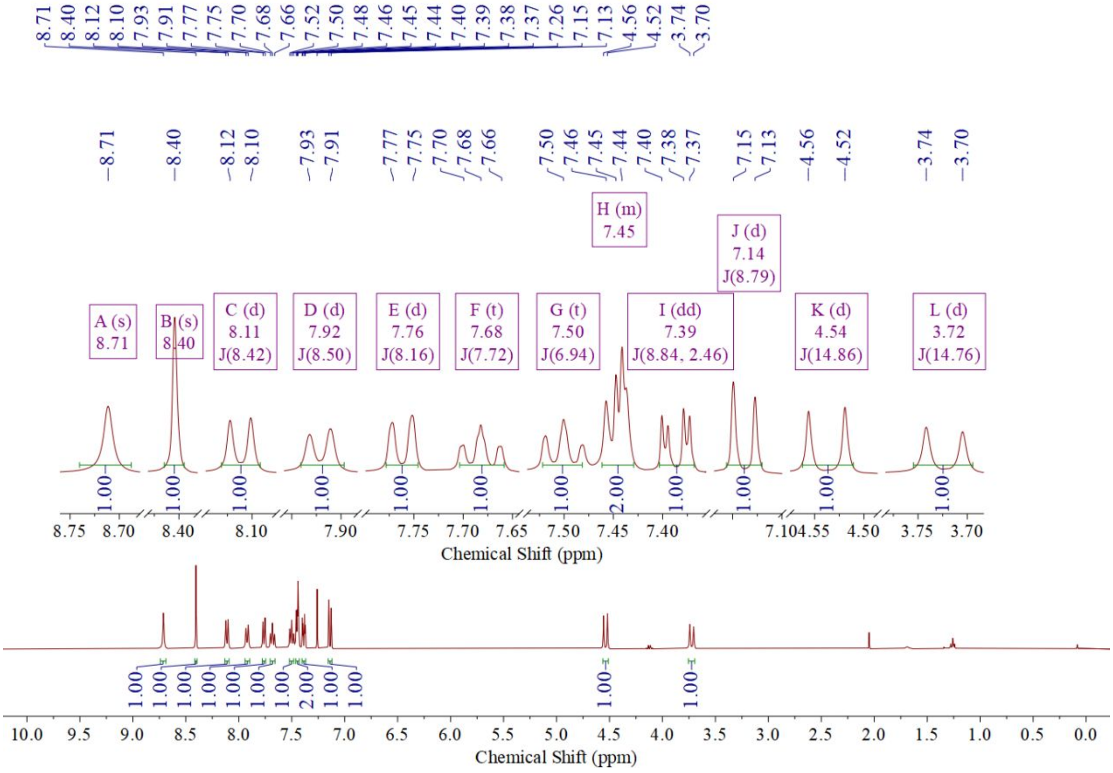

8.71 8.40 8.12 8.10 7.93 7.91 7.77 7.75 7.70 7.68 7.66 7.52 7.50 7.48 7.46 7.45 7.44 7.40 7.39 7.38 7.37 7.26 7.15 7.13 4.56 4.52 3.74 3.70
A (s) 8.71
B (s) 8.40
C (d) 8.11 J(8.42)
D (d) 7.92 J(8.50)
E (d) 7.76 J(8.16)
F (t) 7.68 J(7.72)
G (t) 7.50 J(6.94)
H (m) 7.45
I (dd) 7.39 J(8.84, 2.46)
J (d) 7.14 J(8.79)
K (d) 4.54 J(14.86)
L (d) 3.72 J(14.76)
Chemical Shift (ppm)

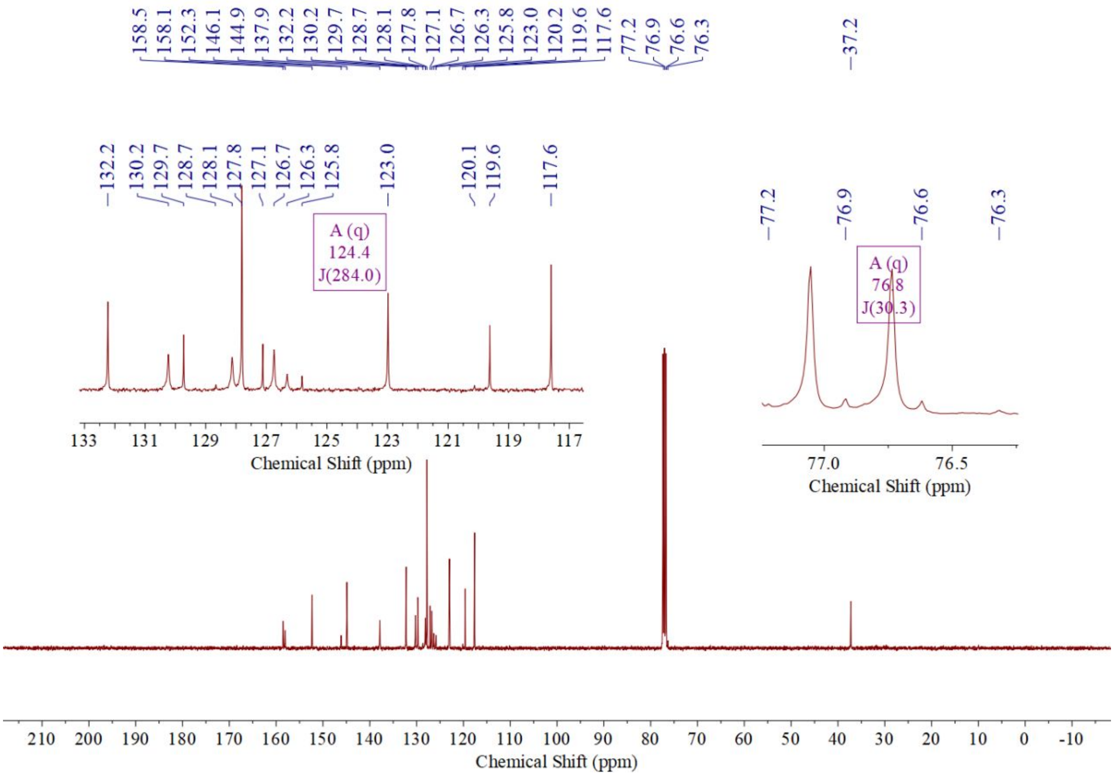

158.5 158.1 152.3 146.1 144.9 137.9 132.2 130.2 129.7 128.7 128.1 127.8 127.1 126.7 126.3 125.8 123.0 120.2 119.6 117.6 77.2 76.9 76.6 76.3 37.2
A (q) 124.4 J(284.0)
A (q) 76.8 J(30.3)
Chemical Shift (ppm)

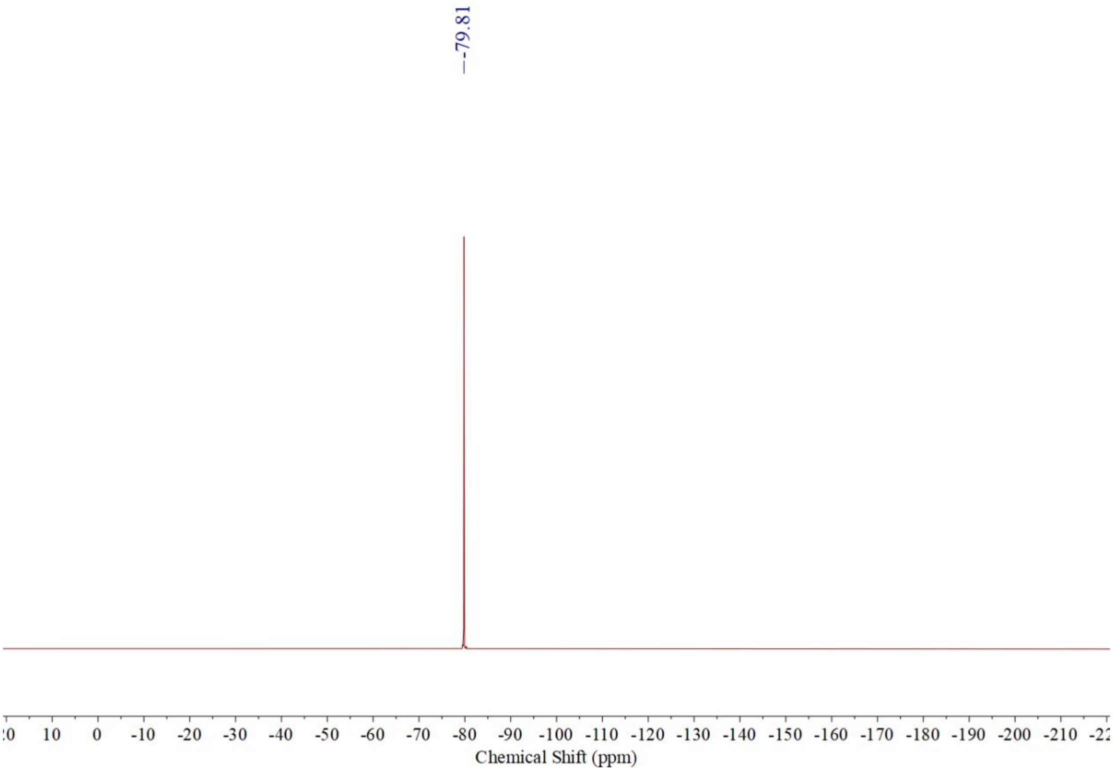
-79.81
Chemical Shift (ppm)

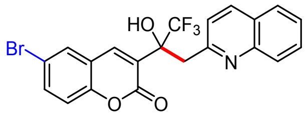
HO
CF3
Br
N
O
O

(3ea)

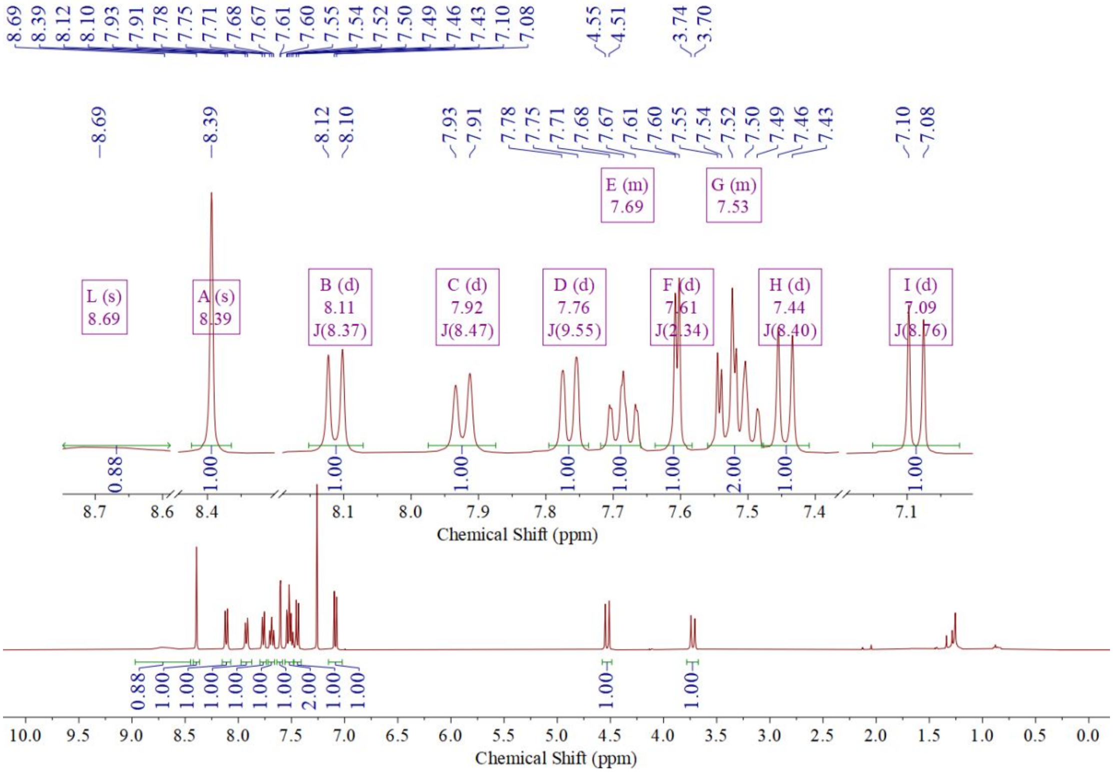
8.69 8.39 8.12 8.10 7.93 7.91 7.78 7.75 7.71 7.68 7.67 7.61 7.60 7.55 7.54 7.52 7.50 7.49 7.46 7.43 7.10 7.08
4.55 4.51
3.74 3.70
E (m) 7.69
G (m) 7.53
L (s) 8.69
A (s) 8.39
B (d) 8.11 J(8.37)
C (d) 7.92 J(8.47)
D (d) 7.76 J(9.55)
F (d) 7.61 J(2.34)
H (d) 7.44 J(8.40)
I (d) 7.09 J(8.76)
0.88 1.00 1.00 1.00 1.00 1.00 1.00 2.00 1.00 1.00
1.00
1.00
Chemical Shift (ppm)

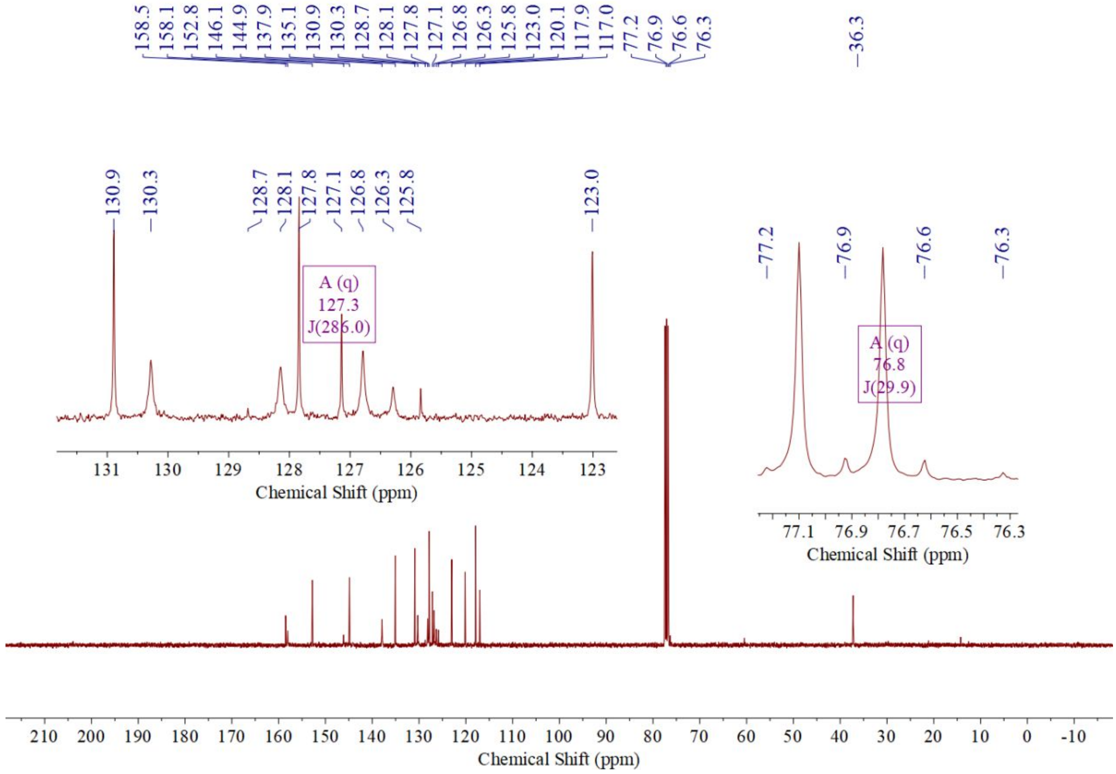

158.5
158.1
152.8
146.1
144.9
137.9
135.1
130.9
130.3
128.7
128.1
127.8
127.1
126.8
126.3
125.8
123.0
120.1
117.9
117.0
77.2
76.9
76.6
76.3
36.3
A (q)
127.3
J(286.0)
A (q)
76.8
J(29.9)
Chemical Shift (ppm)

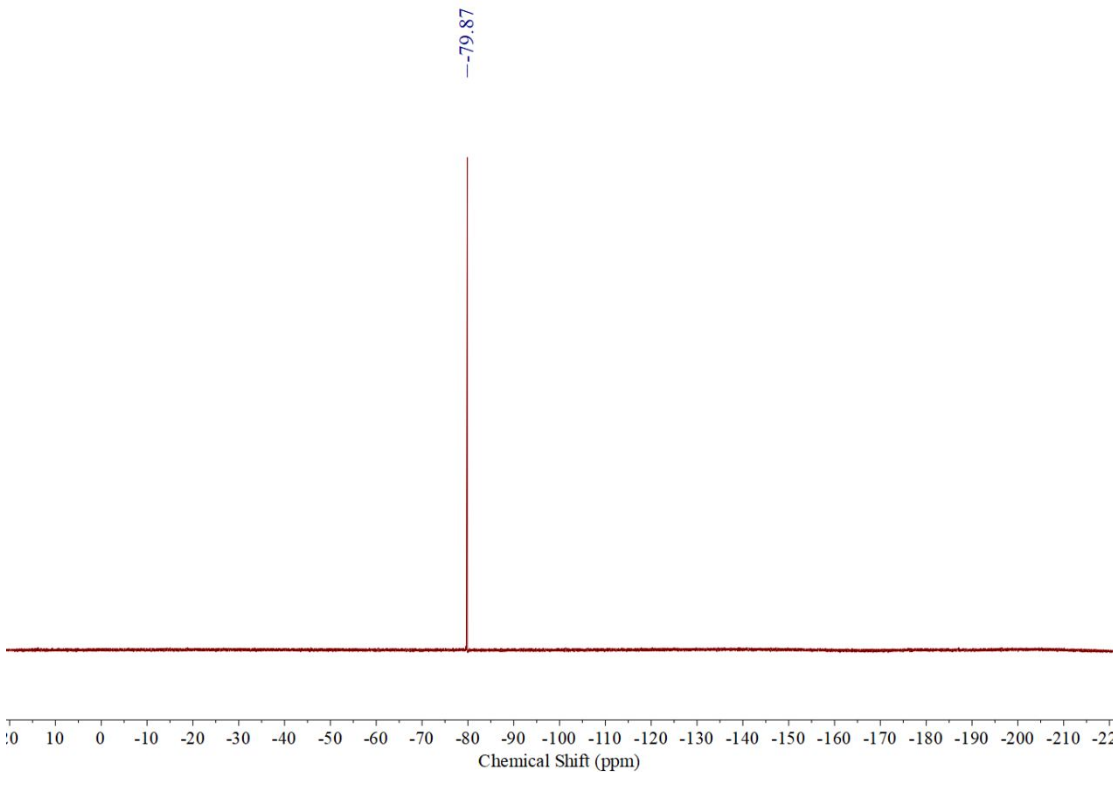

-79.87
Chemical Shift (ppm)

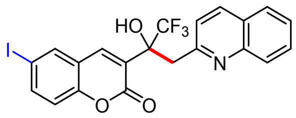

(3fa)

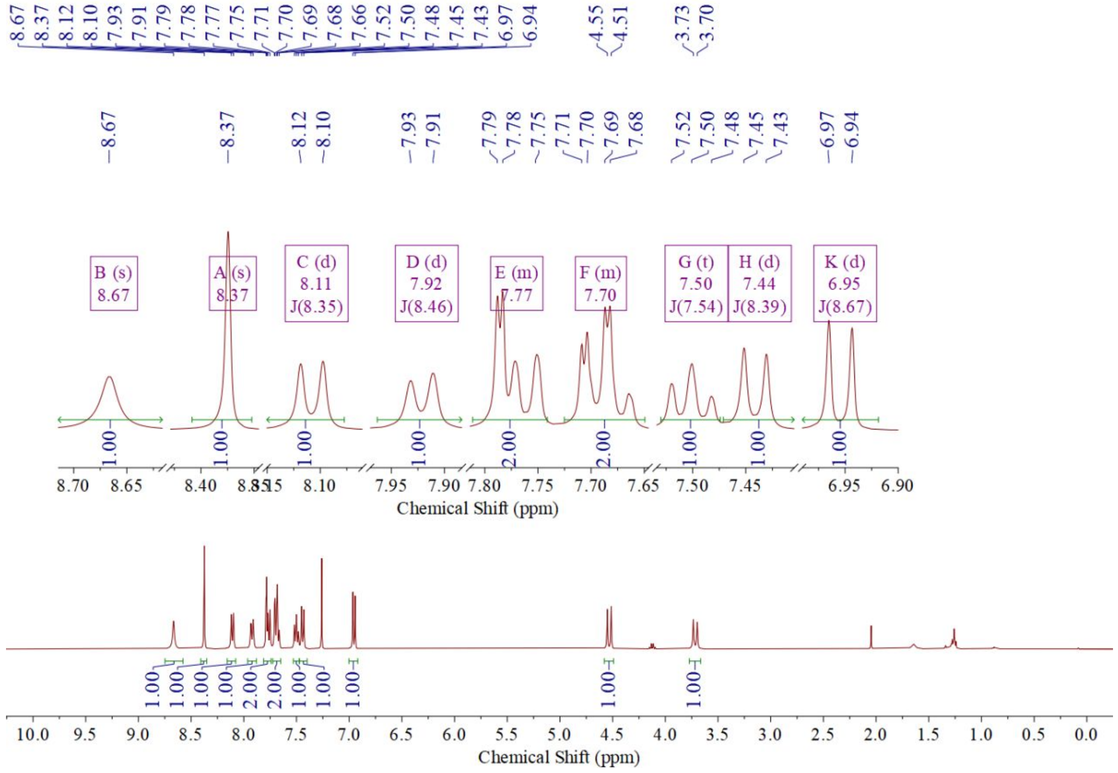

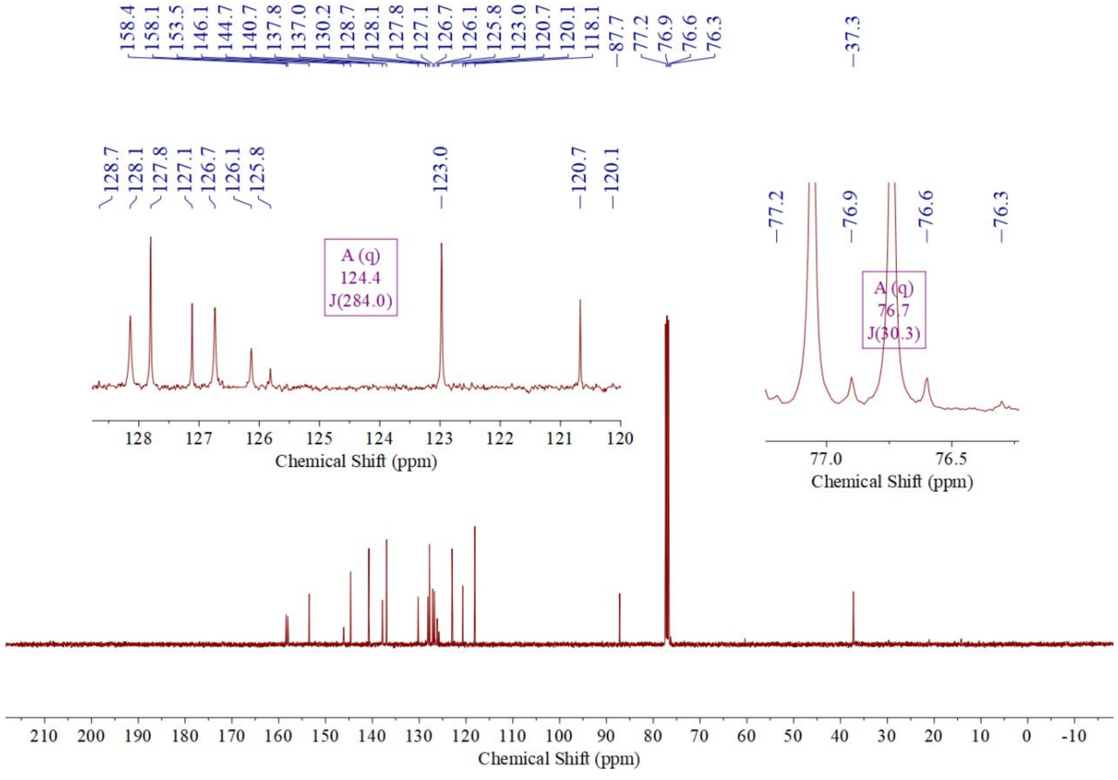

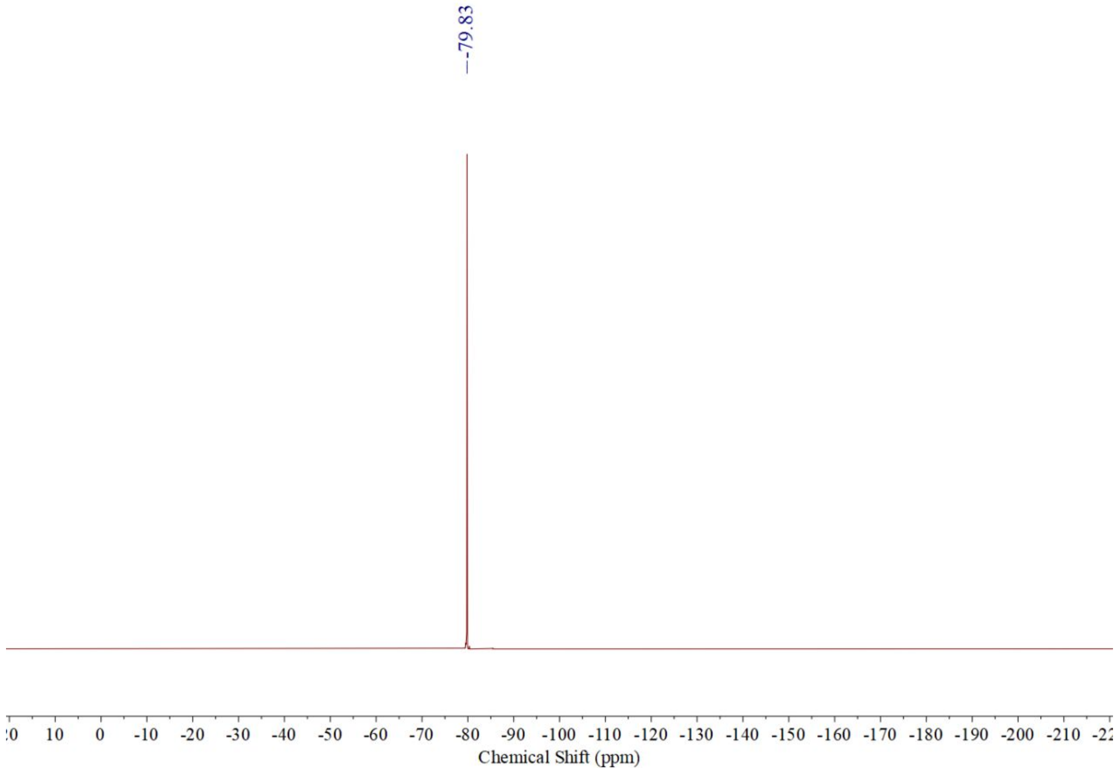
--79.83
Chemical Shift (ppm)

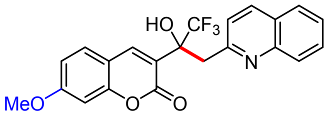
HO
CF3
N
MeO
O
O

(3ga)

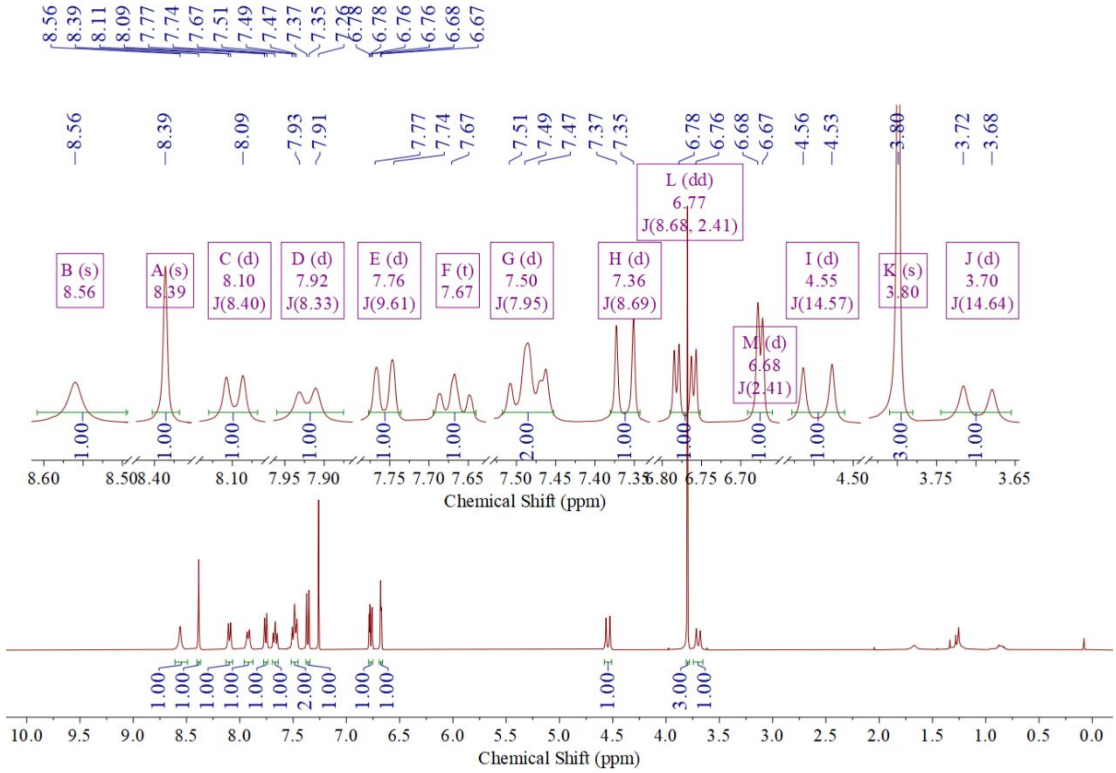
Chemical Shift (ppm)
Chemical Shift (ppm)

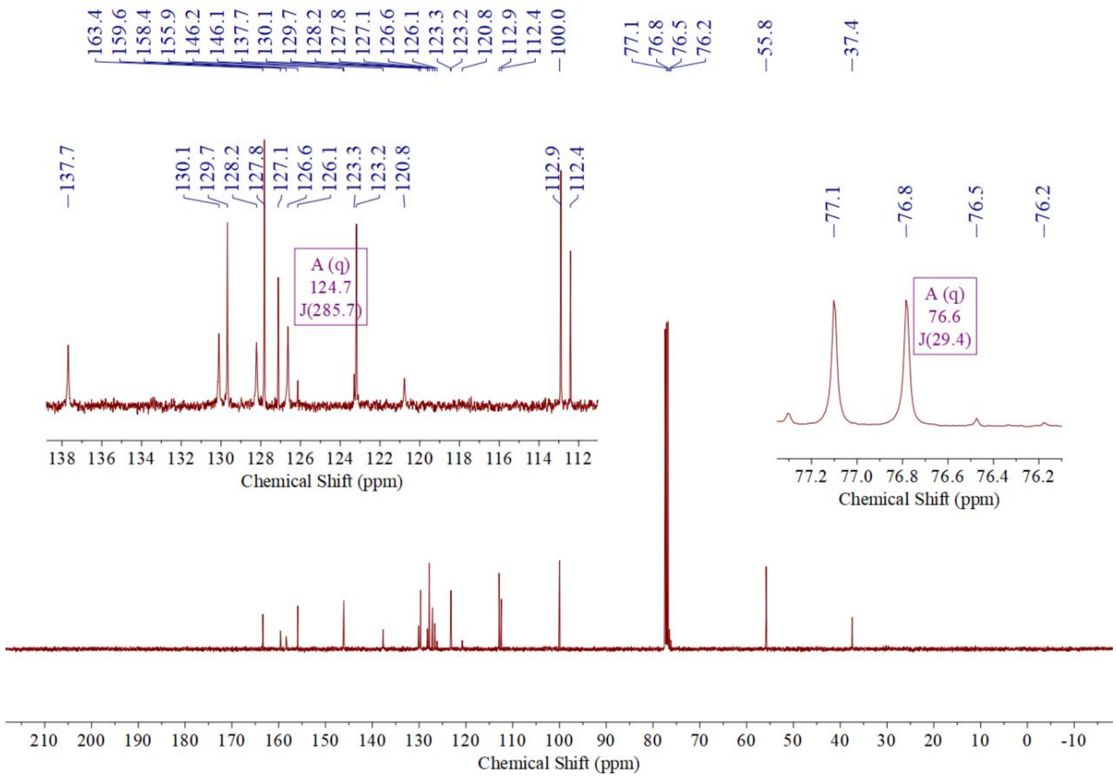

163.4
159.6
158.4
155.9
146.2
146.1
137.7
130.1
129.7
128.2
127.8
127.1
126.6
126.1
123.3
123.2
120.8
112.9
112.4
100.0
77.1
76.8
76.5
76.2
55.8
37.4
A (q)
124.7
J(285.7)
A (q)
76.6
J(29.4)
Chemical Shift (ppm)

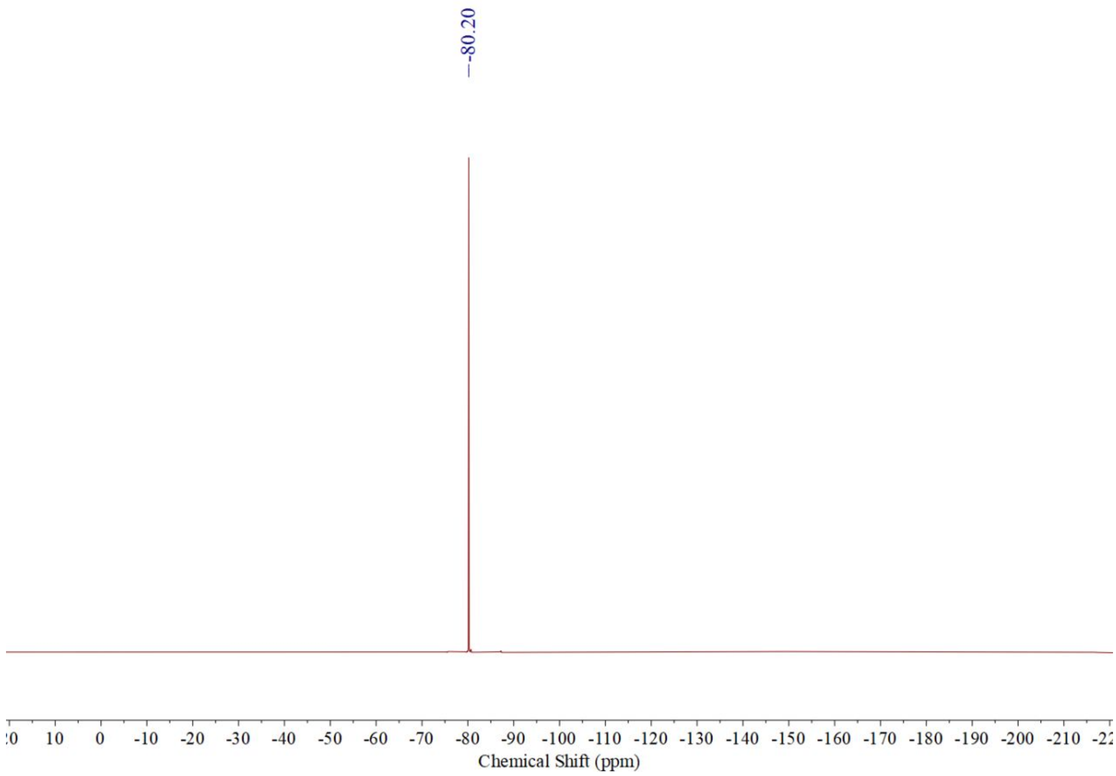

-80.20
Chemical Shift (ppm)

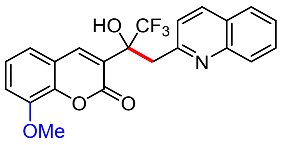
HO
CF3
N
O
O
OMe

(3ha)

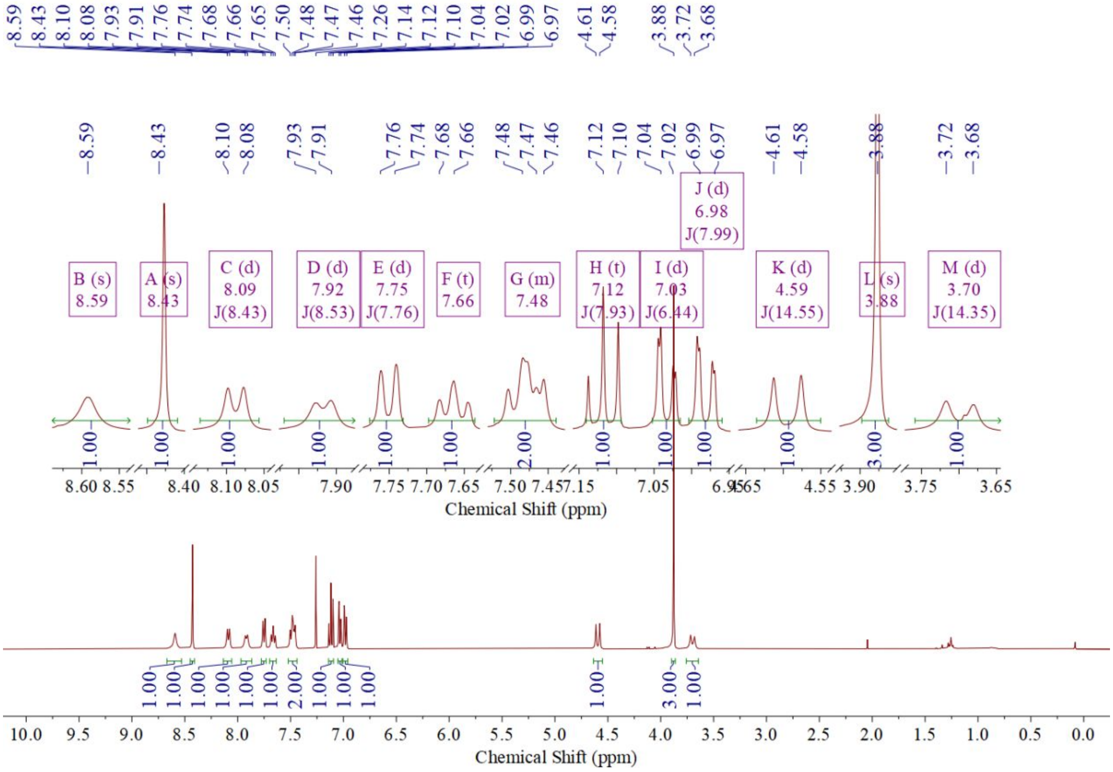
8.59 8.43 8.10 8.08 7.93 7.91 7.76 7.74 7.68 7.66 7.65 7.50 7.48 7.47 7.46 7.26 7.14 7.12 7.10 7.04 7.02 6.99 6.97 4.61 4.58 3.88 3.72 3.68
B (s) 8.59
A (s) 8.43
C (d) 8.09 J(8.43)
D (d) 7.92 J(8.53)
E (d) 7.75 J(7.76)
F (t) 7.66
G (m) 7.48
H (t) 7.12 J(7.93)
I (d) 7.03 J(6.44)
J (d) 6.98 J(7.99)
K (d) 4.59 J(14.55)
L (s) 3.88
M (d) 3.70 J(14.35)
Chemical Shift (ppm)
Chemical Shift (ppm)

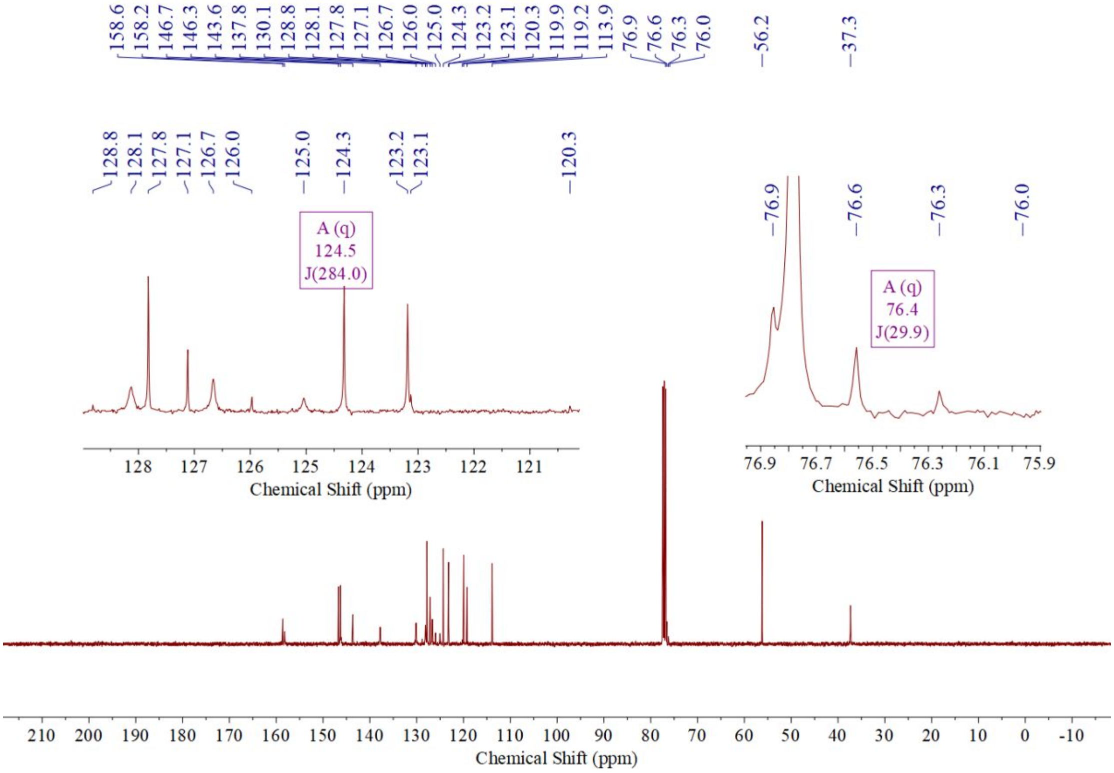
158.6 158.2 146.7 146.3 143.6 137.8 130.1 128.8 128.1 127.8 127.1 126.7 126.0 125.0 124.3 123.2 123.1 120.3 119.9 119.2 113.9 76.9 76.6 76.3 76.0 56.2 37.3
A (q) 124.5 J(284.0)
A (q) 76.4 J(29.9)
Chemical Shift (ppm)
Chemical Shift (ppm)
Chemical Shift (ppm)

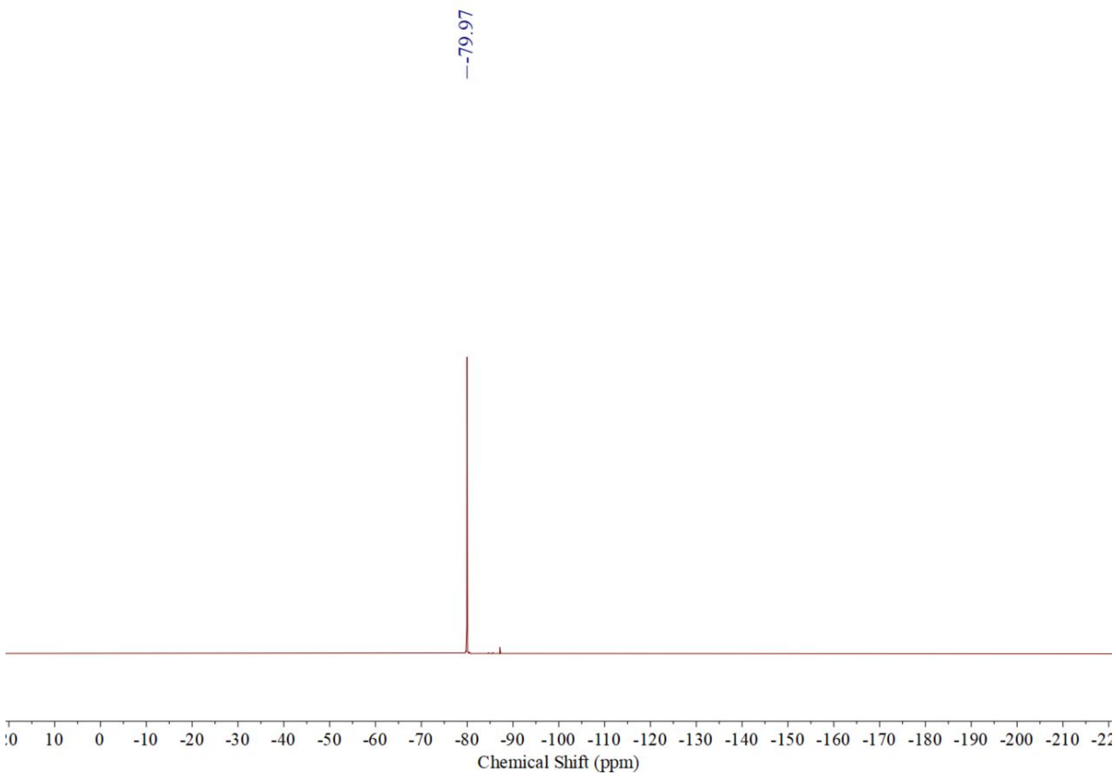
-79.97
Chemical Shift (ppm)

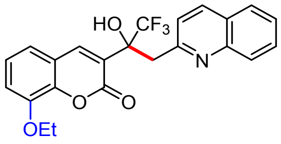
HO
CF3
N
O
O
OEt

(3ia)

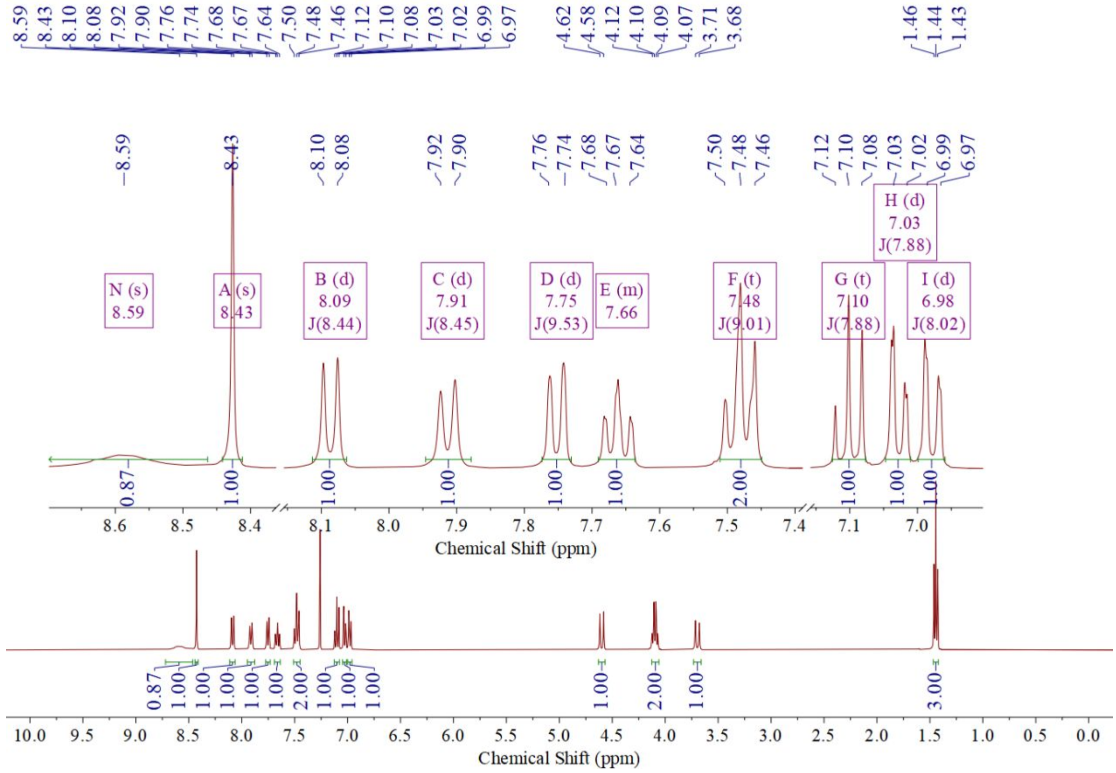
8.59 8.43 8.10 8.08 7.92 7.90 7.76 7.74 7.68 7.67 7.64 7.50 7.48 7.46 7.12 7.10 7.08 7.03 7.02 6.99 6.97
4.62 4.58 4.12 4.10 4.09 4.07 3.71 3.68
1.46 1.44 1.43
N (s) 8.59
A (s) 8.43
B (d) 8.09 J(8.44)
C (d) 7.91 J(8.45)
D (d) 7.75 J(9.53)
E (m) 7.66
F (t) 7.48 J(9.01)
G (t) 7.10 J(7.88)
H (d) 7.03 J(7.88)
I (d) 6.98 J(8.02)
0.87 1.00 1.00 1.00 1.00 1.00 2.00 1.00 1.00 1.00
1.00 2.00 1.00 3.00
Chemical Shift (ppm)

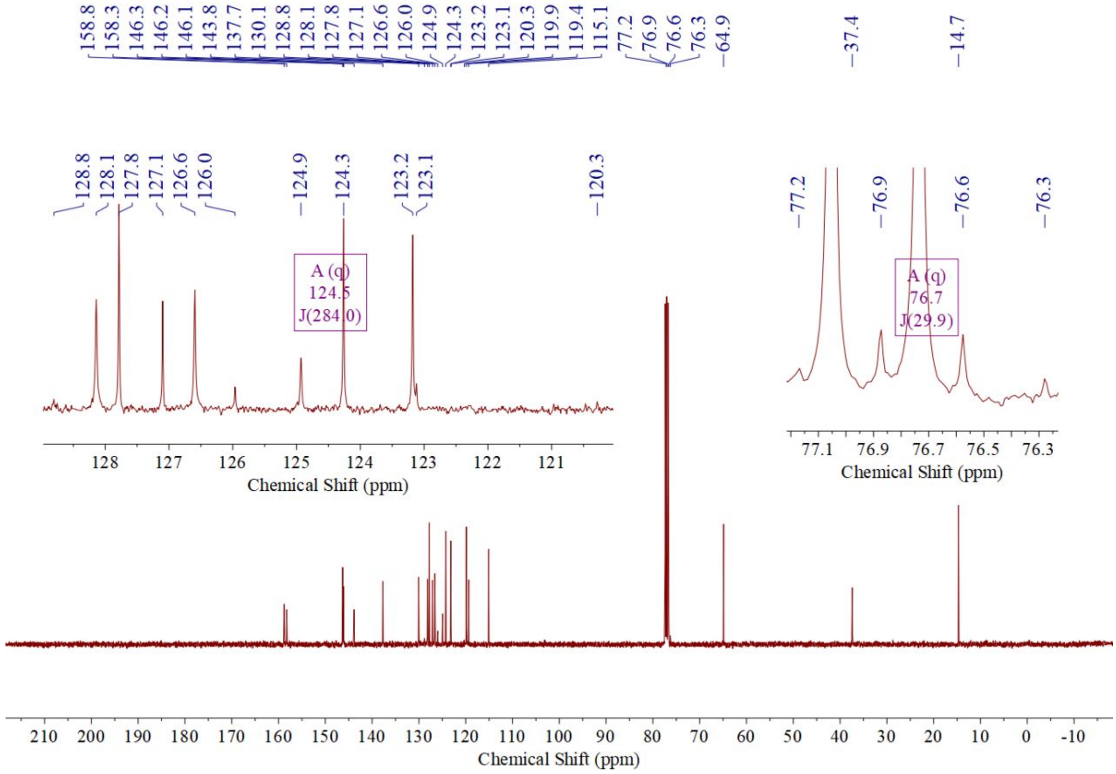
158.8
158.3
146.3
146.2
146.1
143.8
137.7
130.1
128.8
128.1
127.8
127.1
126.6
126.0
124.9
124.3
123.2
123.1
120.3
119.9
119.4
115.1
77.2
76.9
76.6
76.3
64.9
37.4
14.7
A (q)
124.5
J(284.0)
A (q)
76.7
J(29.9)
Chemical Shift (ppm)

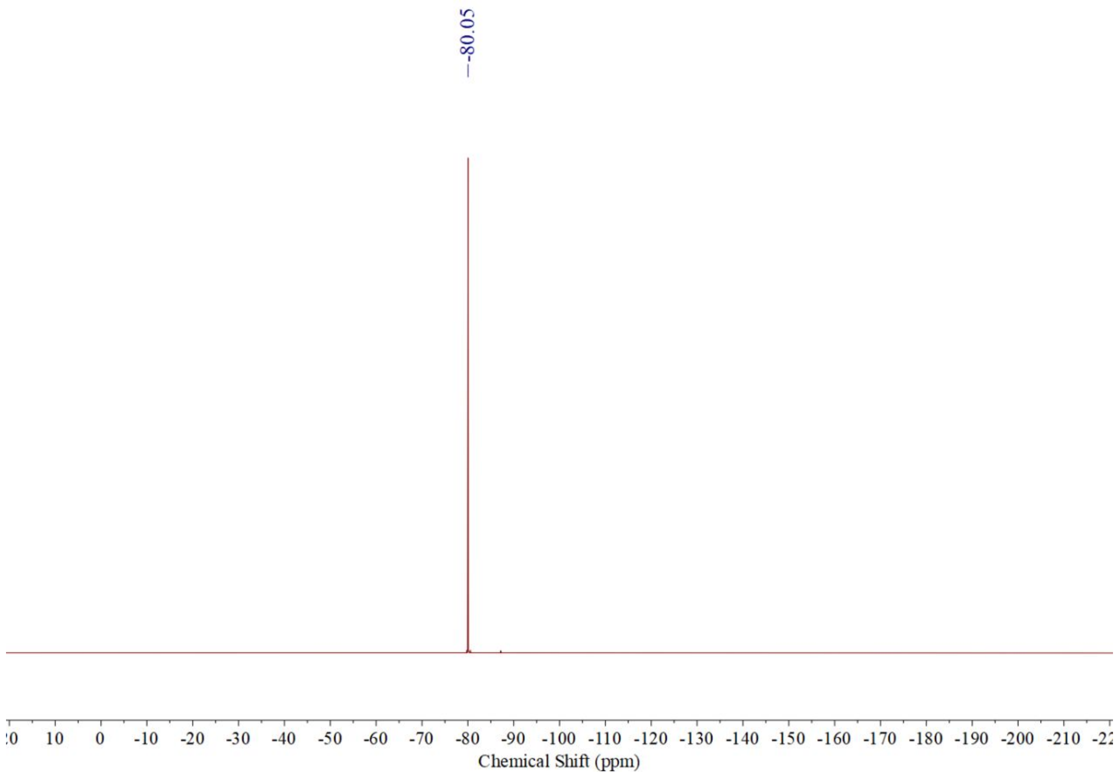
-80.05
Chemical Shift (ppm)

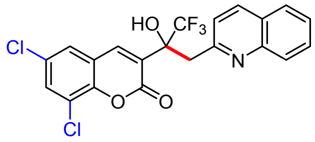

(3ja)

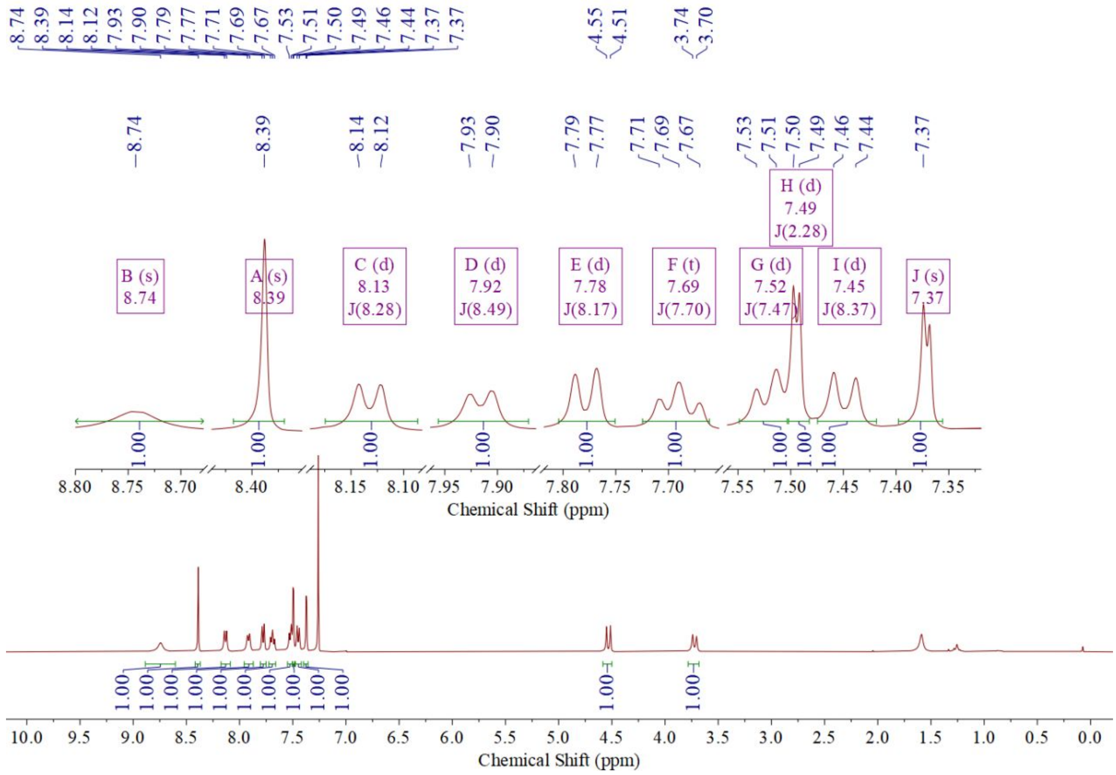

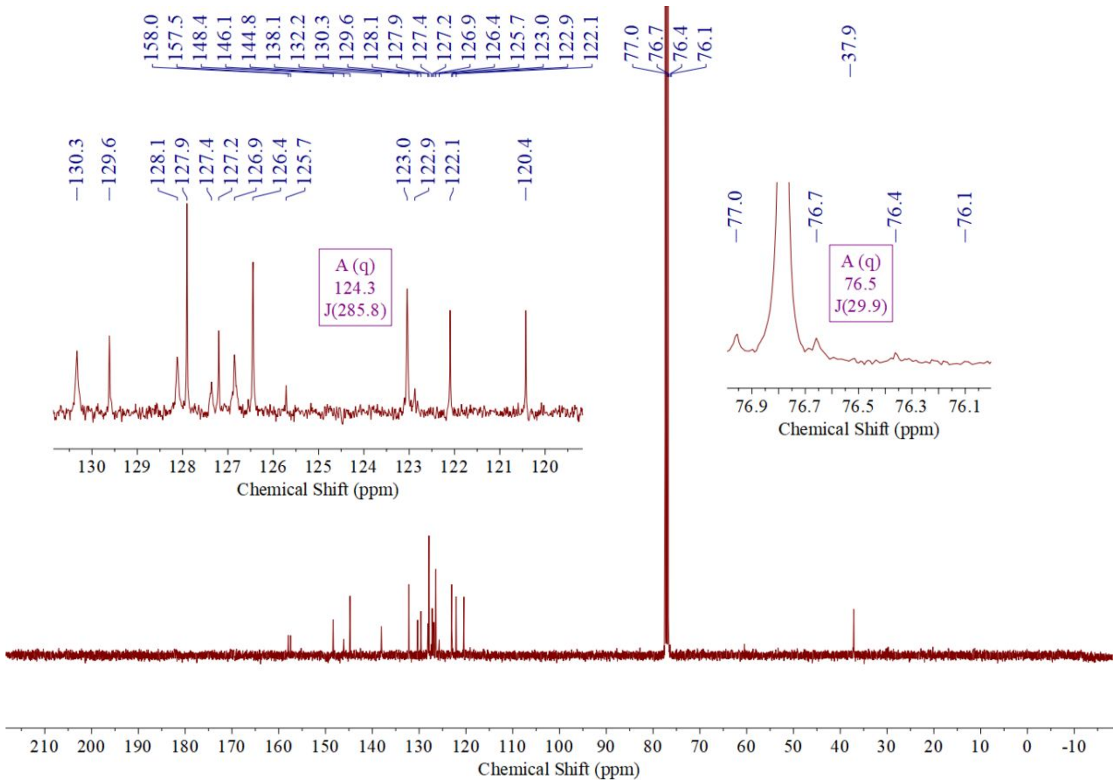

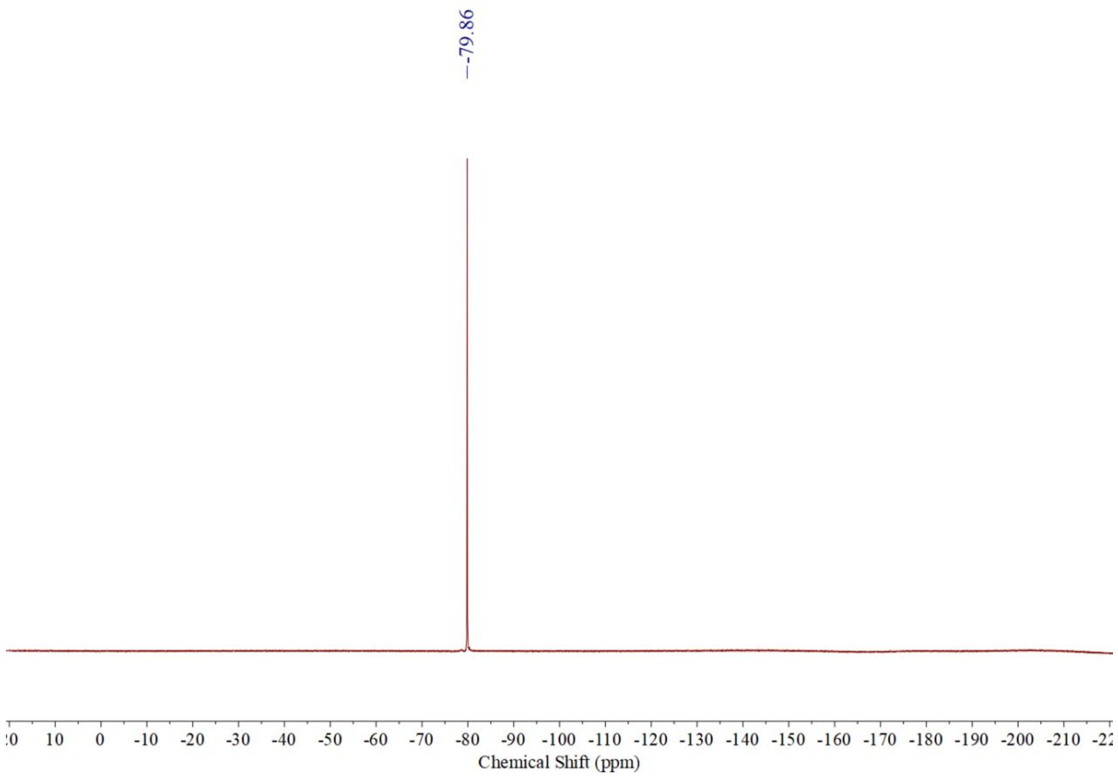
-79.86
Chemical Shift (ppm)

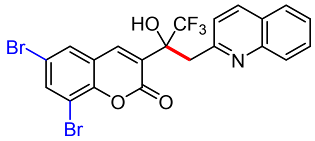
Br
HO
CF3
N
O
O
Br

(3ka)

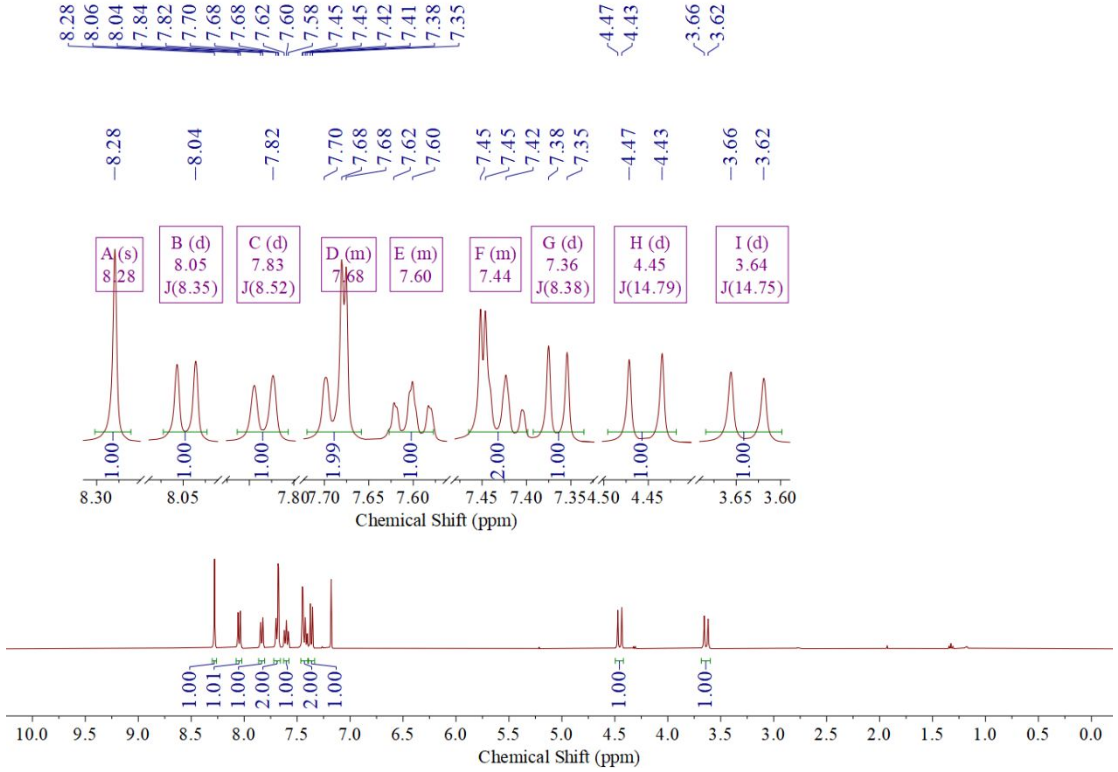
A (s) 8.28
B (d) 8.05 J(8.35)
C (d) 7.83 J(8.52)
D (m) 7.68
E (m) 7.60
F (m) 7.44
G (d) 7.36 J(8.38)
H (d) 4.45 J(14.79)
I (d) 3.64 J(14.75)
Chemical Shift (ppm)
Chemical Shift (ppm)

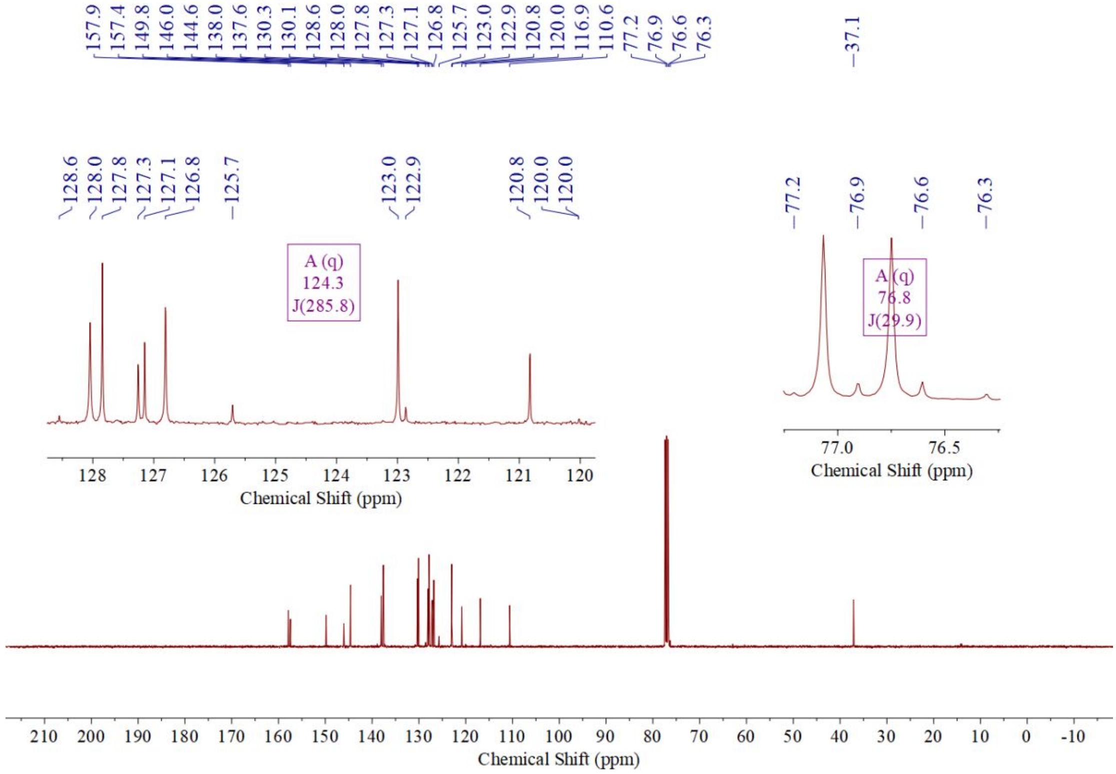
157.9
157.4
149.8
146.0
144.6
138.0
137.6
130.3
130.1
128.6
128.0
127.8
127.3
127.1
126.8
125.7
123.0
122.9
120.8
120.0
116.9
110.6
77.2
76.9
76.6
76.3
37.1
A (q)
124.3
J(285.8)
A (q)
76.8
J(29.9)
Chemical Shift (ppm)
Chemical Shift (ppm)
Chemical Shift (ppm)

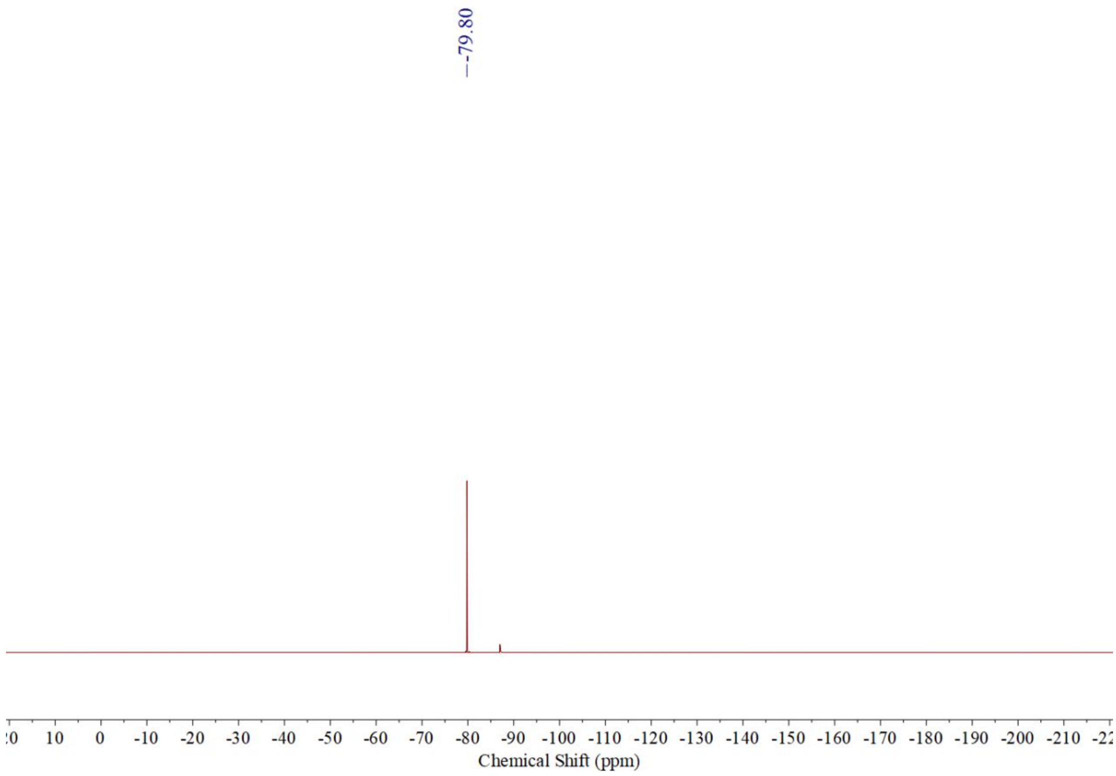
-79.80
Chemical Shift (ppm)

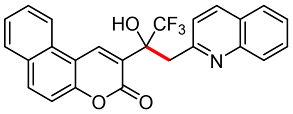
HO
CF3
N
O
O

(3la)

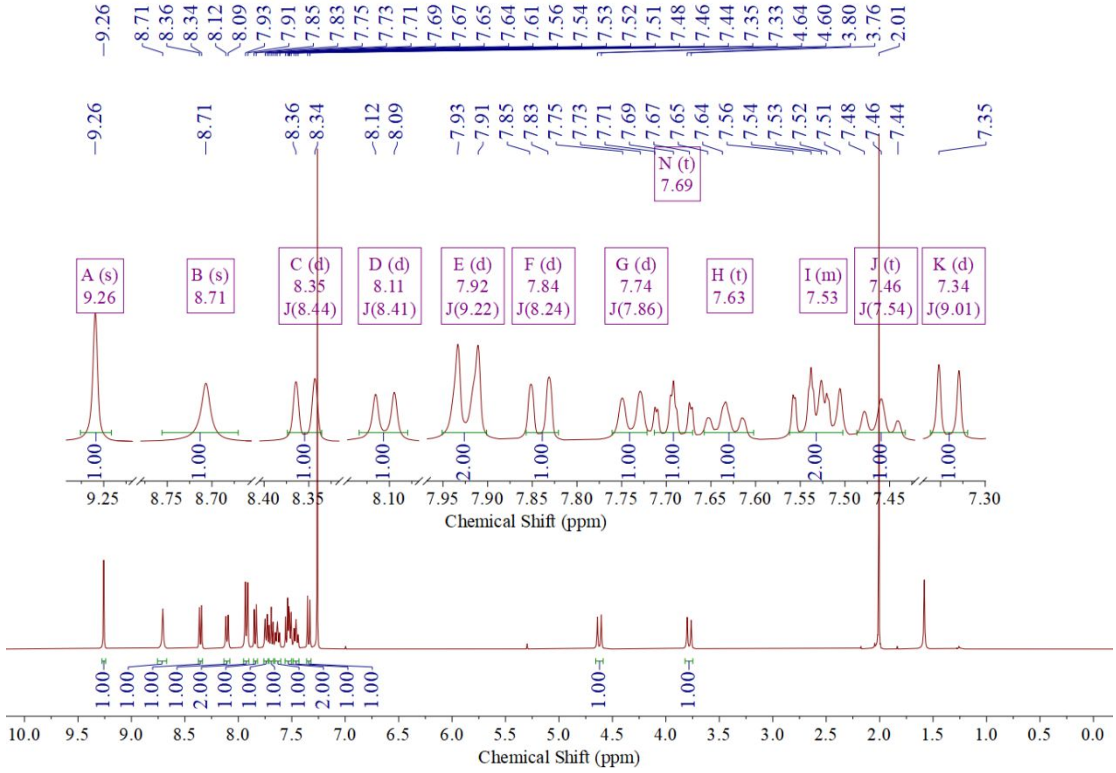
9.26
8.71
8.36
8.34
8.12
8.09
7.93
7.91
7.85
7.83
7.75
7.73
7.71
7.69
7.67
7.65
7.64
7.61
7.56
7.54
7.53
7.52
7.51
7.48
7.46
7.44
7.35
7.33
4.64
4.60
3.80
3.76
2.01
N (t) 7.69
A (s) 9.26
B (s) 8.71
C (d) 8.35 J(8.44)
D (d) 8.11 J(8.41)
E (d) 7.92 J(9.22)
F (d) 7.84 J(8.24)
G (d) 7.74 J(7.86)
H (t) 7.63
I (m) 7.53
J (t) 7.46 J(7.54)
K (d) 7.34 J(9.01)
1.00
1.00
1.00
1.00
2.00
1.00
1.00
1.00
1.00
2.00
1.00
1.00
Chemical Shift (ppm)
1.00
1.00
1.00
1.00
2.00
1.00
1.00
1.00
1.00
2.00
1.00
1.00
1.00
1.00
Chemical Shift (ppm)

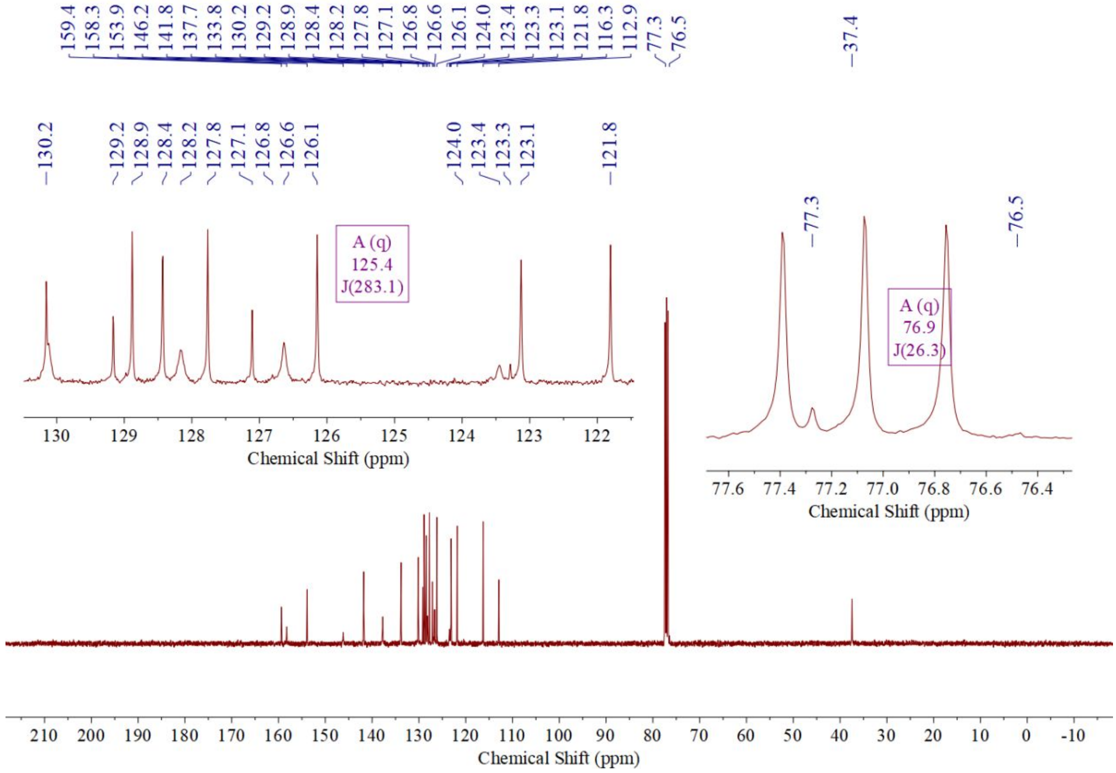
159.4
158.3
153.9
146.2
141.8
137.7
133.8
130.2
129.2
128.9
128.4
128.2
127.8
127.1
126.8
126.6
126.1
124.0
123.4
123.3
123.1
121.8
116.3
112.9
77.3
76.5
37.4
A (q) 125.4 J(283.1)
Chemical Shift (ppm)
A (q) 76.9 J(26.3)
Chemical Shift (ppm)
Chemical Shift (ppm)

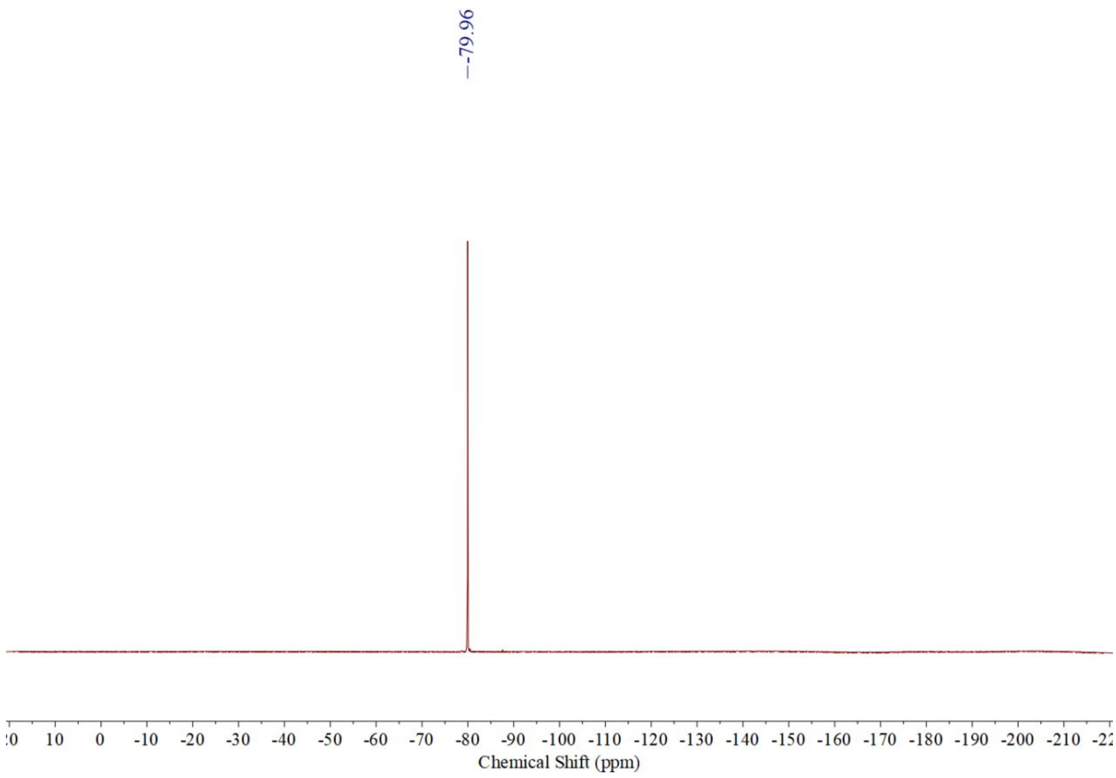

--79.96
Chemical Shift (ppm)

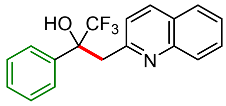

HO
CF3
N

(3ma)

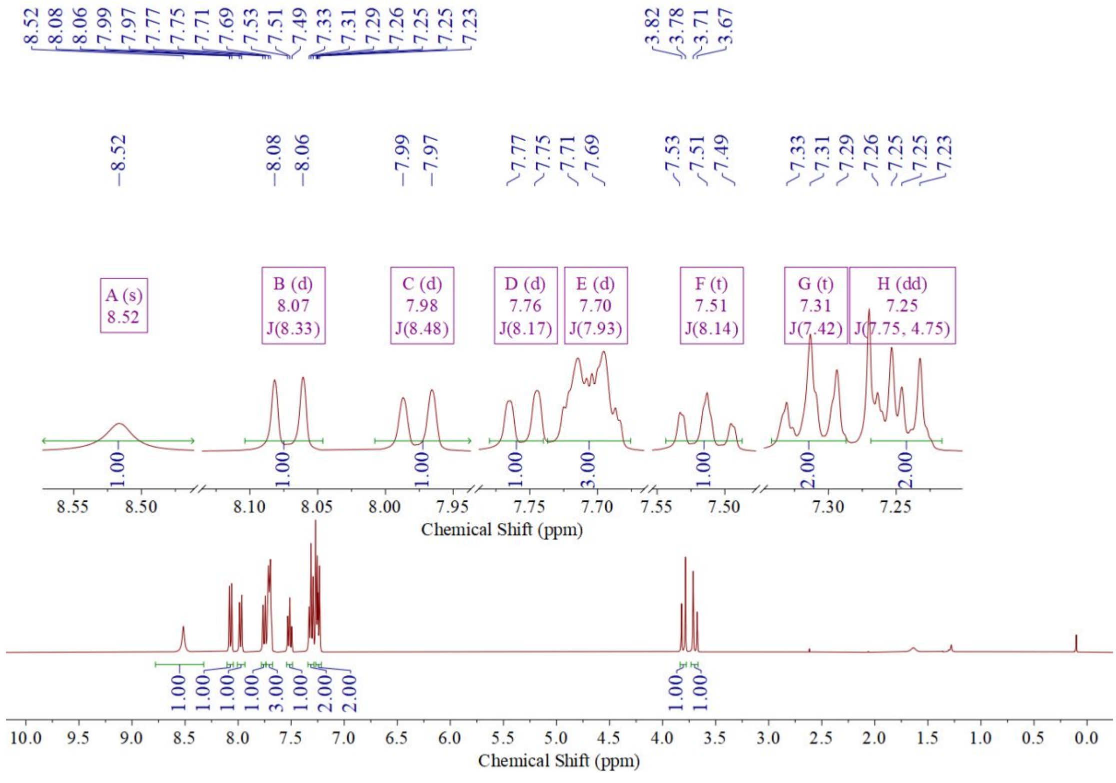

A (s) 8.52
B (d) 8.07 J(8.33)
C (d) 7.98 J(8.48)
D (d) 7.76 J(8.17)
E (d) 7.70 J(7.93)
F (t) 7.51 J(8.14)
G (t) 7.31 J(7.42)
H (dd) 7.25 J(7.75, 4.75)
Chemical Shift (ppm)

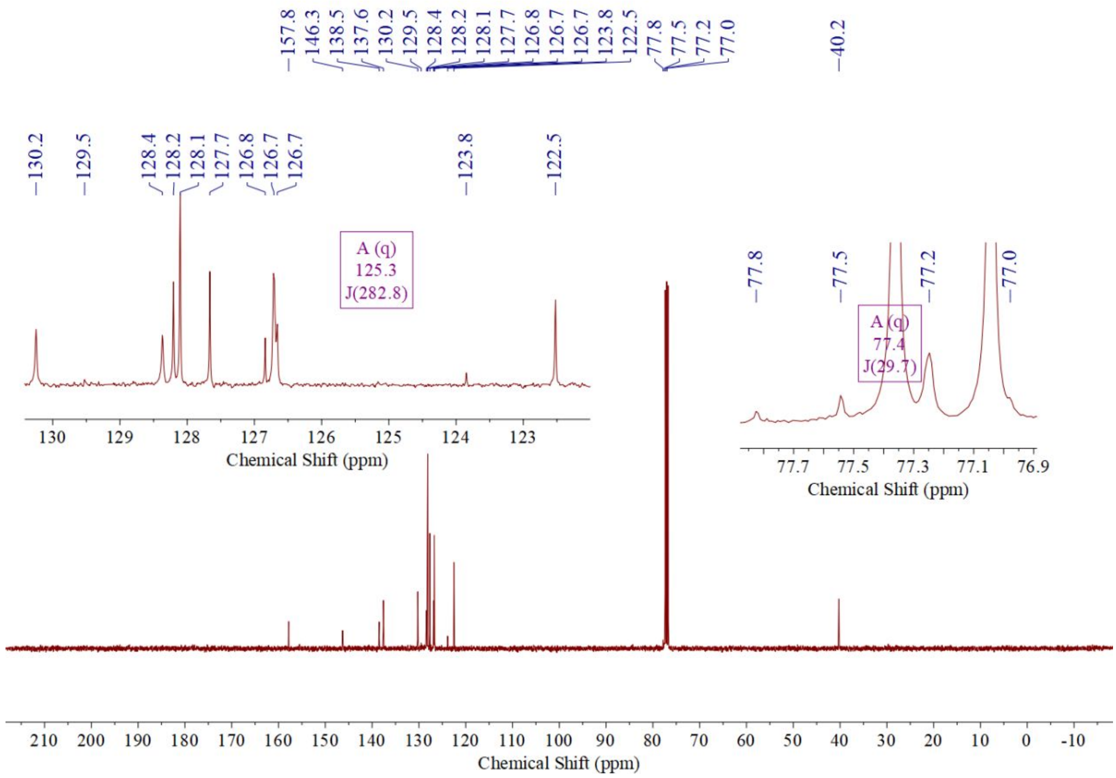
157.8
146.3
138.5
137.6
130.2
129.5
128.4
128.2
128.1
127.7
126.8
126.7
126.7
123.8
122.5
77.8
77.5
77.2
77.0
40.2
A (q)
125.3
J(282.8)
A (q)
77.4
J(29.7)
Chemical Shift (ppm)
Chemical Shift (ppm)
Chemical Shift (ppm)

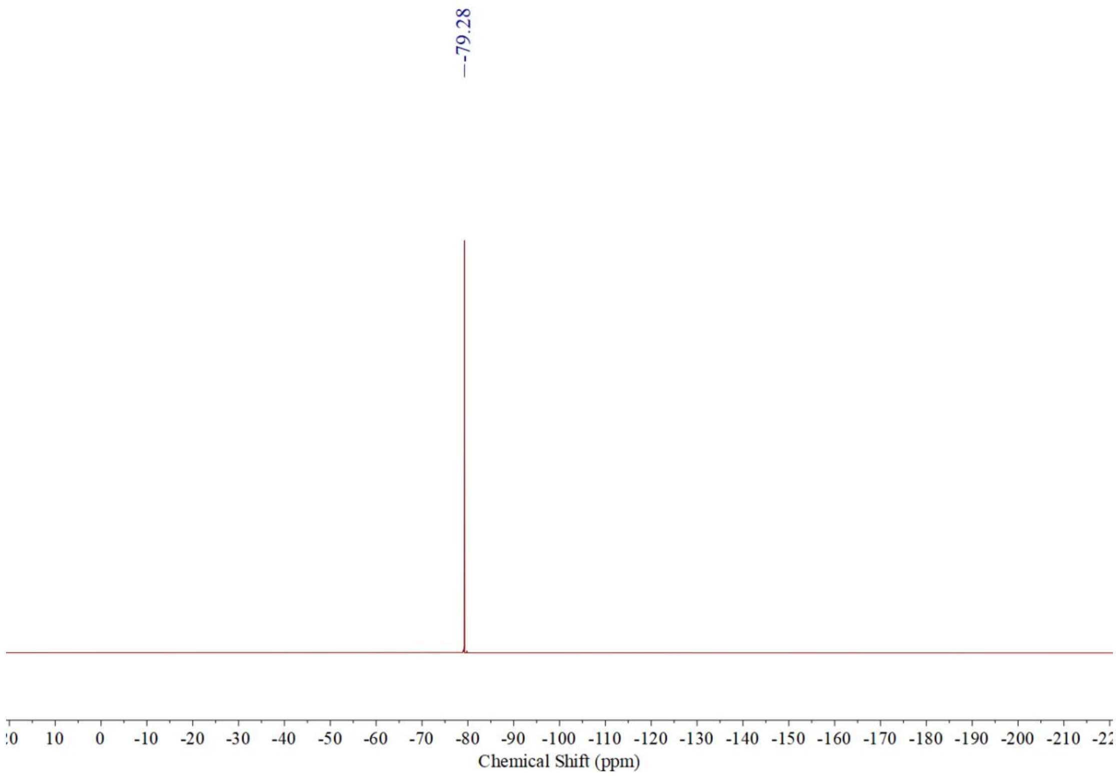
-79.28
Chemical Shift (ppm)

E. HRMS spectra

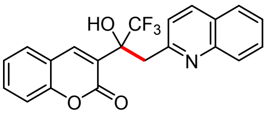

(3aa)

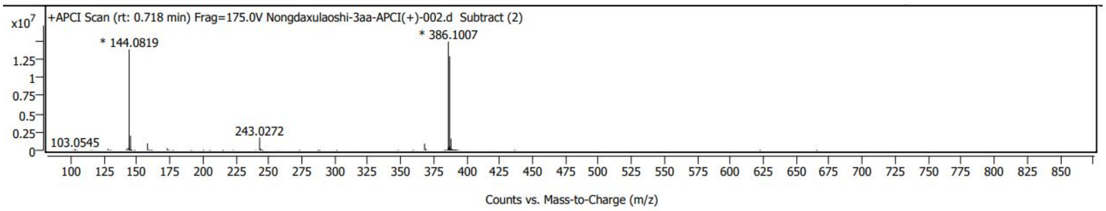

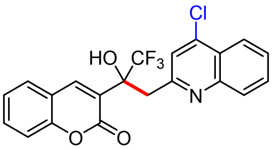

(3ab)

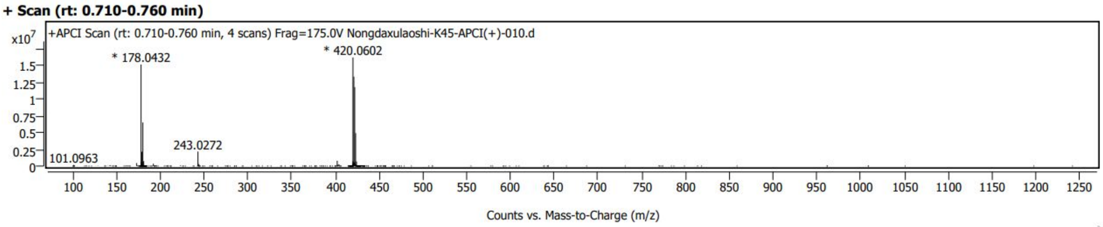

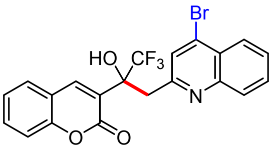

(3ac)

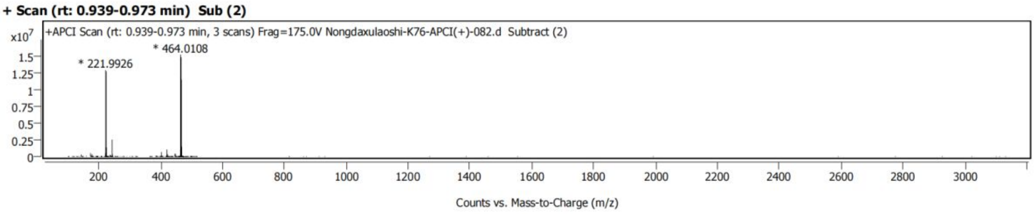

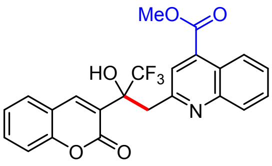

(3ad)

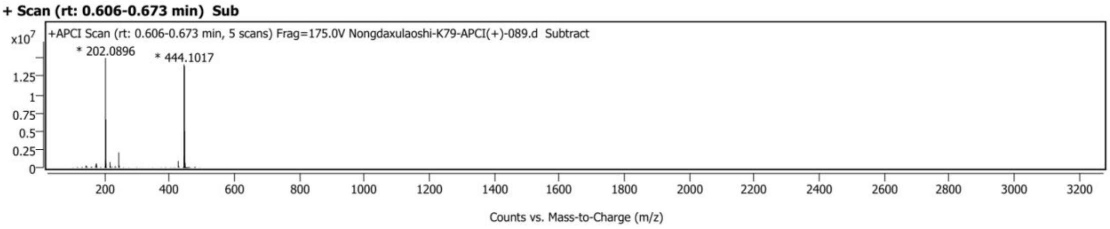

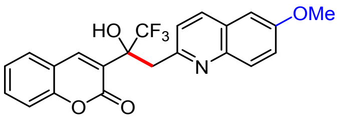

(3ae)

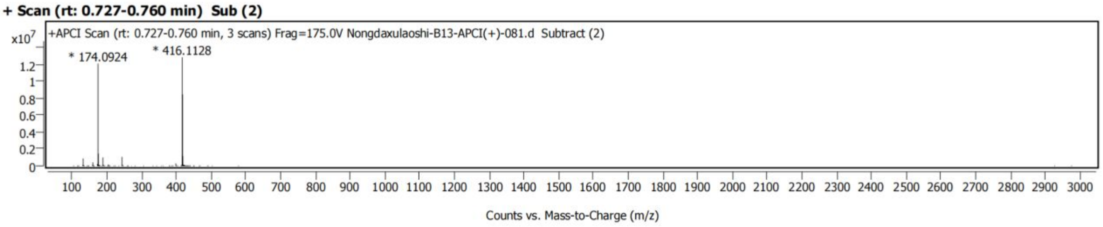

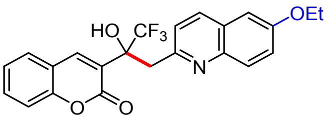

(3af)

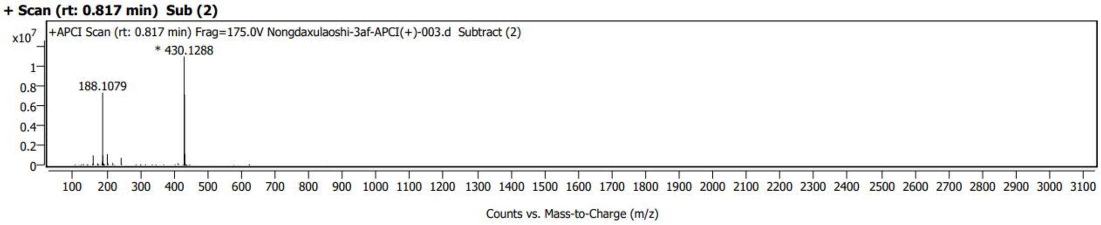

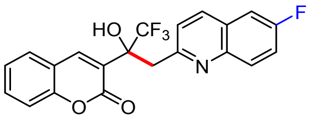

(3ag)

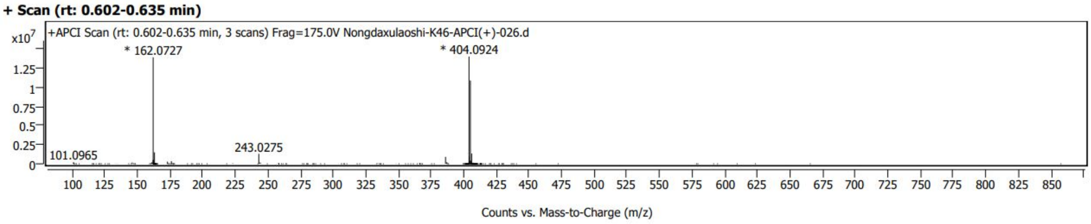

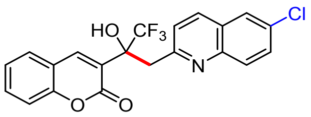

(3ah)

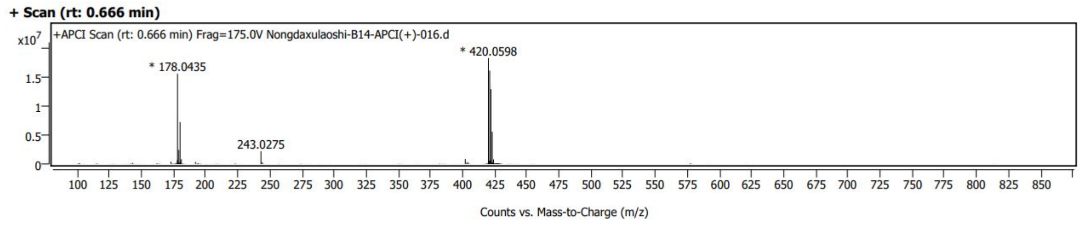
+ Scan (rt: 0.666 min)
+APCI Scan (rt: 0.666 min) Frag=175.0V Nongdaxulaoshi-B14-APCI(+)-016.d
x10^7
* 178.0435
* 420.0598
243.0275
Counts vs. Mass-to-Charge (m/z)

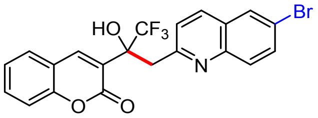
HO
CF3
Br
N
O
O

(3aj)

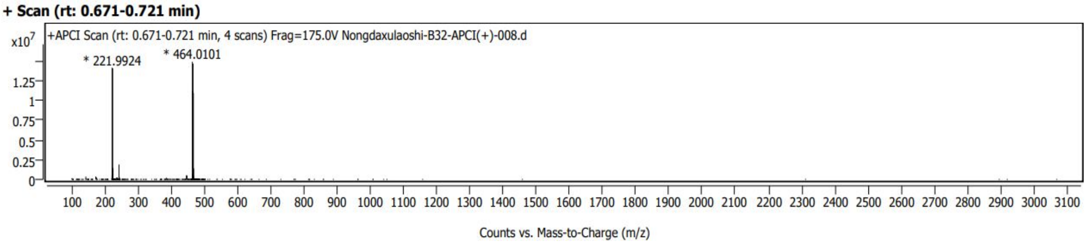
+ Scan (rt: 0.671-0.721 min)
+APCI Scan (rt: 0.671-0.721 min, 4 scans) Frag=175.0V Nongdaxulaoshi-B32-APCI(+)-008.d
x10^7
* 221.9924
* 464.0101
Counts vs. Mass-to-Charge (m/z)

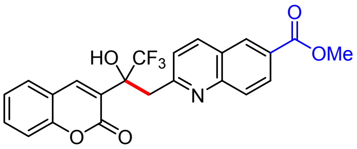
HO
CF3
O
OMe
N
O
O

(3aj)

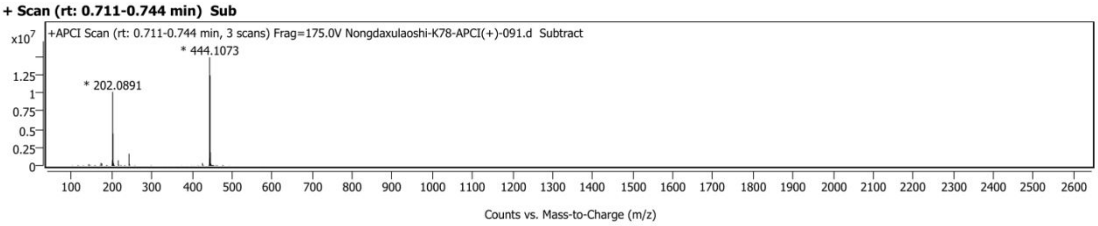
+ Scan (rt: 0.711-0.744 min) Sub
+APCI Scan (rt: 0.711-0.744 min, 3 scans) Frag=175.0V Nongdaxulaoshi-K78-APCI(+)-091.d Subtract
x10^7
* 444.1073
* 202.0891
Counts vs. Mass-to-Charge (m/z)

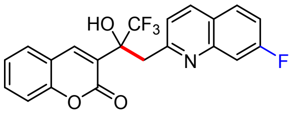
HO
CF3
N
F
O
O

(3ak)

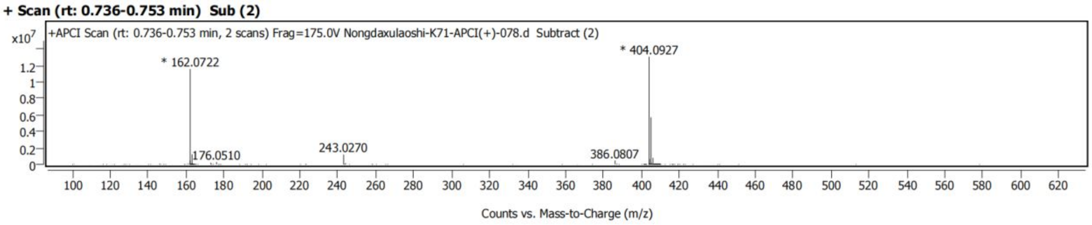
+ Scan (rt: 0.736-0.753 min) Sub (2)
+APCI Scan (rt: 0.736-0.753 min, 2 scans) Frag=175.0V Nongdaxulaoshi-K71-APCI(+)-078.d Subtract (2)
x10^7
* 162.0722
* 404.0927
176.0510
243.0270
386.0807
Counts vs. Mass-to-Charge (m/z)

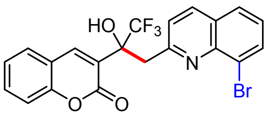
HO
CF3
N
Br
O
O

(3al)

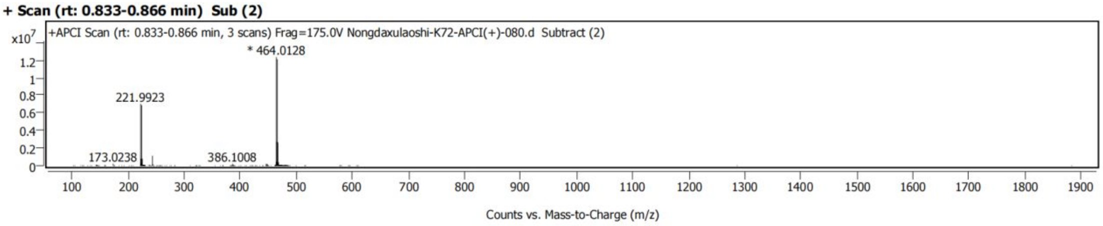

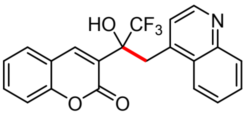

(3am)

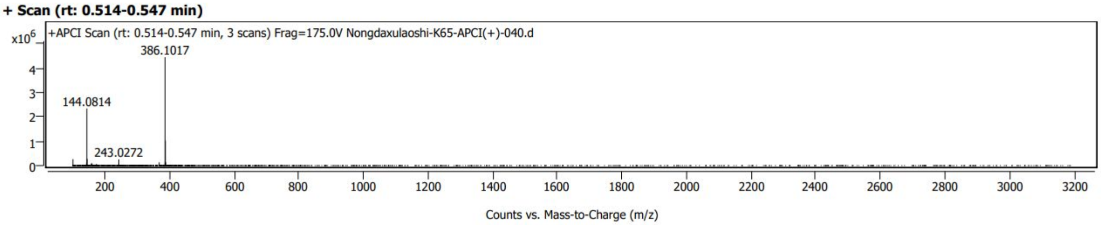

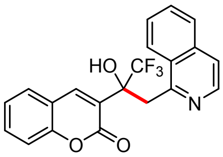

(3an)

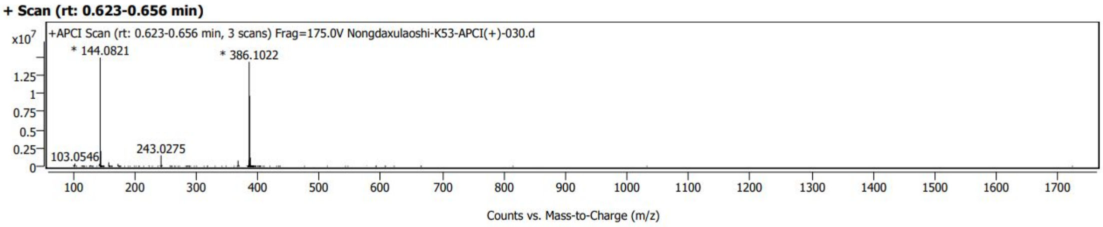

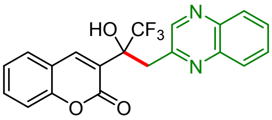

(3ao)

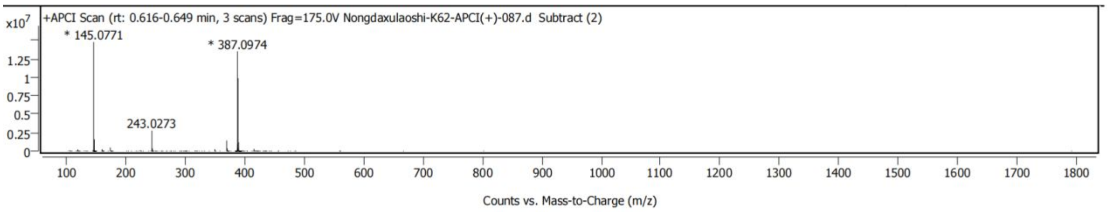

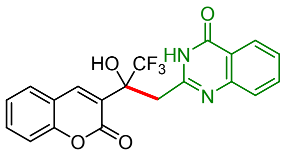

(3ap)

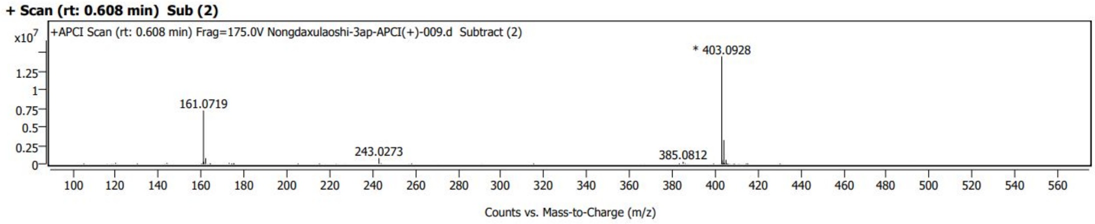

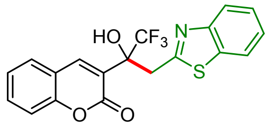

(3aq)

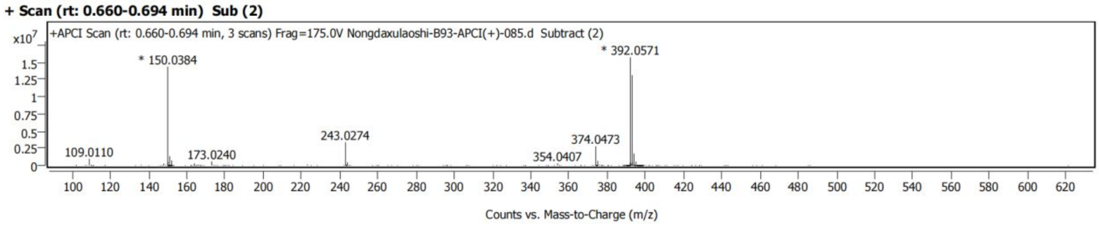

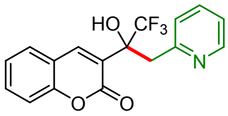

(3ar)

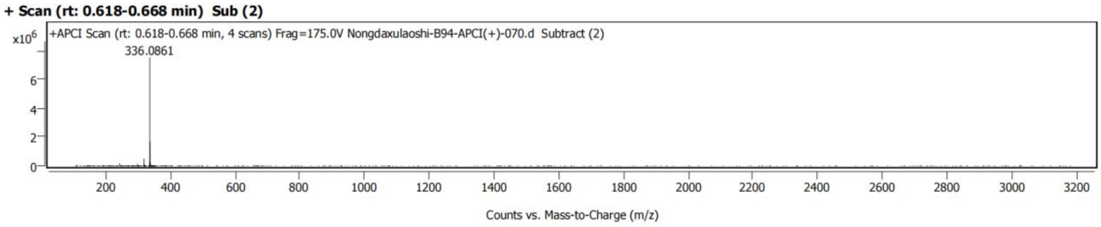

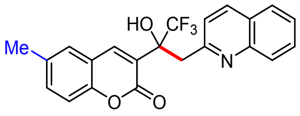

(3ba)

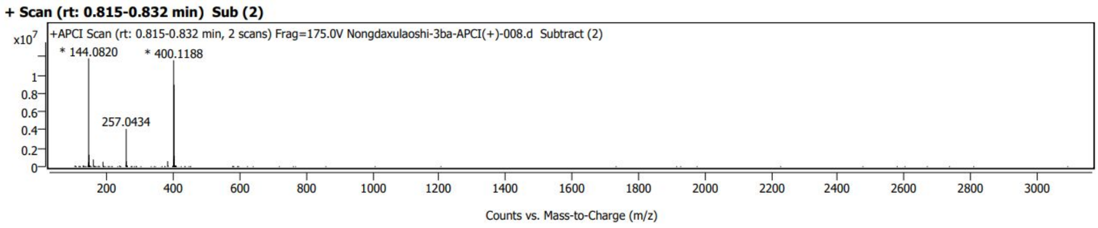

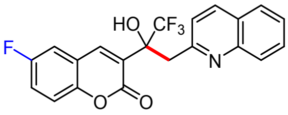

(3ca)

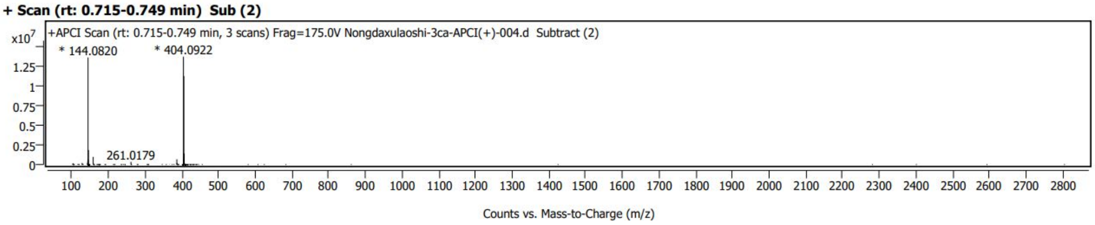

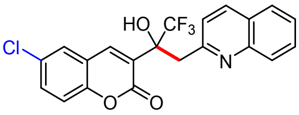

(3da)

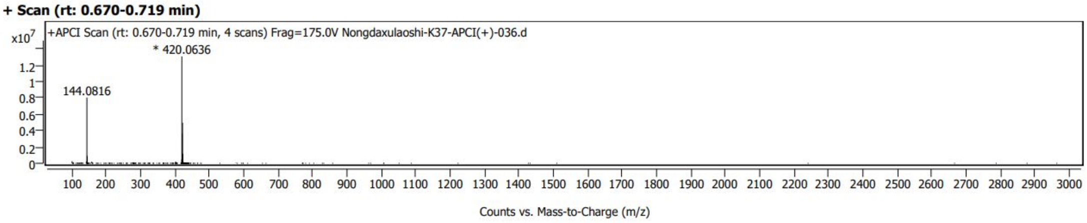

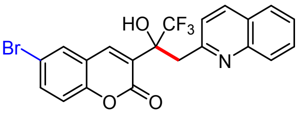

(3ea)

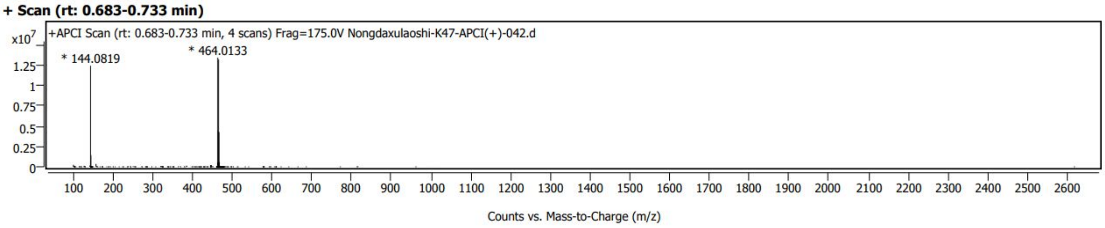

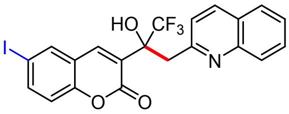

(3fa)

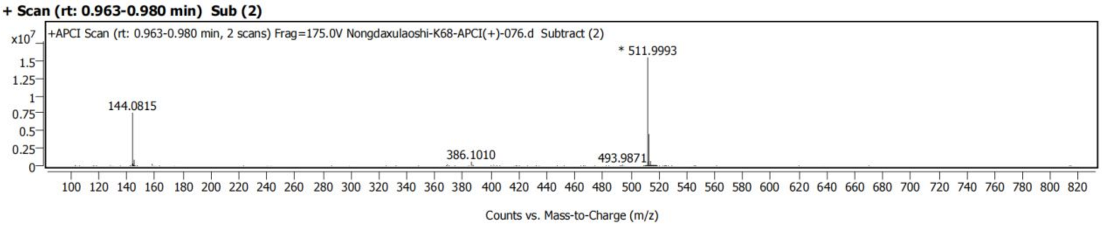

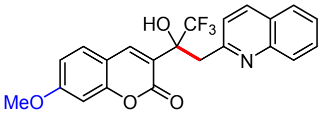

(3ga)

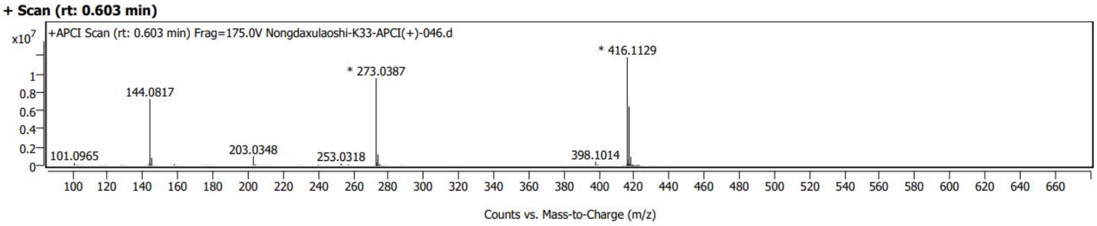

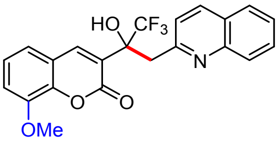

(3ha)

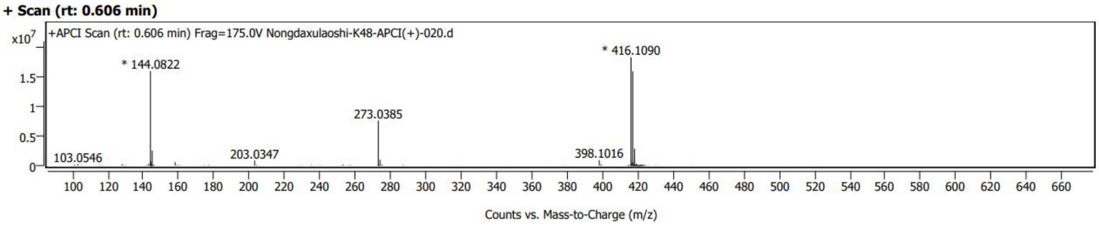

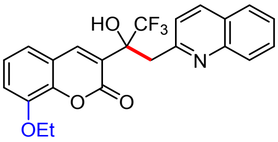

(3ia)

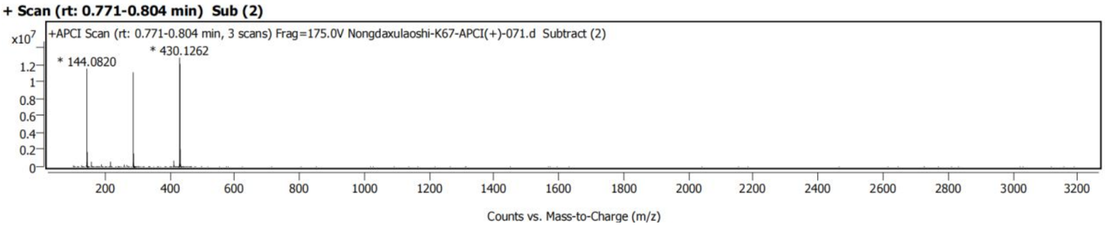

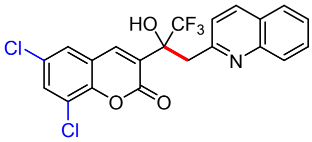

(3ja)

+ Scan (rt: 0.769-0.819 min)

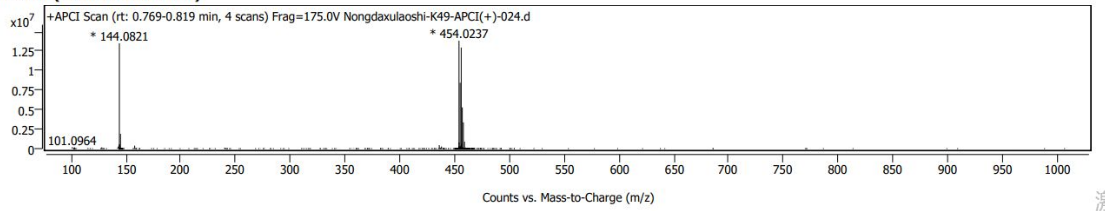

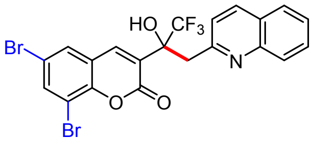

(3ka)

+ Scan (rt: 1.227 min) Sub (2)

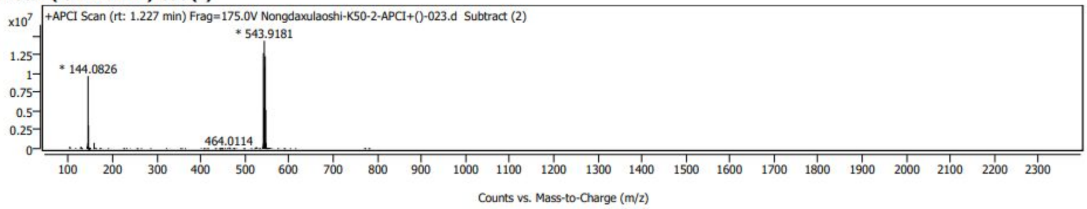

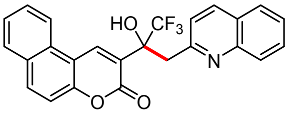

(3la)

+ Scan (rt: 0.743-0.793 min)

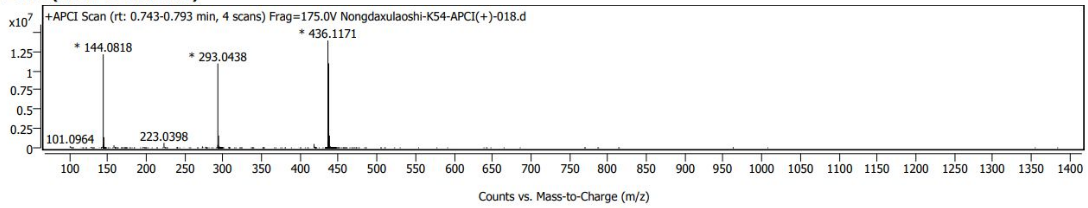

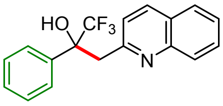

(3ma)

+ Scan (rt: 0.607-0.674 min) Sub (2)

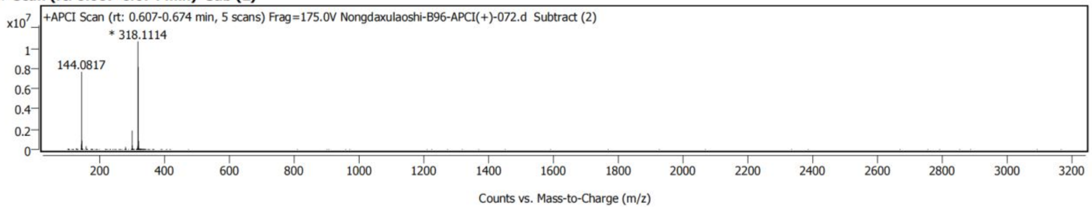

Supplement: Supplementary file 1 — ao2c05855_si_002.pdf [file ao2c05855_si_002.pdf]
